# Controlling Near‐Infrared Fluorescence‐to‐Phosphorescence Ratios and Triplet Lifetimes in Rhodium(I) Dimers via Primary and Secondary Coordination Sphere Effects

**DOI:** 10.1002/anie.6066376

**Published:** 2026-04-09

**Authors:** Vanitha R. Naina, Giacomo Morselli, Alessandro Prescimone, Daniel Häussinger, Oliver S. Wenger

**Affiliations:** ^1^ Department of Chemistry University of Basel Basel Switzerland

**Keywords:** Dinuclear complexes, metal‐metal interactions, near‐infrared emission, triplet lifetimes

## Abstract

Rhodium(I) and other d^8^‐metals form discrete dimers in which bridging ligands position two square‐planar coordination units in close proximity, enabling metal‐metal interactions. Although the metal‐metal distance is known to modulate absorption and photoluminescence wavelengths, clear synthetic guidelines for controlling intersystem crossing and triplet excited‐state lifetimes remain elusive. We show that homoleptic coordination with four identical bridging di‐isocyanide ligands produces a phosphorescent rhodium(I) dimer emitting in the near‐infrared (NIR)‐II region. In contrast, heteroleptic complexes containing two di‐isocyanide and two di‐phosphine ligands yield rhodium(I) dimers that show mainly NIR‐I fluorescence and NIR‐II phosphorescence. In these heteroleptic systems, the fluorescence‐to‐phosphorescence ratio and triplet lifetime depend on the extent of metal‐metal interactions, which can be tuned by over 0.2 Å through modifications at the di‐isocyanide ligand periphery while preserving the primary coordination sphere. Our results are consistent with a picture in which rigidification of the central bimetallic core arises from changes to both the primary and secondary coordination environments, thereby reducing nonradiative excited‐state relaxation pathways, most notably from the phosphorescent T_1_ state. These findings provide guidelines for tuning fluorescence and phosphorescence relevant to imaging and phototherapy, as well as controlling singlet versus triplet photoreactivity in photocatalytic systems for synthetic chemistry and solar energy conversion.

## Graphical abstract

Rh^I^ dimers with bridging aryl isocyanide and di‐phosphine ligands show NIR‐I or NIR‐II fluorescence/phosphorescence, depending on the ligands and conformational rigidity at the Rh_2_ core. Rh‐Rh interactions can be tuned by peripheral modification of the aryl isocyanide ligand, resulting in a rigid Rh_2_ core and improved photophysical properties.

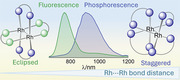

## 1 Introduction

Recent advances in transition‐metal photophysics and photochemistry have focused predominantly on mononuclear compounds [1, 2, 3, 4], while the development of polynuclear systems has lagged behind. This imbalance likely results from the emphasis on making first‐row transition‐metal complexes competitive with mononuclear precious‐metal analogues and on designing photocatalysts optimized for single‐electron‐transfer photoredox or triplet energy transfer catalysis [5, 6, 7, 8, 9]. In such contexts, mononuclear architectures are sufficient, leaving little incentive to explore polynuclear systems.

Nevertheless, cooperative effects between multiple metal centers, which are widely exploited in conventional thermal catalysis [10], could prove equally advantageous in photocatalysis. Such cooperation may enable photoinduced multielectron transfer processes that go beyond conventional single‐electron pathways [11]. In light‐to‐chemical energy conversion and artificial photosynthesis, where multielectron redox events are essential, the ability to transfer multiple redox equivalents per photoexcitation, possibly in a concerted manner that avoids high‐energy radical intermediates, is particularly appealing [12, 13].

Another compelling reason to explore polynuclear complexes is their potential to harness metal‐metal interactions for near‐infrared (NIR) luminescence, a feature that remains challenging to achieve in mononuclear systems [14, 15, 16]. Organometallic compounds that phosphoresce in the NIR region are desirable for photocatalysis [14, 17, 18, 19, 20], bioimaging [21, 22], phototherapy [23, 24, 25, 26], and optoelectronics [27, 28]. However, nonradiative energy dissipation from electronically excited states increases as the energy gap to the ground state decreases [29, 30], rendering the development of efficient red and NIR emitters particularly challenging. Having these two broader research objectives in mind, exploiting metal‐metal cooperativity for multielectron transfer and for NIR luminescence, we identified square‐planar d^8^ metal complexes as a promising class of compounds worthy of more detailed investigation.

Complexes featuring d^8^‐d^8^ interactions often exhibit low‐energy absorption arising from short metal‐metal contacts [31, 32, 33, 34, 35, 36, 37], which can be tuned to achieve red or NIR emission through rational design [38]. Since the early reports on the photophysical behavior of pyrophosphito‐bridged Pt^II^ dimers [39, 40], extensive studies have sought to elucidate the nature of these metal‐metal interactions and their influence on excited‐states [38, 41, 42, 43] and intersystem crossing (ISC) pathways [44, 45, 46]. As a result, Pt^II^ dimers have emerged as a versatile class of chromophores with applications spanning photocatalysis [47] and OLEDs [48, 49]. Encouraged by these discoveries, analogous Pd^II^ and Ni^II^ dimers have also been investigated to probe the role of metal‐metal interactions in modulating excited‐state properties, with some of these complexes finding use in photocatalysis [50, 51, 52, 53].

Alongside the well‐studied Pt^II^ dimers, rhodium dimers with the individual metals in 0, +1, and +2 oxidation states also represent an interesting class of compounds, not only for their photophysical properties but also for their ability to generate H_2_ from hydrohalic acids (HX) [54, 55, 56, 57, 58, 59, 60, 61]. Among these, Rh in the +1 oxidation state coordinated to isocyanide ligands adopts a square‐planar geometry and exhibits metallophilic Rh···Rh interactions. The color and stacking effects of these monomers vary with the isocyanide substituents [62], counteranions [63, 64], and crystallization conditions [65, 66], which in turn reflect subtle variation in the Rh^I^···Rh^I^ distances.

Several homoleptic Rh^I^ dimers with bridging isocyanides featuring short metal‐metal contacts (∼3.2 Å) were reported already in the 1970s [55, 67] and established their photoreactivity towards HX to produce H_2_ [54]. The complexes exhibit both fluorescence and phosphorescence [68, 69, 70, 71]. Other studies developed heteroleptic Rh^I^ complexes incorporating di‐phosphine or di‐arsine bridges, which displayed fluorescence at room temperature and phosphorescence only at 77 K [72, 73, 74, 75]. Later, a heteroleptic complex using 2,2‐dimethyl‐1,3‐diisocyanopropane (dmb) and diphenylphosphinomethane (dppm), [Rh(dmb)_2_(dppm)_2_]^2+^, was reported, featuring a particularly short intramolecular Rh···Rh distance (3.037 Å) and fluorescence centered at 710 nm [71]. Among numerous studies on heteroleptic systems, a rare investigation of triplet excited‐state properties using transient absorption (TA) spectroscopy at room temperature was reported; however, phosphorescence was not observed for these systems, despite the short Rh^I^···Rh^I^ distances [76].

Exploiting aggregation‐induced metallophilic interactions is an alternative approach to achieving short Rh^I^···Rh^I^ contacts, but usually leads to extended supramolecular structures rather than discrete dimers [77, 78, 79, 80]. Several recent studies have reported NIR‐II emitting Rh^I^ aggregates for applications in bio‐imaging [81, 82], as well as Rh^I^ complexes showing NIR‐II phosphorescence in the solid state [83, 84]. However, such systems offer limited control over metal‐metal distances, hindering precise tuning of structure‐property relationships. Thus, discrete Rh^I^ dimers exhibiting NIR‐II phosphorescence in solution still remain rare, and synthetic guidelines to obtain such compounds are lacking.

Against this background, the aim of this study was twofold: first, to gain clearer insight into how NIR phosphorescence can be reliably installed in d^8^ metal dimers; and second, to understand which structural factors promote intersystem crossing and control the resulting triplet excited state lifetimes. Ultimately, we sought to achieve full synthetic control over the photophysical properties of d^8^ metal dimers, similar to what is now readily possible with many mononuclear transition metal complexes [2]. This study comprises two parts. In the first, we synthesized both a homoleptic and a heteroleptic Rh^I^ complex in order to elucidate the fundamental differences between a coordination environment composed solely of isocyanides and one containing both isocyanides and phosphines. The homoleptic complex exhibits pronounced NIR‐II phosphorescence, whereas the heteroleptic analogue displays dominant NIR‐I fluorescence accompanied by weak NIR‐II phosphorescence. Ultrafast TA spectroscopy hints at distinct ISC pathways in homo‐ and heteroleptic compounds. In the second part, we developed a peripheral ligand‐modification strategy for heteroleptic systems, affording a series of structurally analogous complexes with Rh^I^···Rh^I^ separations varied by over 0.2 Å, thereby enabling modulation of fluorescence‐to‐phosphorescence ratios within the NIR‐II region. The obtained insights are relevant not only for tuning luminescence properties, but also for achieving control over singlet and triplet excited‐state reactivity, which is important for modern photocatalysis [85, 86, 87].

## 2 Results and Discussion

### 2.1 Structural and Electronic Differences Between Homo‐ and Heteroleptic Complexes

**FIGURE 1:**
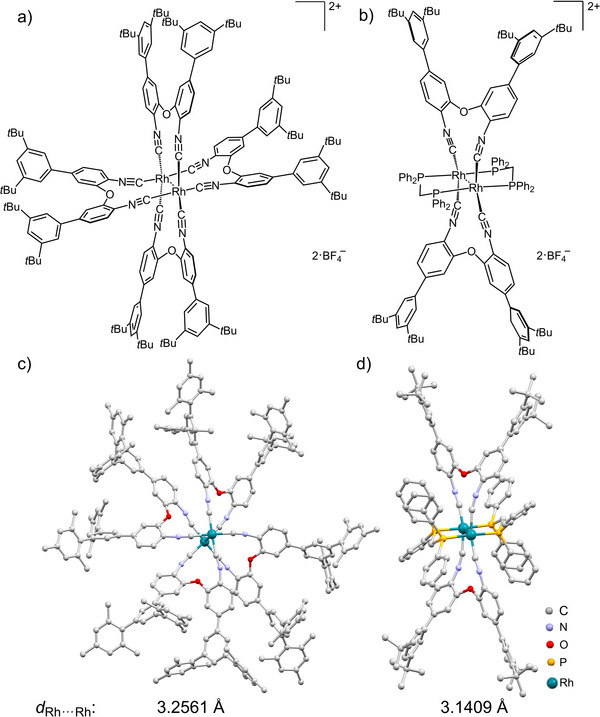
Molecular structures of (a) the homoleptic **[Hom‐Rh_2_]*^t^* ^Bu^** and (b) the heteroleptic **[Het‐Rh_2_]*^t^* ^Bu^** complexes investigated in this work. Molecular structures of (c) homoleptic **[Hom‐Rh_2_]^Mes^** and (d) heteroleptic **[Het‐Rh_2_]*^t^* ^Bu^** complexes in the solid state, along with Rh^I^···Rh^I^ distances. The **[Hom‐Rh_2_]*^t^* ^Bu^** complex could not be crystallized. We used the x‐ray crystal structure of **[Hom‐Rh_2_]^Mes^** as a proxy to estimate the Rh^I^···Rh^I^ distance in **[Hom‐Rh_2_]*^t^* ^Bu^** (see text for further information). Counter‐anions, hydrogen atoms, and non‐coordinating solvent molecules are omitted for clarity. Structural parameters are given in the Supporting Information (Figures S62 and S63; Tables S1 and S2).

The bridging aryl di‐isocyanide ligand **L*^t^* ^Bu^** was synthesized from 2,2''‐oxydianiline via a multistep synthetic route and designed to support short metal‐metal contacts (molecular structure in the solid state is given in Figure S61). The homoleptic complex **[Hom‐Rh_2_]*^t^* ^Bu^** (Figure 1a) was readily obtained by reacting **L*^t^* ^Bu^** with [Rh(cod)_2_BF_4_] in a 2:1 ratio, and its identity was confirmed via nuclear magnetic resonance (NMR) spectroscopy, high‐resolution electron spray ionization mass spectrometry (HR‐ESI‐MS), infrared spectroscopy (IR) and elemental analysis. However, attempts to obtain single crystals of **[Hom‐Rh_2_]*^t^* ^Bu^** for determining the Rh^I^···Rh^I^ distance were unsuccessful.

**FIGURE 2:**
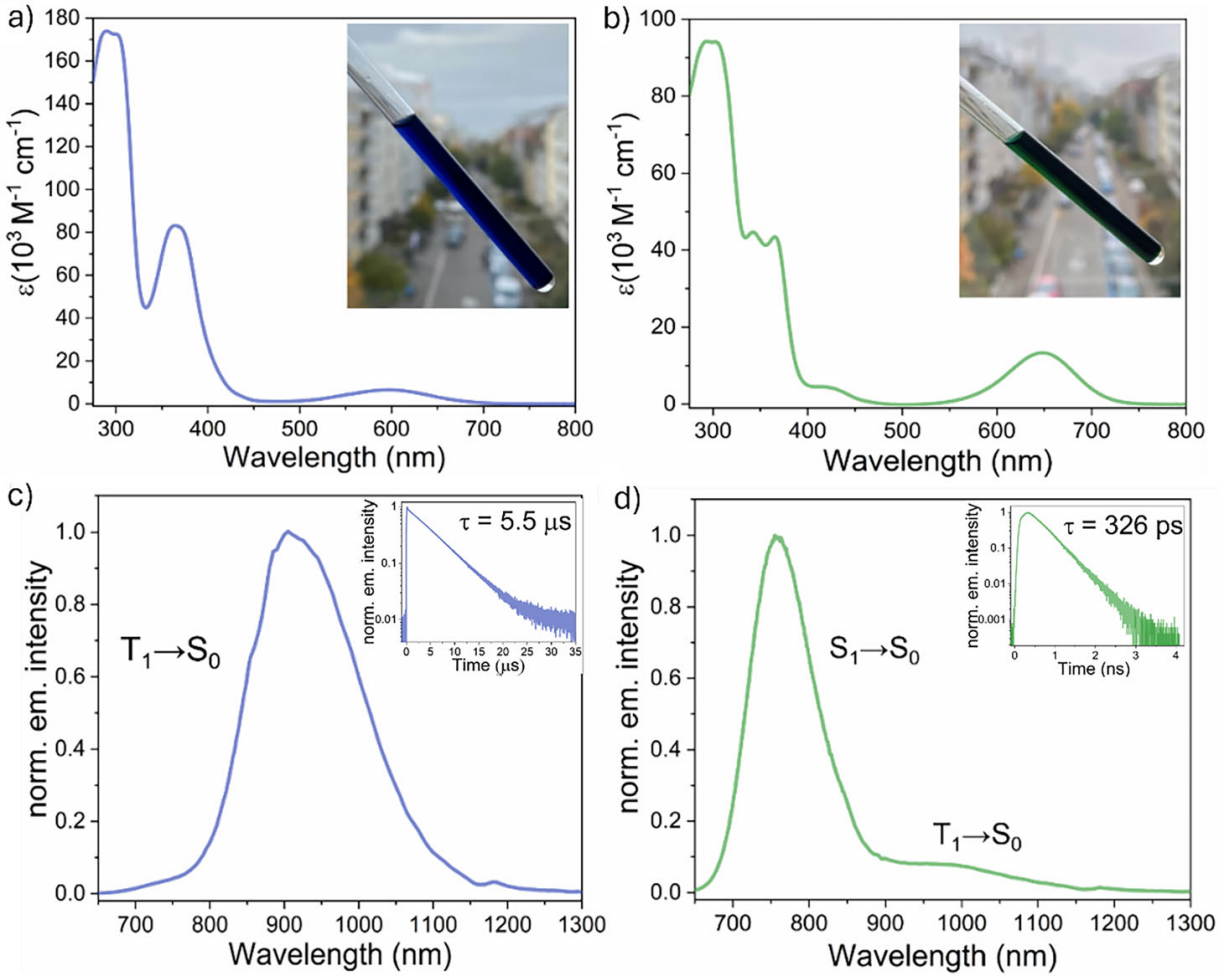
(a) UV‐Vis absorption spectrum of **[Hom‐Rh_2_]*^t^* ^Bu^**. Inset: A picture of an NMR tube containing a solution of **[Hom‐Rh_2_]*^t^* ^Bu^**. (b) UV‐Vis absorption spectrum of **[Het‐Rh_2_]*^t^* ^Bu^**. Inset: A picture of an NMR tube containing a solution of **[Het‐Rh_2_]*^t^* ^Bu^**. (c) Steady‐state photoluminescence spectrum of **[Hom‐Rh_2_]*^t^* ^Bu^** showing phosphorescence from the T_1_ excited state. Inset: Phosphorescence decay of **[Hom‐Rh_2_]*^t^* ^Bu^** after pulsed excitation at *λ*_exc_ =  625 nm and detected at *λ*_obs_ = 790 nm. (d) Steady‐state photoluminescence spectrum of **[Het‐Rh_2_]*^t^* ^Bu^** showing predominant fluorescence from the S_1_ excited‐state, along with some weaker phosphorescence from the T_1_ state. Inset: Fluorescence decay (*λ*_exc_ = 635 nm) of **[Het‐Rh_2_]*^t^* ^Bu^** detected at *λ*_obs_ = 725 nm obtained from time‐correlated single‐photon counting (TCSPC) measurements. The decay of the T_1_ excited‐state is analyzed below in the nanosecond‐transient absorption studies. All measurements were performed in deoxygenated CH_2_Cl_2_ at room temperature.

To enable structural comparison between homoleptic and heteroleptic complexes, an analogous ligand **L^Mes^** was designed and used to synthesize **[Hom‐Rh_2_]^Mes^**. Single crystal x‐ray diffraction (SCXRD) analysis revealed a paddlewheel‐type Rh_2_ core with a Rh^I^···Rh^I^ distance of 3.2561(11) Å in the solid state (Figure 1c). Four bridging **L^Mes^** ligands are arranged as a four‐blade propeller about the Rh^I^···Rh^I^ axis, resulting in a partially staggered conformation of two [Rh(CNR)_4_]^+^ units (Figure 1c). The observed Rh^I^···Rh^I^ distance falls within the range reported for alkylisocyanide‐bridged Rh^I^ dimers [67, 71]. Since the low‐energy absorption bands of **[Hom‐Rh_2_]*^t^* ^Bu^** and **[Hom‐Rh_2_]^Mes^** are similar (Figure 2a and S71), the Rh^I^···Rh^I^ distance in **[Hom‐Rh_2_]*^t^* ^Bu^** is expected to be comparable. Here, and in later sections of this paper, we use the x‐ray crystallographic data described above (150 K) and the observed structural differences between compounds to identify structure‐function relationships, particularly for UV–Vis absorption and photoluminescence at room temperature. The metal‐metal distance is considered a key structural parameter. Because the crystallographic and spectroscopic measurements were performed at 150 K and 300 K, respectively, the analysis assumes negligible changes in metal‐metal distances between these temperatures.

The heteroleptic complex **[Het‐Rh_2_]*^t^* ^Bu^** (Figure 1b) was obtained by reacting **L*^t^* ^Bu^** and dppm with [Rh(cod)_2_BF_4_] in a 1:1:1 ratio. SCXRD analysis revealed a lantern‐type Rh_2_ core bridged by two trans **L*^t^* ^Bu^** and two trans dppm ligands with a Rh^I^···Rh^I^ distance of 3.1409(2) Å (Figure 1d), about 0.1 Å shorter than in the homoleptic **[Hom‐Rh_2_]^Mes^** complex, attributable to the short and rigid backbone of the dppm ligand. Further, in contrast to **[Hom‐Rh_2_]^Mes^**, the two [Rh(CNR)_2_(P)_2_]^+^ units of **[Het‐Rh_2_]*^t^* ^Bu^** are arranged in an eclipsed (rather than partially staggered) conformation about the Rh^I^···Rh^I^ axis (Figure 1d, Figure S66). The molecular ion peak of **[Hom‐Rh_2_]*^t^* ^Bu^** appears at m/z  =  1295.6588 ([C_168_H_192_N_8_O_4_Rh_2_]^2+^) and an additional peak related to the ion pair with a tetrafluoroborate at m/z  =  2677.8140 ([C_168_H_192_N_8_O_4_Rh_2_BF_4_]^+^) (Figures S40 and S41). Likewise, the doubly charged molecular ion peak of **[Het‐Rh_2_]*^t^* ^Bu^** is observed at m/z  =  1083.4034 ([C_134_H_140_N_4_O_2_P_4_Rh_2_]^2+^) and a corresponding ion pair at m/z  =  2252.8115 ([C_134_H_140_N_4_O_2_Rh_2_BF_4_]^+^) (Figures S42 and S43).

Additionally, **[Het‐Rh_2_]*^t^* ^Bu^** displays a complex splitting pattern with more than 16 lines in the ^31^P{^1^H} NMR spectrum. These lines represent the AA'A″A‴ part of the high‐order AA'A″A‴XX′ spin system resulting from two magnetically non‐equivalent Rh and four magnetically non‐equivalent P nuclei at the Rh_2_ core (Figure S11). Collectively, these results suggest that both **[Hom‐Rh_2_]*^t^* ^Bu^** and **[Het‐Rh_2_]*^t^* ^Bu^** retain their dimeric structure in solution, and stacking effects leading to higher‐order species, such as trimer or tetramer, can be excluded. These structural insights enable a direct comparison of the effects of homoleptic versus heteroleptic coordination on the photophysical properties of the Rh_2_ core.

In the IR spectrum of **[Hom‐Rh_2_]*^t^* ^Bu^**_,_ the ν(CN) band appears at 2146 cm^−1^, shifted to a higher wavenumber relative to the free ligand **L*^t^* ^Bu^** (2118 cm^−1^), reflecting weak π‐backbonding to the isocyanide ligands from comparatively electron‐poor Rh^I^ centers. In contrast, the stronger σ‐donating ability of the dppm ligands [88, 89] in **[Het‐Rh_2_]*^t^* ^Bu^** renders the Rh^I^ centers more electron‐rich and thereby enhances π‐backbonding to the isocyanide ligands, resulting in a shift of the ν(CN) band to lower energy (2111 cm^−1^) relative to the free ligand (Figures S55–S57). The differences in the π‐backbonding are also reflected in the C≡N bond distances. In the homoleptic complex, C≡N distances range from 1.10 to 1.14 Å, whereas longer C≡N bonds of ∼1.17 Å are observed in the heteroleptic complex. Electrochemical data further support differences in electron density at the Rh_2_ core, with oxidation potentials of *E*_pa_ =  0.61 V for **[Hom‐Rh_2_]*^t^* ^Bu^** and *E*_1/2_ = 0.73 V versus SCE for **[Het‐Rh_2_]*^t^* ^Bu^** (Figure S96a,b).

The electrochemical potentials for the Rh(I) to Rh(II) oxidation in our complexes, as well as the reversibility of the corresponding redox waves in cyclic voltammetry, appear to be governed by a complex interplay between the relative stabilities of the different oxidation states and the reorganization energy associated with the oxidation process. We report the respective cyclic voltammetry data in the Supporting Information for completeness (Figure S96), but a detailed interpretation and discussion are beyond the scope of this work.

### 2.2 Achieving Control Over Fluorescence‐to‐Phosphorescence Ratios and NIR‐II Versus NIR‐I Emission

**[Hom‐Rh_2_]*^t^* ^Bu^** and **[Het‐Rh_2_]*^t^* ^Bu^** are deep‐blue and dark green colored complexes, respectively, and accordingly exhibit strong absorptions in the visible region (Figure 2a,b). **[Hom‐Rh_2_]*^t^* ^Bu^** shows a broad absorption band between 500 and 700 nm (ε_595 nm_  =  6550 M^−1^ cm^−1^), along with two intense bands at 364 and 290 nm (ε > 8 × 10^4^ M^−1^ cm^−1^) in CH_2_Cl_2_. The low‐energy band is characteristic of Rh^I^ dimers and is assigned to allowed singlet‐singlet 4dσ*(Rh)→5pσ(Rh)/π*(isocyanide) transitions [71, 90, 91], where dσ* results from the antibonding overlap between (occupied) d_z_^2^ orbitals [92]. The 5pσ orbital results from a bonding interaction between the (vacant) 5p_z_ orbitals of Rh, which mixes with low‐lying (vacant) π* orbitals of the isocyanide ligand [71, 90, 91, 93].

In **[Het‐Rh_2_]*^t^* ^Bu^**, the corresponding low‐energy absorption band appears between 550 and 750 nm (*ε*_650 nm_  =  13300 M^−1^ cm^−1^) (Figure 2b) in CH_2_Cl_2_, representing a substantial red‐shift of ∼0.2 eV relative to **[Hom‐Rh_2_]*^t^* ^Bu^** (Figure 2a). This shift is consistent with a shorter Rh^I^···Rh^I^ distance in the heteroleptic complex, which enhances orbital interactions between the metal centers [71]. Although the Rh^I^···Rh^I^ distances are comparable to those of previously reported alkyl isocyanide Rh^I^ dimers, the low‐energy band in the present complexes is noticeably red‐shifted [67, 68, 71]. This is likely attributable to increased conjugation in the aryl isocyanide ligand, which lowers the energy of the relevant π* orbital. The 4dσ*(Rh)→5pσ(Rh)/π*(isocyanide) absorption band exhibits a strong dependence on the solvent, a behavior consistent with that of previously reported dinuclear complexes (Figure S67) [94].

In dichloromethane, both complexes are emissive, but their excited‐state behavior differs significantly (Figure 2c,d). **[Hom‐Rh_2_]^*t*Bu^** displays a predominant phosphorescence band in the NIR‐II region centered at 900 nm upon excitation at 585 nm. The emission band originates from a triplet state, as indicated by its long lifetime (*τ*_T1_ = 5.5 µs) and the comparatively large apparent Stokes shift relative to the singlet‐singlet absorption band maximum. The phosphorescence is quenched efficiently in the presence of O_2_ (Figure S91).

The phosphorescence quantum yield (*ϕ*_ph_) of 8.0 % (in deoxygenated CH_2_Cl_2_ at room temperature) is relatively high for NIR‐II emission. A very weak fluorescence band at 745 nm is also observed (Figure S69a), however, its kinetics could not be resolved by TCSPC. This short‐lived component was resolved using TA measurements with higher time resolution, as described in the following section. Such high *ϕ*_ph_ and phosphorescence‐to‐fluorescence ratios are rarely reported and observed only in stacked systems rather than discrete dimers [82].

In contrast, **[Het‐Rh_2_]*^t^* ^Bu^** exhibits dual luminescence upon excitation at 650 nm, featuring a major fluorescence band centered at 750 nm and a shoulder in the NIR‐II region at ∼950 nm (Figure 2d). The fluorescence lifetime (*τ*_S1_) and quantum yield (*ϕ*_fl_) were measured to be 326 ps (using a reconvolution fit) and 0.7 %, respectively, while the phosphorescence quantum yield (*ϕ*_ph_) is 0.2 % in deoxygenated CH_2_Cl_2_ at room temperature. To the best of our knowledge, NIR‐II phosphorescence has not been reported previously for heteroleptic Rh^I^ complexes [95]. Although direct measurement of the phosphorescence lifetime was not feasible due to instrumental limitations, it was determined via nanosecond TA spectroscopy, as discussed in the following section. These findings show that modulation of the ligand environment at the Rh_2_ core enables control over the fluorescence to phosphorescence ratio, while also facilitating emission in the NIR‐I and NIR‐II windows with significant quantum efficiencies.

### 2.3 Relaxation Pathways and Fluorescence‐to‐Phosphorescence Ratios in Homo‐ and Heteroleptic Complexes

**FIGURE 3:**
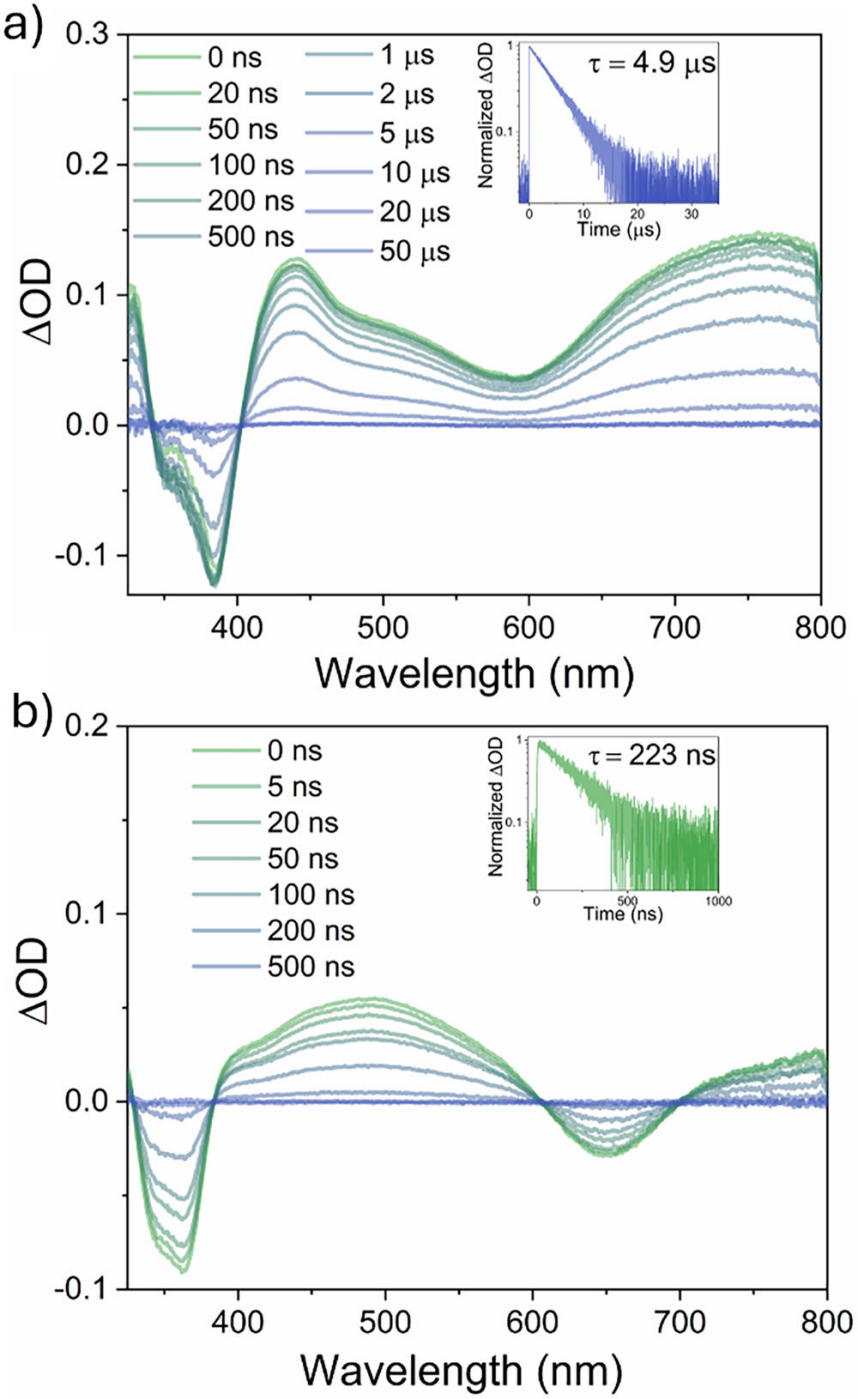
(a) Nanosecond transient UV–Vis absorption spectra at different delay times for **[Hom‐Rh_2_]*^t^* ^Bu^** (laser pump pulses at 600 nm, 8 mJ/pulse, OD_600 nm_ = 0.2, and integration time of 200 ns). Inset: decay of the excited‐state absorption (ESA) at 750 nm. (b) Transient UV–Vis absorption spectra at different delay times for **[Het‐Rh_2_]*^t^* ^Bu^** (laser pump pulses at 625 nm, 8 mJ/pulse, OD_625 nm_  =  0.2, and integration time of 200 ns). Inset: decay of the ESA at 450 nm. All measurements were performed in deoxygenated CH_2_Cl_2_ at room temperature.

The nanosecond TA spectrum of **[Hom‐Rh_2_]*^t^* ^Bu^**, collected upon photoexcitation at 600 nm, shows a ground‐state bleach (GSB) at ∼390 nm and strong excited‐state absorption (ESA) spanning the visible region at early time delays (Figure 3a). Kinetic analysis of the time‐dependent evolution of the GSB and ESA signals yields an excited‐state lifetime of 4.9 µs. Taken together, the mono‐exponential luminescence decay profile (Figure 2c) and TA‐derived kinetics (Figure 3a) confirm that the ESA is attributable to electronic transitions originating from the emissive triplet state.

For **[Het‐Rh_2_]*^t^* ^Bu^**, photoexcitation at 625 nm, two strong GSB at ∼370 nm and ∼650 nm, along with ESA at ∼500 nm and ∼750 nm, are observed as prominent features (Figure 3b). Time‐resolved monitoring of these signals reveals a mono‐exponential decay with a lifetime of 223 ns, which is attributed to the phosphorescence observed in the photoluminescence spectrum of **[Het‐Rh_2_]*^t^* ^Bu^** (Figure 2d). The short fluorescence lifetime of 326 ps could not be resolved under nanosecond experimental conditions.

Additional visible TA spectroscopic measurements on the sub nanosecond time scale provide insights into the distinct excited‐state relaxation pathways observed for **[Hom‐Rh_2_]*^t^* ^Bu^** and **[Het‐Rh_2_]*^t^* ^Bu^**. The data were analyzed using a global fitting method (Figures S94 and S95).

**FIGURE 4:**
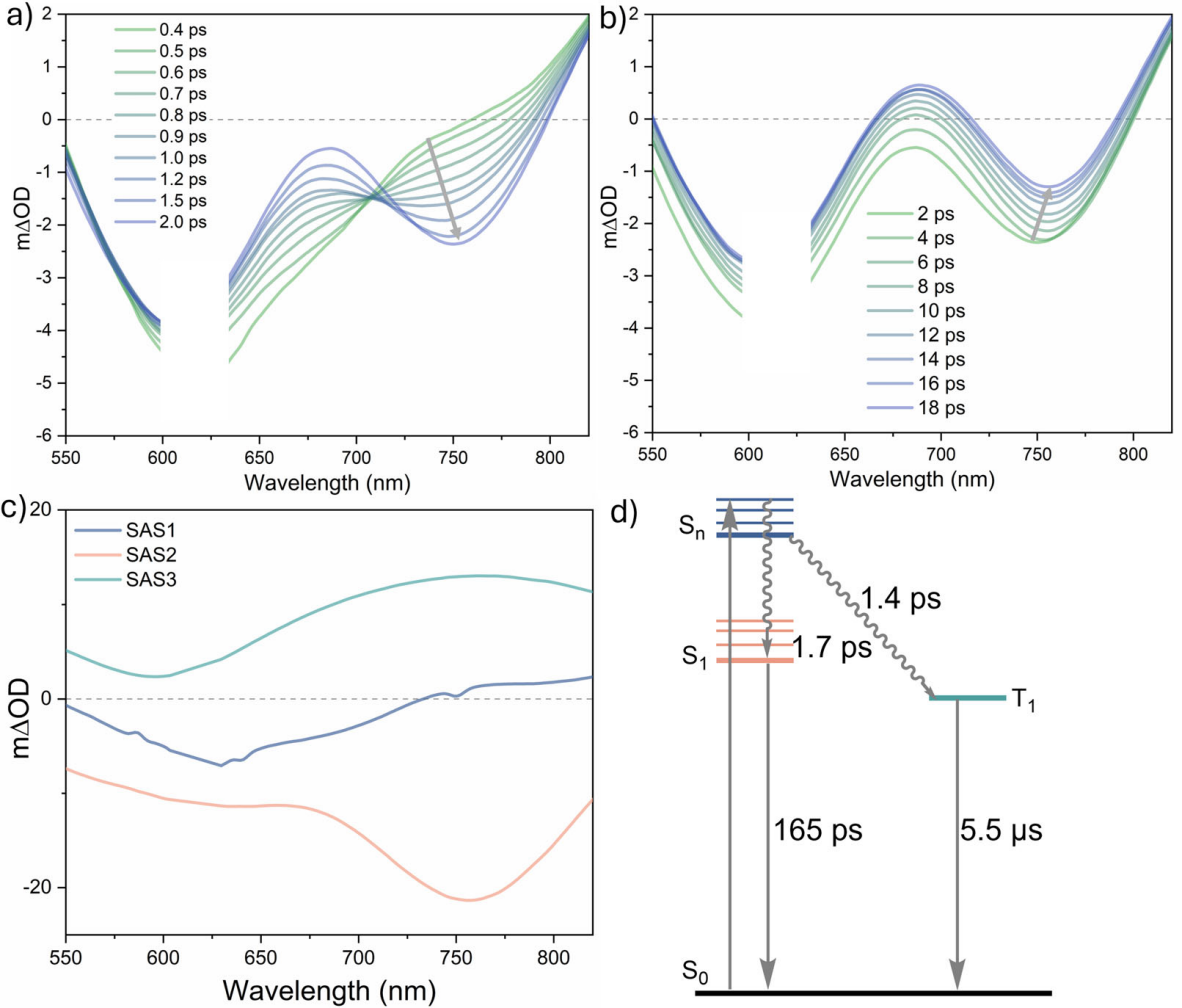
Femtosecond transient absorption spectra at different delay times for **[Hom‐Rh_2_]*^t^* ^Bu^** after excitation at 620 nm (OD_620 nm_  = 0.2); (a) at shorter delay times; (b) at longer delay times. Additional spectra at extended delay times (up to 890 ps) are given in the Supporting Information (Figure S94a). Arrows indicate changes in the spectral features over time. (c) Species‐associated spectra (SAS1, SAS2, and SAS3) of the global fit of the ultrafast transient absorption spectroscopy data of **[Hom‐Rh_2_]*^t^* ^Bu^**. (d) Schematic energy diagram of excited‐state relaxation pathways for **[Hom‐Rh_2_]*^t^* ^Bu^** obtained from the transient absorption studies. Measurements were performed in aerated CH_2_Cl_2_ at room temperature.

For **[Hom‐Rh_2_]*^t^* ^Bu^**, the initial TA spectrum (∼0.4 ps after excitation with pulses of ca. 0.2 ps duration) displays a GSB at ∼610 nm and an ESA above 750 nm (Figure 4a). Over the initial 2 ps interval, a fluorescence band emerges near 750 nm (Figure 4a) and relatively minor changes are observed between 2 and 18 ps (Figure 4b). An intense ESA spanning the visible region is observed at delay times beyond 100 ps (Figure S94a).

The data are described by a three‐state kinetic model (Figure 4d) in which relaxation from a higher‐lying excited‐state bifurcates into two distinct lower‐lying excited states, each characterized by a species‐associated spectrum (SAS) (Figure 4c). This bifurcation model aligns with the aforementioned finding that both a weakly fluorescent S_1_ (Figure S69a) and a phosphorescent T_1_ state (Figure 2c) are ultimately populated in **[Hom‐Rh_2_]*^t^* ^Bu^**. SAS1, assigned to a higher singlet excited‐state (S_n_) features only a GSB (blue trace in Figure 4c). The S_n_ state relaxes through two competitive processes, namely internal conversion (IC) to S_1_ and ISC to T_1_ with lifetimes of 1.7 and 1.4 ps, respectively (Figure 4d). Both of these time components include vibrational cooling to the relaxed S_1_ and T_1_ states; thus, the actual IC and ISC steps are likely faster, but cannot be resolved with the used experimental setup.

SAS2 (orange trace in Figure 4c), associated with the singlet S_1_ state, shows a prominent fluorescence feature at ∼750 nm (matching the weak fluorescence band, Figure S69a) and decays with a lifetime of 165 ps. SAS3 (green trace in Figure 4c) is characterized by a broad ESA across the visible region and decays beyond the timescale of the experiment. Its spectral feature is consistent with the nanosecond TA spectra (Figure 3a), supporting its assignment to the T_1_ state, which has a lifetime of ca. 5 µs determined from luminescence (inset of Figure 2c) and nanosecond TA experiments (inset of Figure 3a).

**FIGURE 5:**
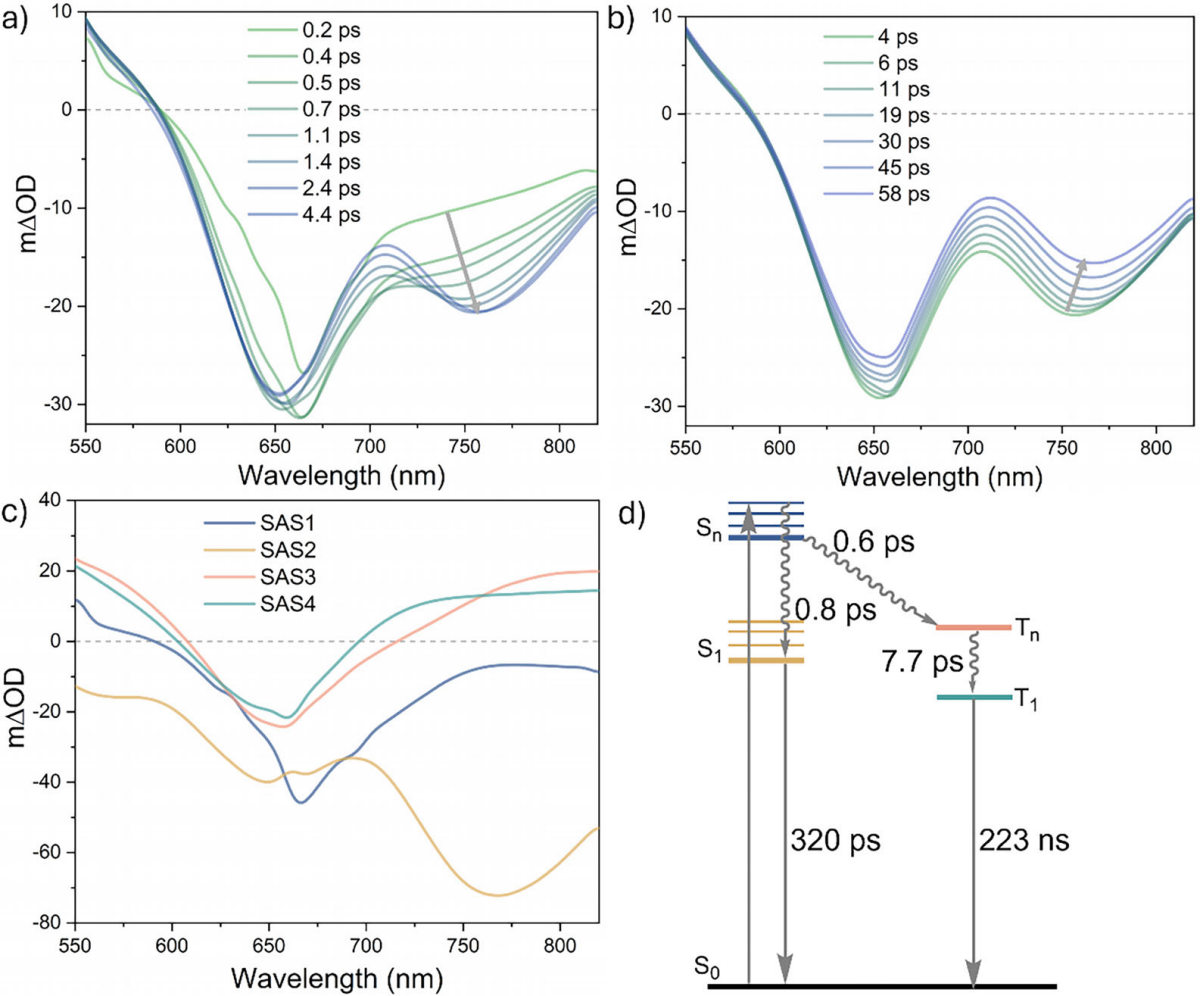
Femtosecond transient absorption spectra at different delay times for **[Het‐Rh_2_]*^t^* ^Bu^** after excitation at 660 nm (OD_660 nm_  =  0.2) (a) at shorter delay times; (b) at longer delay times. Additional spectra at extended delay times are given in the Supporting Information (Figure S95a). Arrows indicate changes in the spectral features over time. (c) Species‐associated spectra (SAS1, SAS2, SAS3, and SAS4) of the global fit of the ultrafast transient absorption spectroscopy data of **[Het‐Rh_2_]*^t^* ^Bu^**. (d) Schematic energy diagram of excited‐state relaxation pathways for **[Het‐Rh_2_]*^t^* ^Bu^** obtained from the transient absorption studies. Measurements were performed in aerated CH_2_Cl_2_ at room temperature.

For **[Het‐Rh_2_]*^t^* ^Bu^**, the initial TA spectrum (∼0.2 ps) exhibits a GSB at ∼660 nm (Figure 5a). Over the initial 4.4 ps, a prominent fluorescence band emerges near 760 nm. The spectral changes occurring between 4 ps and 58 ps are relatively minor (Figure 5b), but at delay times beyond 500 ps an ESA band becomes detectable beyond 700 nm (Figure S95a).

In contrast to the three‐state model (Figure 4d) for **[Hom‐Rh_2_]*^t^* ^Bu^**, the **[Het‐Rh_2_]*^t^* ^Bu^** complex requires a four‐state kinetic model (Figure 5d), each associated with an SAS (Figure 5c). As in the homoleptic analogue, SAS1 (blue trace in Figure 5c) is assigned to a higher singlet excited‐state (S_n_), which relaxes via two channels with lifetimes of 0.8 ps (IC to S_1_) and 0.6 ps (through another channel discussed below). SAS2, assigned to S_1_, features both a GSB and a fluorescence band (yellow trace in Figure 5c) and decays with a lifetime of 320 ps, consistent with the luminescence spectrum (Figure 2d) and the lifetime obtained from TCSPC measurements (*τ*_S1_  =  326 ps, inset of Figure 2d).

SAS3 (orange trace in Figure 5c) and SAS4 (green trace in Figure 5c) are spectrally similar, but they have very different decay times: 7.7 ps and > 5 ns, respectively. The latter is beyond the time window accessible in this fs‐TA experiment. Both SAS3 and SAS4 feature essentially the same ESA bands as the TA spectrum measured on the nanosecond timescale (Figure 3b): one above 700 nm and one below 600 nm. Consequently, the long‐lived SAS4 is attributed to the weakly phosphorescent T_1_ state, for which a lifetime of 223 ns was determined above (inset of Figure 3b).

Given the clear assignment of SAS4 to T_1_ and the spectral similarity between SAS4 and SAS3, the latter appears to be reasonably attributable to either a higher triplet excited‐state (T_n_) or a vibrationally excited‐state of T_1_. The electronic excitation of 4dσ*(Rh)→5pσ(Rh)/π*(isocyanide) leads to a contraction of the Rh^I^···Rh^I^ distance, because an electron is promoted from a metal‐metal antibonding dσ* to a metal‐metal bonding pσ orbital [96, 97, 98]. Such contractions were previously observed for Pt^II^···Pt^II^ dimers and Au^I^ oligomers, where excited‐state relaxation pathways proceed first through an intermediate state (involving nuclear reorganizations related to the metal‐metal distance) prior to reaching the emissive, fully relaxed T_1_ state [44, 45, 94, 99].

The kinetic models based on the fs‐TA experiments (Figures 4d and 5d) both support similar competition between IC (to populate S_1_) and ISC (ultimately leading to T_1_) from the initially excited S_n_ state in both **[Hom‐Rh_2_]*^t^* ^Bu^** and **[Het‐Rh_2_]*^t^* ^Bu^**. Therefore, we conclude that S_1_ and T_1_ are populated to similar extents in **[Hom‐Rh_2_]*^t^* ^Bu^** and **[Het‐Rh_2_]*^t^* ^Bu^**. The fluorescence quantum yields (*ϕ*_fl_) are both below 1% (Table 1). The main difference between these two compounds is that the phosphorescence quantum yield (*ϕ*_ph_) of **[Hom‐Rh_2_]*^t^* ^Bu^** is 40 times higher than that of **[Het‐Rh_2_]*^t^* ^Bu^** (Table 1). Given the factor of 25 difference in T_1_ lifetimes (Table 1), the difference in *ϕ*_ph_ can be traced back to less efficient nonradiative relaxation from T_1_ to S_0_ in **[Hom‐Rh_2_]*^t^* ^Bu^** than in **[Het‐Rh_2_]*^t^* ^Bu^**. This can be partially explained by the fact that the T_1_ energy of **[Hom‐Rh_2_]*^t^* ^Bu^** is approximately 1000 cm^−1^ higher than that of **[Het‐Rh_2_]*^t^* ^Bu^** (based on the phosphorescence band maxima in Figure 2c,d), which favors nonradiative relaxation from **[Het‐Rh_2_]*^t^* ^Bu^** according to the energy gap law [100]. Furthermore, the different molecular structures with partially staggered [Rh(CNR)_4_] units in **[Hom‐Rh_2_]*^t^* ^Bu^** (Figure 1a) and eclipsed [Rh(CNR)_2_(P)_2_] units in **[Het‐Rh_2_]*^t^* ^Bu^** (Figure 1b) could undergo different types and extents of excited‐state distortions that affect nonradiative relaxation.

**TABLE 1:** Key photophysical parameters of **[Hom‐Rh_2_]^*t*Bu^**, **[Het‐Rh_2_]^*t*Bu^**, and **[Hom‐Rh_2_]^Mes^** measured in deoxygenated CH_2_Cl_2_ at room temperature, unless otherwise stated.

Complex	[Hom‐Rh_2_]*^t^* ^Bu^	[Het‐Rh_2_]*^t^* ^Bu^	[Hom‐Rh_2_]^Mes^
*τ*_S1_/ns	0.165^a^	0.326^b^	n.d.^f^
*τ*_T1_/µs^c^	5.5	0.223	4.0
*ϕ*_fl_ ^d^	< 0.3%^e^	0.7%	< 0.2%^e^
*ϕ*_ph_ ^d^	8.0%	0.2%	5.5%

^a^ The value was obtained from TA measurements (*λ*_exc_ = 620 nm and *λ*_obs_ = 750 nm) in aerated CH_2_Cl_2_ at room temperature.

^b^ The value was obtained from TCSPC measurements (*λ*_exc_ = 635 nm and *λ*_obs_ = 725 nm).

^c^ The values were obtained from transient UV‐Vis absorption measurements (for **[Hom‐Rh_2_]*^t^* ^Bu^** and **[Hom‐Rh_2_]^Mes^** *λ*_exc_ = 625 nm and *λ*_obs_ = 790 nm, for **[Het‐Rh_2_]*^t^* ^Bu^** *λ*_exc_ = 650 nm and *λ*_obs_ = 450 nm).

^d^ The luminescence quantum yields were measured using [Os(bpy)_3_](PF_6_)_2_ as a reference in deoxygenated CH_3_CN (*ϕ*_MeCN_ = 0.5 %) [101].

^e^ The upper limit of the fluorescence quantum yield (*ϕ*_fl_) at 745 nm was estimated with reference to the phosphorescence quantum yield (*ϕ*_ph_) by comparing the relative intensities of fluorescence and phosphorescence bands.

^f^ n.d. = not determined.

**[Hom‐Rh_2_]*^t^* ^Bu^** stands out from previously investigated homoleptic paddlewheel Rh^I^ dimer complexes with bridging alkyl isocyanide due to its phosphorescence properties [68, 71]. Aryl isocyanide ligands appear to lead to a Rh_2_ core, in which nonradiative relaxation from the T_1_ state is decelerated [67]. In particular, the Rh^I^ dimer core appears to be more rigid with our aryl isocyanide ligands due to the partially staggered conformation between the two involved square‐planar coordination units, which are more mutually interlocked into one another than in the case of Rh^I^ dimers with alkyl isocyanide ligands, where eclipsed conformations between individual [Rh(CNR)_4_]^+^ units are more common. This partially staggered geometry may further suppress nonradiative decay by limiting molecular distortions during excited‐state bond shortening [96, 97], whereas in the eclipsed analogue, the closer approach of the square‐planar units is likely to increase interunit repulsion and consequently additional molecular distortions.

### 2.4 Tuning Metal‐Metal Distances and Triplet Lifetimes via Ligand Periphery Modifications

**FIGURE 6:**
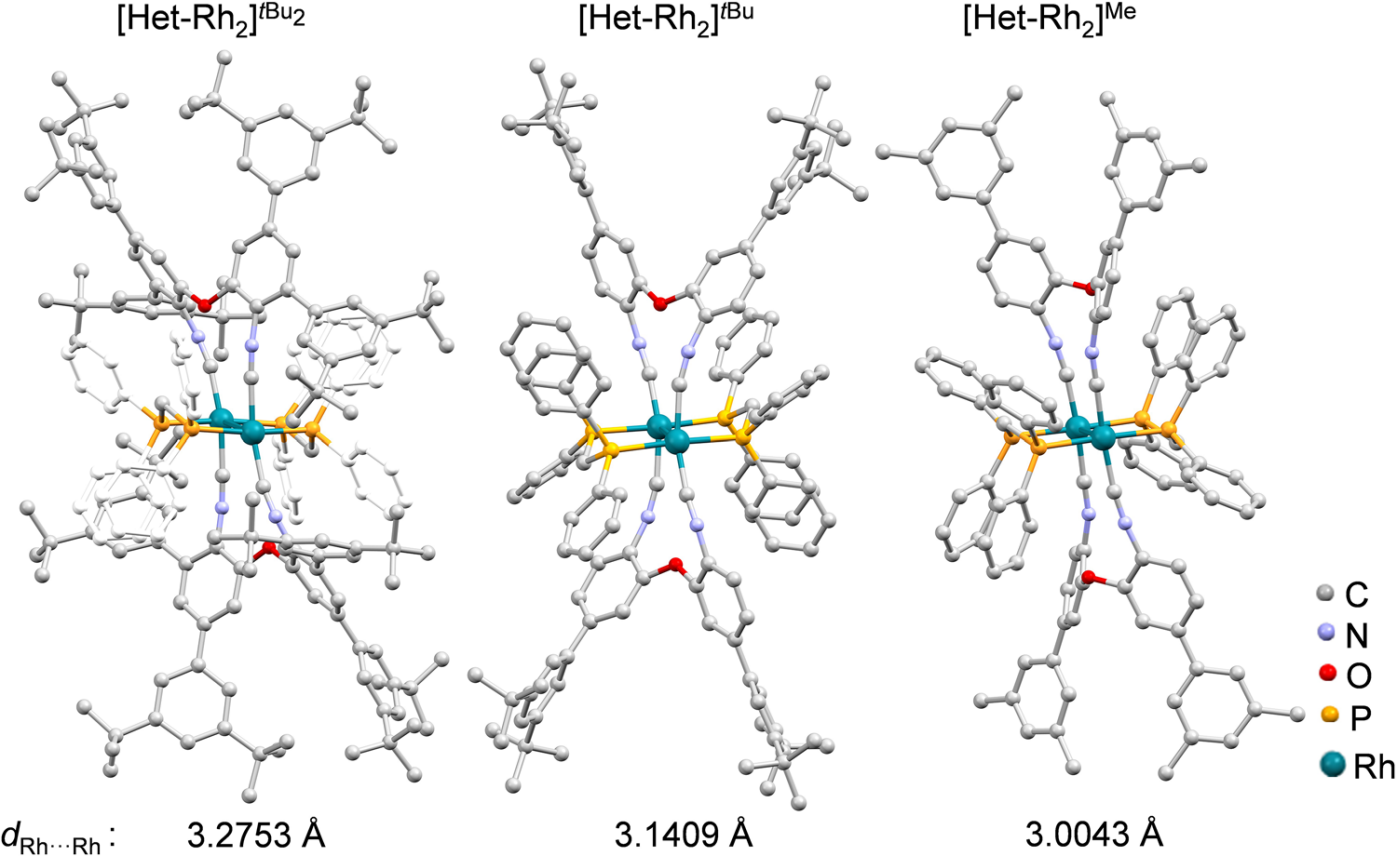
Molecular structures of a series of heteroleptic Rh^I^ complexes (with structural modification in the ligand periphery) in the solid state, along with their Rh···Rh distances. Counter‐anions, hydrogen atoms and non‐coordinating solvent molecules are omitted for clarity. The phenyl groups attached to the phosphine moiety of **[Het‐Rh_2_]*^t^* ^Bu2^** are colored white for visual clarity. Structural parameters are given in the Supporting Information (Figures S63–S65, Tables S2 and S3).

In the second part of this work, our focus was on the heteroleptic molecular design. We sought to control the fluorescence‐to‐phosphorescence ratio and triplet lifetimes by varying the metal‐metal distance. To this end, we synthesized three different heteroleptic complexes (**[Het‐Rh_2_]*^t^* ^Bu2^**, **[Het‐Rh_2_]*^t^* ^Bu^** and **[Het‐Rh_2_]^Me^**) through peripheral ligand modifications designed to modulate steric effects. The complexes were characterized using standard analytical techniques, including SCXRD (Figure 6) and key photophysical parameters, along with Rh^I^···Rh^I^ distances, which are tabulated in Table 2.

**TABLE 2:** Key photophysical parameters and Rh^I^···Rh^I^ distances of the heteroleptic Rh^I^ dimers, with photophysical measurements carried out in deoxygenated CH_2_Cl_2_ at room temperature.

Complex	[Het‐Rh_2_]*^t^* ^Bu2^	[Het‐Rh_2_]*^t^* ^Bu^	[Het‐Rh_2_]^Me^
*τ*_S1_/ns^a^	< 0.061^d^	0.326	0.331
*τ*_T1_/ns^b^	N/A^e^	223	477
*ϕ*_fl_ ^c^	0.2%	0.7%	0.7%
*ϕ*_ph_ ^c^	N/A^e^	0.2%	0.3%
*ϕ*_ph_/*ϕ*_fl_	N/A	0.3	0.4
*d*_Rh···Rh_/Å	3.2753(5)	3.1409(2)	3.0043(10)

^a^ The values were obtained from TCSPC measurements (*λ*_exc_ = 635 nm and *λ*_obs_ = 725 nm).

^b^ The values were obtained from transient UV‐Vis absorption measurements (*λ*_exc_ = 650 nm and *λ*_obs_ = 450 nm).

^c^ The quantum yields were measured using [Os(bpy)_3_](PF_6_)_2_ as a reference in deoxygenated CH_3_CN (*ϕ*_MeCN_  = 0.5 %) [101].

^d^ Upper limit imposed by the instrumental limitations.

^e^ No transient absorption nor luminescence for T_1_ observable in this case.

All three complexes adopt an eclipsed conformation at the Rh_2_ core, thereby minimizing conformational variability at the metal center (Figure S66), which is known to influence the photophysical properties [67]. By maintaining a consistent conformation at the Rh_2_ core, the peripheral steric modifications enabled the isolation of Rh^I^ complexes with different metal‐metal distances. Although several Rh^I^ complexes exhibiting a range of Rh^I^···Rh^I^ distances have been reported [65, 67], the synthetic approach to control the Rh^I^···Rh^I^ distances, as shown here, is unusually simple.

The ligand **L*^t^* ^Bu2^** was synthesized by introducing 3,5‐di‐*tert*‐butylphenyl substituents at both the ortho‐ and para‐positions to the isocyanide group. Based on examples from Pt^II^ and Pd^II^ dimers [50, 102], ortho‐substitution was anticipated to enforce a shorter Rh^I^···Rh^I^ distance relative to **[Het‐Rh_2_]*^t^* ^Bu^**. Instead, **[Het‐Rh_2_]*^t^* ^Bu2^** displays an elongated metal‐metal distance of 3.2753(5) Å, compared to 3.1408(2) Å in **[Het‐Rh_2_]*^t^* ^Bu^** (Figure 6). In contrast, replacing the *t*‐butyl groups with methyl groups (**[Het‐Rh_2_]^Me^**) at the periphery leads to contraction of the Rh^I^···Rh^I^ distance of 3.0043(10) Å (Figure 6), which is shorter than that reported for [Rh(dmb)_2_(dppm)_2_]^2+^ (3.037 Å) [71].

The observed steric dependence differs from trends commonly reported for Pt^II^ dimers [38, 102], in which increased steric bulk is associated with contraction of the Pt^II^···Pt^II^ distance. Across the present series, the Rh^I^···Rh^I^ separation varies over 0.2 Å (Figure 6), enabling direct correlation of metal‐metal distance with photophysical properties.

**FIGURE 7:**
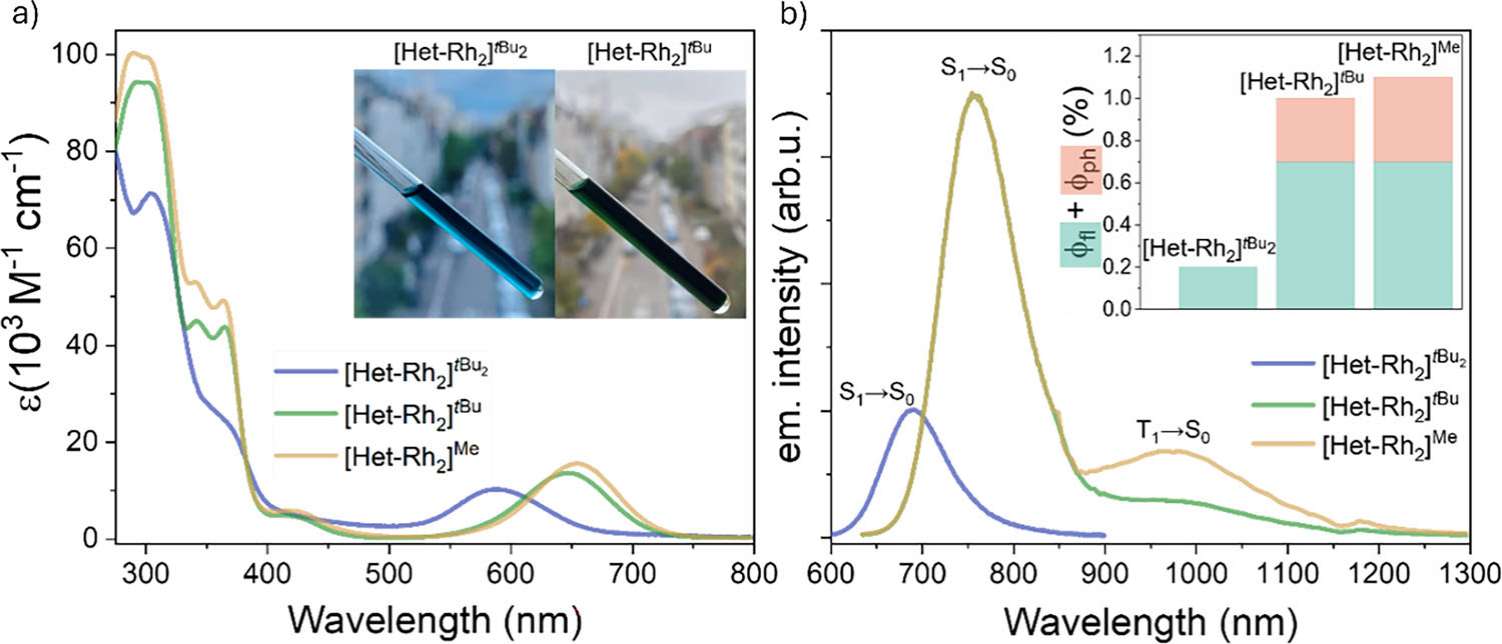
Steady‐state photophysical characterization of a series of heteroleptic Rh^I^ dimers **([Het‐Rh_2_]*^t^* ^Bu2^**, **[Het‐Rh_2_]*^t^* ^Bu^**, **[Het‐Rh_2_]^Me^**). (a) UV‐Vis absorption spectra of the heteroleptic Rh^I^ dimers. Inset: Pictures of NMR tubes containing a solution of **[Het‐Rh_2_]*^t^* ^Bu2^** and **[Het‐Rh_2_]*^t^* ^Bu^**. (b) Photoluminescence spectra of the heteroleptic Rh^I^ dimers, scaled relative to one another according to their respective *ϕ*_fl_. Inset: Plot depicting the sum of *ϕ*_fl_ and *ϕ*_ph_ of heteroleptic complexes and their ratios. All measurements were performed in deoxygenated CH_2_Cl_2_ at room temperature.

Consistent with the structural variation, the complexes exhibit different photophysical properties (Figure 7). The 4dσ*(Rh)→5pσ(Rh)/π*(isocyanide) absorption band of **[Het‐Rh_2_]*^t^* ^Bu2^** appears between 530 and 670 nm (*ε*_585 nm_  =  9800 M^−1^ cm^−1^) in CH_2_Cl_2_, and is the most blue‐shifted in energy within the series, followed by **[Het‐Rh_2_]*^t^* ^Bu^** (*ε*_648 nm_  =  13000 M^−1^ cm^−1^) and **[Het‐Rh_2_]^Me^** (*ε*_648 nm_  =  15000 M^−1^ cm^−1^), although **[Het‐Rh_2_]*^t^* ^Bu^** and **[Het‐Rh_2_]^Me^** differ marginally. The energy of the absorption band correlates with the Rh^I^···Rh^I^ distance, such that increasing metal‐metal distance leads to higher energy of the [4dσ* (Rh)→5pσ (Rh)/π*(isocyanide)] transition [71].

**[Het‐Rh_2_]*^t^* ^Bu2^** (featuring the longest Rh^I^···Rh^I^ distance) exhibits a fluorescence band at ∼690 nm with a *ϕ*_fl_ of 0.2 % (Figure 7b). The absence of phosphorescence or a nanosecond TA signal indicates that the triplet manifold is not populated or is very short‐lived. It seems possible that the longer Rh^I^···Rh^I^ distance leads to less efficient intersystem crossing, since excited‐state energetics and spin‐orbit coupling strongly depend on the distance and relative orientation of the d_z_^2^ orbitals in previously investigated d^8^‐d^8^ dimers [44, 103]. While this rationale appears applicable to this series of heteroleptic complexes, it does not account for the observation that the homoleptic complexes **[Hom‐Rh_2_]*^t^* ^Bu^** and **[Hom‐Rh_2_]^Mes^** exhibit Rh···Rh distances and excited state energetics similar to those of **[Het‐Rh_2_]*^t^* ^Bu2^**, yet the former display phosphorescence, whereas the latter does not. Clearly, a more nuanced description is required to fully rationalize intersystem crossing across a structurally diverse set of Rh_2_ dimers, one that goes beyond what is presently attainable.

The photophysical properties of **[Het‐Rh_2_]*^t^* ^Bu^** (featuring a Rh···Rh distance in between those of the two other heteroleptic complexes from Figure 6) were discussed in detail in the first part of this work. In brief, to provide context for its properties relative to those of its heteroleptic congeners investigated in this section, the complex shows a dominant fluorescence band centered at ∼750 nm (red‐shifted by 0.25 eV relative to **[Het‐Rh_2_]*^t^* ^Bu2^**) along with a shoulder due to phosphorescence at ∼950 nm, with lifetimes of 326 ps and 223 ns, respectively (Table 2). The complex **[Het‐Rh_2_]^Me^** with the shortest metal‐metal distance, displays a similar fluorescence band centered at ∼750 nm (*ϕ*_fl_  =  0.7 %,  *τ*_s1_ = 331 ps Figure S75), and a more pronounced phosphorescence band in the NIR‐II region at ∼975 nm (*ϕ*_ph_  =  0.3 %) (Figure 7b). This phosphorescence band is slightly red‐shifted (∼300 cm^−1^) and exhibits a longer lifetime of 477 ns (obtained from ns TA measurements, Figure S92b, compared to **[Het‐Rh_2_]*^t^* ^Bu^** (223 ns). Based on the fluorescence and phosphorescence spectra of **[Het‐Rh_2_]*^t^* ^Bu^** and **[Het‐Rh_2_]^Me^** (Figures S76 and S77), we determine energy differences of over 0.3 eV between the S_1_ and T_1_ excited states. This makes reverse ISC unlikely in these compounds, which is consistent with the observation of mono‐exponential fluorescence decays in the picosecond range; in other words, there is no evidence of thermally activated delayed fluorescence (TADF) in these compounds.

Across this series, modulation of the Rh^I^···Rh^I^ distance primarily influences the phosphorescence‐to‐fluorescence ratio (inset of Figure 7b) and the triplet excited‐state lifetimes (Table 2). However, changing peripheral substituents in the homoleptic complexes (**[Hom‐Rh_2_]*^t^* ^Bu^** and **[Hom‐Rh_2_]^Mes^**) does not influence the phosphorescence‐to‐fluorescence ratios (Table 1 and Figure S87). This behavior contrasts with previously reported Pt^II^ dimers, in which changes in the metal‐metal distance led to pronounced shifts in emission energy and alterations in the nature of the charge‐transfer excited‐state [38, 42]. In our case, the increase in phosphorescence quantum yield and T_1_ lifetime with a decreased metal‐metal distance (among the heteroleptic series) appears to be mostly due to slower nonradiative excited‐state relaxation. This is evident when comparing the *ϕ*_ph_ and  τ_T1_ values of **[Het‐Rh_2_]*^t^* ^Bu^** and **[Het‐Rh_2_]^Me^** in Table 2. Similarly, fluorescence benefits from shortened metal‐metal distances, as seen in the comparison of *ϕ*_fl_ and  τ_S1_ values of **[Het‐Rh_2_]*^t^* ^Bu2^** and **[Het‐Rh_2_]*^t^* ^Bu^** (inset in Figure 7b and Table 2). Increased rigidity of the Rh_2_ core, brought about by shortened metal‐metal distances, seems to plausibly help restrict molecular distortions and vibrations that dissipate energy in a nonradiative fashion in both the S_1_ and T_1_ excited states. When the temperature was lowered from 290 K to 180 K, the phosphorescence‐to‐fluorescence ratio increased ninefold for **[Het‐Rh_2_]*^t^* ^Bu^** (Figure S78) and fivefold for **[Het‐Rh_2_]^Me^** (Figure S79). These results support the hypothesis that structural rigidification optimizes phosphorescence properties.

## 3 Conclusions

Photoactive d^8^‐d^8^ metal dimers have been known for nearly 50 years, and while early work investigated Rh^I^ complexes with alkyl isocyanide bridging ligands [64, 67, 68, 69, 71, 104], aryl isocyanide variants have been largely overlooked until now. Using such ligands in homo‐ and heteroleptic Rh^I^ dimer complexes provides the following new insights:

1. The use of aryl isocyanide ligands in place of alkyl isocyanides enhances phosphorescence in both homoleptic lantern‐type complexes and their heteroleptic analogues. The aryl isocyanides used in this work lead to a homoleptic complex with a partially staggered conformation of individual [Rh(CNR)_4_]^+^ units, making the Rh_2_ core more compact and rigid, in contrast to previously reported alkyl isocyanide Rh^I^ dimers, which feature an eclipsed geometry and a more structurally flexible Rh_2_ core [68]. It seems plausible that this enhanced rigidity suppresses nonradiative relaxation pathways, resulting in enhanced phosphorescence quantum yields and T_1_ lifetimes.
2. Directly comparing a homoleptic complex and a heteroleptic complex (Figure 1a,b) that use the same aryl isocyanide ligand (and, in the case of the heteroleptic complex, a di‐phosphine bridging ligand) further emphasizes the importance of controlling the structure of the Rh_2_ core. In the homoleptic complex, partially staggered [Rh(CNR)_4_]^+^ units lead to NIR‐II phosphorescence. In contrast, eclipsed [Rh(CNR)_4_]^+^ units in the heteroleptic compound lead to NIR‐I fluorescence and a weak NIR‐II phosphorescence, observed for the first time for heteroleptic Rh^I^ complexes (Figure 2c,d).
3. Furthermore, rigidification of the Rh_2_ core appears achievable by modulating the peripheral substituents of the di‐isocyanide bridging ligands in the secondary coordination sphere of heteroleptic complexes (Figure 6, Table 2). In this case, it appears that rigidification is primarily caused by a decrease in the distance between metals. This leads to less nonradiative relaxation, resulting in improved S_1_ and T_1_ excited state properties, including higher fluorescence and phosphorescence quantum yields and longer lifetimes.
4. Ultrafast spectroscopy hints at distinct ISC pathways between homoleptic and heteroleptic complexes. However, this appears to affect the branching of the initially excited higher singlet excited states into the photoactive S_1_ and T_1_ excited states only to a minor extent, but could eventually become a mechanism to control ISC.

These combined findings are relevant to improving the photophysical properties and the photochemical applicability of d^8^‐d^8^ dimers. They provide insight into tuning the fluorescence‐to‐phosphorescence ratios, which could be relevant for (bio)imaging, because long‐lived phosphorescence can be readily distinguished from background fluorescence in time‐gated luminescence measurements. Moreover, the fact that a substantial portion of the phosphorescence from these dimers lies within the NIR‐II spectral region is noteworthy for biological applications, owing to the much greater tissue penetration and transparency in this range compared to other spectral regions.

Future work may focus on gaining further control over the S_1_ and T_1_ populations by controlling the ISC from the initially populated higher singlet excited state. Our ultrafast studies suggest this could be achieved by controlling the primary coordination sphere, as ISC pathways appear different in homo‐ and heteroleptic compounds (Figures 4d and 5d). This would be relevant to photocatalysis, in which T_1_ excited states demonstrate predominant triplet‐triplet energy transfer reactivity, while S_1_ states show predominant electron transfer reactivity [105]. Lastly, extending these principles and insights to first‐row transition metal complexes represents an attractive and feasible direction for future work [2].

## Conflicts of Interest

The authors declare no conflicts of interest.

## Supporting information




**Supporting File 1**: anie72080‐sup‐0001‐SuppMat.docx.

**Supporting File 2**: anie72080‐sup‐0002‐Data.zip.

## Data Availability

The data that supports the findings of this study are available in the supplementary material of this article.

## References

[1] C. Förster and K. Heinze , “Photophysics and Photochemistry With Earth‐Abundant Metals—Fundamentals and Concepts,” Chemical Society Reviews 49 (2020): 1057–1070, 10.1039/C9CS00573K. 32025671

[2] G. Morselli , C. Reber , and O. S. Wenger , “Molecular Design Principles for Photoactive Transition Metal Complexes: A Guide for ‘Photo‐Motivated’ Chemists,” Journal of the American Chemical Society 147 (2025): 11608–11624, 10.1021/jacs.5c02096. 40147007 PMC11987026

[3] C. Bruschi , X. Gui , P. Rauthe , et al., “Dual Role of a Novel Heteroleptic Cu(I) Complex in Visible‐Light‐Driven CO_2_ Reduction,” Chemistry: A European Journal 30 (2024): e202400765, 10.1002/chem.202400765. 38742808

[4] D. Tungulin , J. Leier , A. B. Carter , et al., “Chasing BODIPY: Enhancement of Luminescence in Homoleptic Bis(dipyrrinato) Zn^II^ Complexes Utilizing Symmetric and Unsymmetrical Dipyrrins,” Chemistry: A European Journal 25 (2019): 3816–3827, 10.1002/chem.201806330. 30687972

[5] Z.‐L. Gong , H.‐J. Zhang , Y. Cheng , et al., “Recent Progress on Photoactive Nonprecious Transition‐Metal Complexes,” Science China Chemistry 68 (2025): 46–95, 10.1007/s11426-024-2345-0.

[6] T. Huang , P. Du , and Y.‐M. Lin , “Recent Advances in Photoactive First‐Row Transition Metal Complexes for Organic Synthesis,” Chinese Journal of Chemistry 43 (2025): 2566–2587, 10.1002/cjoc.70135.

[7] J. K. McCusker , “Electronic Structure in the Transition Metal Block and Its Implications for Light Harvesting,” Science 363 (2019): 484–488, 10.1126/science.aav9104. 30705184

[8] B. M. Hockin , C. Li , N. Robertson , and E. Zysman‐Colman , “Photoredox Catalysts Based on Earth‐Abundant Metal Complexes,” Catalysis Science & Technology 9 (2019): 889–915, 10.1039/C8CY02336K.

[9] J. Leier , P. Rauthe , R. Tabone , C. Bizzarri , and A.‐N. Unterreiner , “Excited state Dynamics of Homoleptic Zn(II)Dipyrrin Complexes and Their Application in Photocatalysis,” New Journal of Chemistry 48 (2024): 13261–13269, 10.1039/D4NJ02527J.

[10] R. Maity , B. S. Birenheide , F. Breher , and B. Sarkar , “Cooperative Effects in Multimetallic Complexes Applied in Catalysis,” ChemcCatChem 13 (2021): 2337–2370, 10.1002/cctc.202001951.

[11] A. Pannwitz and O. S. Wenger , “Proton‐Coupled Multi‐Electron Transfer and Its Relevance for Artificial Photosynthesis and Photoredox Catalysis,” Chemical Communications 55 (2019): 4004–4014, 10.1039/C9CC00821G. 30810148

[12] L. Hammarström , “Accumulative Charge Separation for Solar Fuels Production: Coupling Light‐Induced Single Electron Transfer to Multielectron Catalysis,” Accounts of Chemical Research 48 (2015): 840–850, 10.1021/ar500386x. 25675365

[13] M. Brändlin , B. Pfund , and O. S. Wenger , “Photoinduced Double Charge Accumulation in a Molecular Compound,” Nature Chemistry 17 (2025): 1777–1784, 10.1038/s41557-025-01912-x. PMC12580319 40855362

[14] A. König , R. Naumann , C. Förster , J. Klett , and K. Heinze , “A Near‐Infrared‐II Luminescent and Photoactive Vanadium(II) Complex With a 760 ns Excited State Lifetime,” Journal of the American Chemical Society 147 (2025): 20833–20842, 10.1021/jacs.5c04471. 40462271 PMC12186473

[15] J. M. Sledesky , J. H. Zimmerman , H. C. London , et al., “Xylylethynyl Titanocene With a Microsecond Emission Lifetime Photosensitizes Singlet‐Oxygen Formation and Photon Upconversion,” Inorganic Chemistry 64 (2025): 14977–14988, 10.1021/acs.inorgchem.5c01773. 40673496 PMC12308804

[16] V. Q. Dang , C. Jiang , and T. S. Teets , “Enhanced Blue Phosphorescence in Platinum Acetylide Complexes via a Secondary Heavy Metal and Anion‐Controlled Aggregation,” Chemical Science 16 (2025): 7302–7310, 10.1039/D5SC00172B. 40144504 PMC11934150

[17] G. Morselli , T. H. Eggenweiler , M. Villa , A. Prescimone , and O. S. Wenger , “Pushing the Thermodynamic and Kinetic Limits of Near‐Infrared Emissive Cr^III^ Complexes in Photocatalysis,” Journal of the American Chemical Society 147 (2025): 28226–28240, 10.1021/jacs.5c08541. 40720722 PMC12333365

[18] F. Glaser and O. S. Wenger , “Red Light‐Based Dual Photoredox Strategy Resembling the Z‐Scheme of Natural Photosynthesis,” JACS Au 2 (2022): 1488–1503, 10.1021/jacsau.2c00265. 35783177 PMC9241018

[19] P. Neidinger , J. Davis , D. Voll , et al., “Near Infrared Light Induced Radical Polymerization in Water,” Angewandte Chemie, International Edition 61 (2022): e202209177, 10.1002/anie.202209177. 35945906 PMC9826492

[20] P. Neidinger , D. Voll , S. L. Walden , A.‐N. Unterreiner , and C. Barner‐Kowollik , “Two Photon Induced Pulsed Laser Polymerization With Near Infrared Light,” ACS Macro Letters 12 (2023): 308–313, 10.1021/acsmacrolett.3c00063. 36787646

[21] C. Li , Y. Pang , Y. Xu , et al., “Near‐Infrared Metal Agents Assisting Precision Medicine: From Strategic Design to Bioimaging and Therapeutic Applications,” Chemical Society Reviews 52 (2023): 4392–4442, 10.1039/D3CS00227F. 37334831

[22] R. Tabone , D. Feser , E. D. Lemma , U. Schepers , and C. Bizzarri , “Intriguing Heteroleptic Zn^II^ Bis(Dipyrrinato) Emitters in the Far‐Red Region With Large Pseudo‐Stokes Shift for Bioimaging,” Frontiers in Chemistry 9 (2021): 1–9, 10.3389/fchem.2021.754420. PMC8495118 34631672

[23] H. Janeková , M. Russo , U. Ziegler , and P. Štacko , “Photouncaging of Carboxylic Acids From Cyanine Dyes With Near‐Infrared Light,” Angewandte Chemie, International Edition 61 (2022): e202204391, 10.1002/anie.202204391. 35578980 PMC9542589

[24] K. M. Kuznetsov , K. Cariou , and G. Gasser , “Two in One: Merging Photoactivated Chemotherapy and Photodynamic Therapy to Fight Cancer,” Chemical Science 15 (2024): 17760–17780, 10.1039/D4SC04608K. 39464604 PMC11499979

[25] S. Bonnet , “Why Develop Photoactivated Chemotherapy?” Dalton Transactions 47 (2018): 10330–10343, 10.1039/C8DT01585F. 29978870

[26] S. Callaghan and M. O. Senge , “The Good, the Bad, and the Ugly–Controlling Singlet Oxygen Through Design of Photosensitizers and Delivery Systems for Photodynamic Therapy,” Photochemical & Photobiological Sciences 17 (2018): 1490–1514, 10.1039/c8pp00008e. 29569665

[27] Y. Zhang , Y. Wang , J. Song , et al., “Near‐Infrared Emitting Materials via Harvesting Triplet Excitons: Molecular Design, Properties, and Application in Organic Light Emitting Diodes,” Advanced Optical Materials 6 (2018): 1800466, 10.1002/adom.201800466.

[28] H. C. London , D. Y. Pritchett , J. A. Pienkos , et al., “Photochemistry and Photophysics of Charge‐Transfer Excited States in Emissive *d* ^10^/*d* ^0^ Heterobimetallic Titanocene Tweezer Complexes,” Inorganic Chemistry 61 (2022): 10986–10998, 10.1021/acs.inorgchem.2c01746. 35786924

[29] R. Englman and J. Jortner , “The Energy Gap Law for Radiationless Transitions in Large Molecules,” Molecular Physics 18 (1970): 145–164, 10.1080/00268977000100171.

[30] J. V. Caspar and T. J. Meyer , “Application of the Energy Gap Law to Nonradiative, Excited‐State Decay,” Journal of Physical Chemistry 87 (1983): 952–957, 10.1021/j100229a010.

[31] P. Kim , M. S. Kelley , A. Chakraborty , et al., “Coherent Vibrational Wavepacket Dynamics in Platinum(II) Dimers and Their Implications,” Journal of Physical Chemistry C 122 (2018): 14195–14204, 10.1021/acs.jpcc.8b01636.

[32] Y. Wada , T. Maruchi , R. Ishii , and Y. Sunada , “Visible Light Responsive Dinuclear Zinc Complex Consisting of Proximally Arranged Two *d* ^10^ ‐Zinc Centers,” Angewandte Chemie International Edition 62 (2023): e202310571, 10.1002/anie.202310571. 37753736

[33] V. R. Naina , S. Gillhuber , C. Ritschel , et al., “Dye Induced Luminescence Properties of Gold(I) Complexes With near Unity Quantum Efficiency,” Angewandte Chemie International Edition 64 (2025): e202414517, 10.1002/anie.202414517. 39183175 PMC11701351

[34] V. R. Naina , A. K. Singh , Subham , J. Krämer , M. Iqbal , and P. W. Roesky , “Synthesis of Luminescent Coumarin‐Substituted Phosphinoamide‐Bridged Polynuclear Gold(I) Metallacycles and Reactivity Studies,” Inorganic Chemistry Frontiers 11 (2024): 6079–6088, 10.1039/D4QI01747A.

[35] M. Kato , H. Ito , M. Hasegawa , and K. Ishii , “Soft Crystals: Flexible Response Systems With High Structural Order,” Chemistry: A European Journal 25 (2019): 5105–5112, 10.1002/chem.201805641. 30653768 PMC6593753

[36] Z. Li , H. T. Chifotides , and K. R. Dunbar , “Unprecedented Partial Paddlewheel Dirhodium Methyl Isocyanide Compounds With Unusual Structural and Electronic Properties: A Comprehensive Experimental and Theoretical Study,” Chemical Science 4 (2013): 4470–4485, 10.1039/c3sc51641e.

[37] T. Theiss , S. Buss , I. Maisuls , et al., “Room‐Temperature Phosphorescence From Pd(II) and Pt(II) Complexes as Supramolecular Luminophores: The Role of Self‐Assembly, Metal–Metal Interactions, Spin–Orbit Coupling, and Ligand‐Field Splitting,” Journal of the American Chemical Society 145 (2023): 3937–3951, 10.1021/jacs.2c09775. 36780431

[38] S. E. Brown‐Xu , M. S. J. Kelley , K. A. Fransted , et al., “Tunable Excited‐State Properties and Dynamics as a Function of Pt–Pt Distance in Pyrazolate‐Bridged Pt(II) Dimers,” Journal of Physical Chemistry A 120 (2016): 543–550, 10.1021/acs.jpca.5b11233. 26759897

[39] A. P. Zipp , “The Behavior of the Tetra‐υ‐Pyrophosphito‐Diplatinum(II) Ion Pt_2_(P_2_O_5_H_2_)^4–^ _4_ and Related Species,” Coordination Chemistry Reviews 84 (1988): 47–83, 10.1016/0010-8545(88)80031-6.

[40] D. M. Roundhill , H. B. Gray , and C. M. Che , “Pyrophosphito‐Bridged Diplatinum Chemistry,” Accounts of Chemical Research 22 (1989): 55–61, 10.1021/ar00158a002.

[41] A. Chakraborty , J. C. Deaton , A. Haefele , and F. N. Castellano , “Charge‐Transfer and Ligand‐Localized Photophysics in Luminescent Cyclometalated Pyrazolate‐Bridged Dinuclear Platinum(II) Complexes,” Organometallics 32 (2013): 3819–3829, 10.1021/om400276v.

[42] A. Chakraborty , J. E. Yarnell , R. D. Sommer , S. Roy , and F. N. Castellano , “Excited‐State Processes of Cyclometalated Platinum(II) Charge‐Transfer Dimers Bridged by Hydroxypyridines,” Inorganic Chemistry 57 (2018): 1298–1310, 10.1021/acs.inorgchem.7b02736. 29336558

[43] C. A. Strassert , C.‐H. Chien , M. D. Galvez Lopez , et al., “Switching on Luminescence by the Self‐Assembly of a Platinum(II) Complex Into Gelating Nanofibers and Electroluminescent Films,” Angewandte Chemie International Edition 50 (2011): 946–950, 10.1002/anie.201003818. 21246698

[44] S. R. Rather , N. P. Weingartz , S. Kromer , F. N. Castellano , and L. X. Chen , “Spin–Vibronic Coherence Drives Singlet–Triplet Conversion,” Nature 620 (2023): 776–781, 10.1038/s41586-023-06233-y. 37468632

[45] P. Kim , A. J. S. Valentine , S. Roy , et al., “Ultrafast Excited‐State Dynamics of Photoluminescent Pt(II) Dimers Probed by a Coherent Vibrational Wavepacket,” Journal of Physical Chemistry Letters 12 (2021): 6794–6803, 10.1021/acs.jpclett.1c01289. 34270259

[46] T. Hofbeck , Y. C. Lam , M. Kalbáč , S. Záliš , A. Vlček Jr. , and H. Yersin , “Thermally Tunable Dual Emission of the d^8^–d^8^ Dimer [Pt_2_(μ‐P_2_O_5_(BF_2_)_2_)_4_]^4–^ ,” Inorganic Chemistry 55 (2016): 2441–2449, 10.1021/acs.inorgchem.5b02839. 26909653

[47] D. Gómez de Segura , A. Corral‐Zorzano , E. Alcolea , M. T. Moreno , and E. Lalinde , “Phenylbenzothiazole‐Based Platinum(II) and Diplatinum(II) and (III) Complexes With Pyrazolate Groups: Optical Properties and Photocatalysis,” Inorganic Chemistry 63 (2024): 1589–1606, 10.1021/acs.inorgchem.3c03532. 38247362 PMC10806813

[48] M. Z. Shafikov , R. Daniels , P. Pander , F. B. Dias , J. A. G. Williams , and V. N. Kozhevnikov , “Dinuclear Design of a Pt(II) Complex Affording Highly Efficient Red Emission: Photophysical Properties and Application in Solution‐Processible OLEDs,” ACS Applied Materials & Interfaces 11 (2019): 8182–8193, 10.1021/acsami.8b18928. 30753060

[49] X. Wu , D.‐G. Chen , D. Liu , et al., “Highly Emissive Dinuclear Platinum(III) Complexes,” Journal of the American Chemical Society 142 (2020): 7469–7479, 10.1021/jacs.9b13956. 32223139

[50] M. Xue , W.‐P. To , G. Cheng , et al., “ ^3^MMLCT Excited States of Luminescent Binuclear Pd^II^ Complexes: Excited State Inner‐Sphere Electron‐Transfer Reactions and Application,” Chemical Science 16 (2025): 10701–10713, 10.1039/D4SC08612K. 40438168 PMC12107753

[51] S.‐W. Lai , T.‐C. Cheung , M. C. W. Chan , K.‐K. Cheung , S.‐M. Peng , and C.‐M. Che , “Luminescent Mononuclear and Binuclear Cyclometalated Palladium(II) Complexes of 6‐Phenyl‐2,2′‐Bipyridines: Spectroscopic and Structural Comparisons With Platinum(II) Analogues,” Inorganic Chemistry 39 (2000): 255–262, 10.1021/ic991089g. 11272533

[52] M. Gao , W.‐P. To , G. S. M. Tong , et al., “Dinuclear Cyclometalated Pincer Nickel(II) Complexes With Metal‐Metal‐to‐Ligand Charge Transfer Excited States and Near‐Infrared Emission,” Angewandte Chemie International Edition 64 (2025): e202414411, 10.1002/anie.202414411. 39320051 PMC11720376

[53] J. Lin , C. Zou , X. Zhang , et al., “Highly Phosphorescent Organopalladium(ii) Complexes With Metal–Metal‐to‐Ligand Charge‐Transfer Excited States in Fluid Solutions,” Dalton Transactions 48 (2019): 10417–10421, 10.1039/C9DT02525A. 31241101

[54] H. B. Gray and A. W. Maverick , “Solar Chemistry of Metal Complexes,” Science 214 (1981): 1201–1205, 10.1126/science.214.4526.1201. 17789279

[55] K. R. Mann , N. S. Lewis , V. M. Miskowski , D. K. Erwin , G. S. Hammond , and H. B. Gray , “Solar Energy Storage. Production of Hydrogen by 546‐nm Irradiation of a Dinuclear Rhodium(I) Complex in Acidic Aqueous Solution,” Journal of the American Chemical Society 99 (1977): 5525–5526, 10.1021/ja00458a071.

[56] M. T. Fortunato , C. E. Moore , and C. Turro , “Ligand‐Centered Photocatalytic Hydrogen Production in an Axially Capped Rh_2_ (II,II) Paddlewheel Complex With Red Light,” Journal of the American Chemical Society 145 (2023): 2748–27357, 10.1021/jacs.3c07532. 38055041

[57] T. J. Whittemore , C. Xue , J. Huang , J. C. Gallucci , and C. Turro , “Single‐Chromophore Single‐Molecule Photocatalyst for the Production of Dihydrogen Using Low‐Energy Light,” Nature Chemistry 12 (2020): 180–185, 10.1038/s41557-019-0397-4. 31959960

[58] P. Gupta , C. E. Moore , and C. Turro , “Photo‐ and Electrocatalytic Hydrogen Evolution by Heteroleptic Dirhodium(II,II) Complexes: Role of the Bridging and Diimine Ligands,” Journal of the American Chemical Society 146 (2024): 27161–27172, 10.1021/jacs.4c10626. 39298379

[59] A. F. Heyduk and D. G. Nocera , “Hydrogen Produced From Hydrohalic Acid Solutions by a Two‐Electron Mixed‐Valence Photocatalyst,” Science 293 (2001): 1639–1641, 10.1126/science.1062965. 11533485

[60] A. J. Esswein and D. G. Nocera , “Hydrogen Production by Molecular Photocatalysis,” Chemical Reviews 107 (2007): 4022–4047, 10.1021/cr050193e. 17927155

[61] T. S. Teets and D. G. Nocera , “Photocatalytic Hydrogen Production,” Chemical Communications 47 (2011): 9268–9274, 10.1039/c1cc12390d. 21647489

[62] V. W.‐W. Yam , V. K.‐M. Au , and S. Y.‐L. Leung , “Light‐Emitting Self‐Assembled Materials Based on d^8^ and d^10^ Transition Metal Complexes,” Chemical Reviews 115 (2015): 7589–7728, 10.1021/acs.chemrev.5b00074. 26158432

[63] K. R. Mann , N. S. Lewis , R. M. Williams , H. B. Gray , and J. G. Gordon II , “Further Studies of Metal‐Metal Bonded Oligomers of Rhodium(I) Isocyanide Complexes. Crystal Structure Analysis of Octakis(Phenyl Isocyanide)Dirhodium Bis(Tetraphenylborate),” Inorganic Chemistry 17 (1978): 828–834, 10.1021/ic50182a008.

[64] K. R. Mann , J. G. Gordon II , and H. B. Gray , “Characterization of Oligomers of Tetrakis(Phenyl Isocyanide)Rhodium(I) in Acetonitrile Solution,” Journal of the American Chemical Society 97 (1975): 3553–3555, 10.1021/ja00845a065.

[65] N. T. Tran , J. R. Stork , D. Pham , et al., “Adventures in Crystallization. Crystalline Salts Containing One, Two, or Even Three Chemically Distinct Cations Obtained From Solutions of [(Cyclohexyl Isocyanide)_4_ Rh^I^]^+^ ,” Inorganic Chemistry 46 (2007): 7998–8007, 10.1021/ic7008013. 17696426

[66] N. T. Tran , J. R. Stork , D. Pham , M. M. Olmstead , J. C. Fettinger , and A. L. Balch , “Variation in Crystallization Conditions Allows the Isolation of Trimeric as Well as Dimeric and Monomeric Forms of [(Alkyl Isocyanide)_4_Rh^I^]^+^ ,” Chemical Communications (2006): 1130–1132, 10.1039/b513700d. 16514462

[67] K. R. Mann , J. A. Thich , R. A. Bell , C. L. Coyle , and H. B. Gray , “Crystal Structure Analyses of Rh_2_(Bridge)_4_(BPh_4_)_2_.CH_3_CN and Rh_2_(TM4‐Bridge)_4_(PF_6_)_2_._2_CH_3_CN. Further Electronic Spectral Studies of Binuclear Rhodium(I) Isocyanide Complexes,” Inorganic Chemistry 19 (1980): 2462–2468, 10.1021/ic50210a054.

[68] V. M. Miskowski , S. F. Rice , H. B. Gray , and S. J. Milder , “Excited‐State Decay Processes of Binuclear Rhodium(I) Isocyanide Complexes,” Journal of Physical Chemistry 97 (1993): 4277–4283, 10.1021/j100119a007.

[69] V. M. Miskowski , G. L. Nobinger , D. S. Kliger , et al., “Flash Kinetic Spectroscopic Studies of Dinuclear Rhodium(I) Complexes,” Journal of the American Chemical Society 100 (1978): 485–488, 10.1021/ja00470a020.

[70] S. F. Rice , S. J. Milder , H. B. Gray , R. A. Goldbeck , and D. S. Kliger , “Photophysical Properties of the Lowest Electronic Excited States of Binuclear Rhodium(I) Isocyanide Complexes,” Coordination Chemistry Reviews 43 (1982): 349–354, 10.1016/S0010-8545(00)82105-0.

[71] J. J. Stace , K. D. Lambert , J. A. Krause , and W. B. Connick , “Rhodium Dimers With 2,2‐Dimethyl‐1,3‐Diisocyano and Bis(Diphenylphosphino)Methane Bridging Ligands,” Inorganic Chemistry 45 (2006): 9123–9131, 10.1021/ic060923g. 17054373

[72] A. L. Balch , “Dimeric Rhodium(I) and Rhodium(II) Complexes With Bridging Phosphine or Arsine Ligands,” Journal of the American Chemical Society 98 (1976): 8049–8054, 10.1021/ja00441a029.

[73] A. L. Balch , J. W. Labadie , and G. Delker , “Further Studies of Diphosphine‐ and Diarsine‐Bridged Rhodium Complexes,” Inorganic Chemistry 18 (1979): 1224–1227, 10.1021/ic50195a009.

[74] A. L. Balch and B. Tulyathan , “Interactions Between Rhodium(I) Centers in Dimeric Complexes,” Inorganic Chemistry 16 (1977): 2840–2845, 10.1021/ic50177a035.

[75] W. A. Fordyce and G. A. Crosby , “Electronic Spectroscopy of Diphosphine‐ and Diarsine‐Bridged Rhodium(I) Dimers,” Journal of the American Chemical Society 104 (1982): 985–988, 10.1021/ja00368a011.

[76] C.‐M. Che , W.‐M. Lee , H.‐L. Kwong , V. W.‐W. Yam , and K.‐C. Cho , “Synthesis, Spectroscopy, and Photochemistry of Bis[Bis(Diphenylphosphino)Methane]Bis(2,5‐Di‐Isocyano‐2,5‐Dimethylhexane)Dirhodium(I),” Journal of the Chemical Society, Dalton Transactions (1990): 1717–1722, 10.1039/dt9900001717.

[77] A. K.‐W. Chan , K. M.‐C. Wong , and V. W.‐W. Yam , “Supramolecular Assembly of Isocyanorhodium(I) Complexes: An Interplay of Rhodium(I)···Rhodium(I) Interactions, Hydrophobic–Hydrophobic Interactions, and Host–Guest Chemistry,” Journal of the American Chemical Society 137 (2015): 6920–6931, 10.1021/jacs.5b03396. 25984814

[78] M. H.‐Y. Chan and V. W.‐W. Yam , “Toward the Design and Construction of Supramolecular Functional Molecular Materials Based on Metal–Metal Interactions,” Journal of the American Chemical Society 144 (2022): 22805–22825, 10.1021/jacs.2c08551. 36484725

[79] A. K.‐W. Chan , M. Ng , K.‐H. Low , and V. W.‐W. Yam , “Versatile Control of Directed Supramolecular Assembly via Subtle Changes of the Rhodium(I) Pincer Building Blocks,” Journal of the American Chemical Society 140 (2018): 8321–8329, 10.1021/jacs.8b04687. 29943985

[80] A. K.‐W. Chan , D. Wu , K. M.‐C. Wong , and V. W.‐W. Yam , “Rhodium(I) Complexes of Tridentate *N*‐Donor Ligands and Their Supramolecular Assembly Studies,” Inorganic Chemistry 55 (2016): 3685–3691, 10.1021/acs.inorgchem.6b00289. 26991111

[81] B. Shi , L. Zhang , K. Yan , et al., “Efficient and Stable NIR‐II Phosphorescence of Metallophilic Molecular Oligomers for In Vivo Single‐Cell Tracking and Time‐Resolved Imaging,” Angewandte Chemie International Edition 63 (2024):e202410118, 10.1002/anie.202410118. 38997791

[82] W. Wei , J. Wang , X. Kang , et al., “Synthesis, Supramolecular Aggregation, and NIR‐II Phosphorescence of Isocyanorhodium(I) Zwitterions,” Chemical Science 14 (2023): 11490–11498, 10.1039/D3SC03508E. 37886099 PMC10599467

[83] Y. Liu , Y. Liu , F. Cheng , et al., “Mechanochromic Phosphorescence of Rhodium(I) Isocyanide Complexes in the NIR‐II Window,” Inorganic Chemistry 64 (2025): 9993–10000, 10.1021/acs.inorgchem.5c00518. 40351266

[84] N. Zhou , Y. Zhang , X. Wang , P. Yang , W. Lu , and Q. Wan , “Effective Near‐Infrared Triplet Emitter Based on Hetero‐Metal–Metal Interaction,” Journal of the American Chemical Society 147 (2025): 19949–19958, 10.1021/jacs.5c04585. 40434357

[85] A. Chatterjee and B. König , “Birch‐Type Photoreduction of Arenes and Heteroarenes by Sensitized Electron Transfer,” Angewandte Chemie International Edition 58 (2019): 14289–14294, 10.1002/anie.201905485. 31379035 PMC6790943

[86] B. Pfund , V. Hutskalova , C. Sparr , and O. S. Wenger , “Isoacridone Dyes With Parallel Reactivity From Both Singlet and Triplet Excited States for Biphotonic Catalysis and Upconversion,” Chemical Science 14 (2023): 11180–11191, 10.1039/D3SC02768F. 37860649 PMC10583676

[87] C. Empel , Q. H. Pham , and R. M. Koenigs , “Spin States Matter─From Fundamentals Toward Synthetic Methodology Development and Drug Discovery,” Accounts of Chemical Research 57 (2024): 2717–2727, 10.1021/acs.accounts.4c00405. 39221592

[88] T. Ziegler and A. Rauk , “Carbon Monoxide, Carbon Monosulfide, Molecular Nitrogen, Phosphorus Trifluoride, and Methyl Isocyanide as σ Donors and п Acceptors. A Theoretical Study by the Hartree‐Fock‐Slater Transition‐State Method,” Inorganic Chemistry 18 (1979): 1755–1759, 10.1021/ic50197a006.

[89] M. V. Kashina , A. A. Karcheuski , M. A. Kinzhalov , K. V. Luzyanin , and S. A. Katkova , “Mutual Placement of Isocyanide and Phosphine Ligands in Platinum(II) Complexes [PtHal_2_L^1^L^2^] (Hal = Cl, Br, I; L^1^,L^2^ = CNCy, PPh_3_) Leads to Highly‐Efficient Photocatalysts for Hydrosilylation of Alkynes,” Molecules 28 (2023): 7764, 10.3390/molecules28237764. 38067492 PMC10708205

[90] V. M. Miskowski , T. P. Smith , T. M. Loehr , and H. B. Gray , “Properties of Metal‐Metal Single Bonds. Vibrational and Electronic Spectra of Binuclear Rhodium(II) and Iridium(II) Isocyanide Complexes With Comparisons to Decacarbonyldimanganese [Mn_2_(CO)_10_],” Journal of the American Chemical Society 107 (1985): 7925–7934, 10.1021/ja00312a021.

[91] S. F. Rice , V. M. Miskowski , and H. B. Gray , “Electronic Absorption Spectra of Binuclear Rhodium(I) Isocyanide Complexes. Comparison of Ground‐State and dσ* → pσ Excited‐State Bond Energies,” Inorganic Chemistry 27 (1988): 4704–4708, 10.1021/ic00299a006.

[92] H. B. Gray , S. Záliš , and A. Vlček , “Electronic Structures and Photophysics of d^8^‐d^8^ Complexes,” Coordination Chemistry Reviews 345 (2017): 297–317, 10.1016/j.ccr.2017.01.008.

[93] A. J. Bukvic , M. Brändlin , D. Häussinger , and O. S. Wenger , “Supramolecular Assembly of a Macrocyclic Rhodium(I) Isocyanide Complex With Long‐Lived Near‐Infrared Luminescence,” Journal of the American Chemical Society 147 (2025): 43540–43549, 10.1021/jacs.5c12755. 41231408

[94] R. Monni , G. Capano , G. Auböck , et al., “Vibrational Coherence Transfer in the Ultrafast Intersystem Crossing of a Diplatinum Complex in Solution,” PNAS 115 (2018): E6396–E6403, 10.1073/pnas.1719899115. 29941568 PMC6048523

[95] H. Xiang , J. Cheng , X. Ma , X. Zhou , and J. J. Chruma , “Near‐Infrared Phosphorescence: Materials and Applications,” Chemical Society Reviews 42 (2013): 6128–6185, 10.1039/c3cs60029g. 23652863

[96] P. Łaski , L. Bosman , J. Drapała , et al., “Nanosecond‐Lived Excimer Observation in a Crystal of a Rhodium(I) Complex via Time‐Resolved X‐Ray Laue Diffraction,” Journal of Physical Chemistry Letters 15 (2024): 10301–10306, 10.1021/acs.jpclett.4c02476. 39382182 PMC11492376

[97] J. B. Benedict , A. Makal , J. D. Sokolow , et al., “Time‐Resolved Laue Diffraction of Excited Species at Atomic Resolution: 100 Ps Single‐Pulse Diffraction of the Excited state of the Organometallic Complex Rh_2_(μ‐PNP)_2_(PNP)_2_·BPh_4_ ,” Chemical Communications 47 (2011): 1704–1706, 10.1039/c0cc04997b. 21210070 PMC3129623

[98] S. F. Rice and H. B. Gray , “Metal‐Metal Interactions in Binuclear Rhodium Isocyanide Complexes. Polarized Single‐Crystal Spectroscopic Studies of the Lowest Triplet ← Singlet System in Tetrakis(1,3‐Diisocyanopropane)Dirhodium(2+),” Journal of the American Chemical Society 103 (1981): 1593–1595, 10.1021/ja00396a065.

[99] M. Iwamura , K. Nozaki , S. Takeuchi , and T. Tahara , “Real‐Time Observation of Tight Au–Au Bond Formation and Relevant Coherent Motion Upon Photoexcitation of [Au(CN)_2_–] Oligomers,” Journal of the American Chemical Society 135 (2013): 538–541, 10.1021/ja310004z. 23273414

[100] J. V. Caspar , E. M. Kober , B. P. Sullivan , and T. J. Meyer , “Application of the Energy Gap Law to the Decay of Charge‐Transfer Excited States,” Journal of the American Chemical Society 104 (1982): 630–632, 10.1021/ja00366a051.

[101] M. Montalti , A. Credi , L. Prodi , and M. T. Gandolfi , Handbook of Photochemistry (CRC Press, 2006), 10.1201/9781420015195.

[102] B. Ma , J. Li , P. I. Djurovich , M. Yousufuddin , R. Bau , and M. E. Thompson , “Synthetic Control of Pt···Pt Separation and Photophysics of Binuclear Platinum Complexes,” Journal of the American Chemical Society 127 (2005): 28–29, 10.1021/ja044313w. 15631432

[103] K. Saito , Y. Nakao , and S. Sakaki , “Theoretical Study of Pyrazolate‐Bridged Dinuclear Platinum(II) Complexes: Interesting Potential Energy Curve of the Lowest Energy Triplet Excited State and Phosphorescence Spectra,” Inorganic Chemistry 47 (2008): 4329–4337, 10.1021/ic702367f. 18416550

[104] S. J. Milder , R. A. Goldbeck , D. S. Kliger , and H. B. Gray , “Studies of Energy‐Transfer and Electron‐Transfer Processes Involving the ^3^A_2u_ Excited States of Binuclear Rhodium Isocyanide Complexes,” Journal of the American Chemical Society 102 (1980): 6761–6764, 10.1021/ja00542a015.

[105] D. S. Wagner , L. Spierling , and O. S. Wenger , “Expanding Thermodynamic and Kinetic Frontiers in Molecular Photocatalysis,” ACS Central Science 12 (2026): 144–156, 10.1021/acscentsci.5c02047. 41783129 PMC12956032

