# Supplementary material for: Controlling Near‐Infrared Fluorescence‐to‐Phosphorescence Ratios and Triplet Lifetimes in Rhodium(I) Dimers via Primary and Secondary Coordination Sphere Effects
Source: Angew Chem Int Ed Engl. 2026 Apr 9;65(21):e6066376. doi: 10.1002/anie.6066376 (PMC13182202; doi:10.1002/anie.6066376)
Supplement: Supplementary file 1 — Supporting File 1: anie72080‐sup‐0001‐SuppMat.docx. [file ANIE-65-e6066376-s002.docx]

Controlling Near-Infrared Fluorescence-to-Phosphorescence Ratios and Triplet Lifetimes in Rhodium(I) Dimers via Primary and Secondary Coordination Sphere Effects

Vanitha R. Naina,^*[a]^ Giacomo Morselli,^[a]†^ Alessandro Prescimone,^[b]†^ Daniel Häussinger,^[a]^ and Oliver S. Wenger^*[a]^

^[a]^ Department of Chemistry, University of Basel, St. Johanns-Ring 19, 4056 Basel, Switzerland.

^[b]^ Department of Chemistry, University of Basel, BPR 1096, Mattenstrasse 22, 4058 Basel, Switzerland.

^†^ Current Address: Department of Chemistry, University of Basel, BPR 1096, Mattenstrasse 22, 4058 Basel, Switzerland.

^*^Correspondence:

Vanitha R. Naina: vanithareddy.naina@unibas.ch

Oliver S. Wenger: oliver.wenger@unibas.ch

Table of Contents

[**Experimental details** S3](#_Toc218854550)

[**Materials and methods** S3](#_Toc218854551)

[**Synthesis and characterization** S5](#_Toc218854552)

[**Synthesis and characterization of bridging diisocyanide ligand L*^t^*^Bu^** S5](#_Toc218854553)

[**Synthesis and characterization of homoleptic rhodium(I) complex [Hom-Rh_2_]*^t^*^Bu^** S8](#_Toc218854554)

[**Synthesis and characterization of heteroleptic rhodium(I) complex [Het-Rh_2_]*^t^*^Bu^** S9](#_Toc218854555)

[**Synthesis and characterization of bridging diisocyanide ligand L^Me^** S10](#_Toc218854556)

[**Synthesis and characterization of heteroleptic rhodium(I) complex [Het-Rh_2_]^Me^** S12](#_Toc218854557)

[**Synthesis and characterization of bridging diisocyanide ligand L*^t^*^Bu2^** S13](#_Toc218854558)

[**Synthesis and characterization of heteroleptic rhodium(I) complex [Het-Rh_2_]*^t^*^Bu2^** S16](#_Toc218854559)

[**Synthesis and characterization of bridging diisocyanide ligand L^Mes^** S17](#_Toc218854560)

[**Synthesis and characterization of homoleptic rhodium(I) complex [Hom-Rh_2_]^Mes^** S20](#_Toc218854561)

[**NMR spectra** S21](#_Toc218854562)

[**HRMS-ESI spectra** S39](#_Toc218854563)

[**Infrared spectra** S49](#_Toc218854564)

[**X-ray crystallography** S52](#_Toc218854565)

[**UV-Vis steady state absorption spectroscopy** S61](#_Toc218854566)

[**Photoluminescence spectroscopy** S62](#_Toc218854567)

[**Luminescence quantum yield determination** S67](#_Toc218854568)

[**Nanosecond transient absorption measurements** S76](#_Toc218854569)

[**Femtosecond transient absorption measurements** S79](#_Toc218854570)

[**Cyclic voltammetry** S81](#_Toc218854571)

[**References** S82](#_Toc218854572)

# **Experimental details**

## **Materials and methods**

Deuterated solvents were acquired from Cambridge Isotope Laboratories Inc. or Apollo Scientific. All reagents and substrates not involving synthetic procedures in this study were obtained from Thermo Scientific, Fluorochem, Alfar Aesar, Acros Organics, or Sigma-Aldrich/Merck, with a minimum purity of "reagent grade," and were used as received. Solvents for spectroscopic measurements were purchased as “extra dry” with 99.8% purity from Thermo Scientific or Sigma-Aldrich, and were filtered prior to use. Unless otherwise noted all chemicals were purchased from commercial suppliers and used without further purification.

Thin layer chromatography (TLC) was performed on pre-coated aluminum plates (Merck: silica gel 60 with a fluorescence indicator F254, layer thickness of 0.25 mm). Compound visualization was done under UV light, using either the 254 nm or 365 nm wavelength from a UV lamp. For the isolation of reaction products via column chromatography, silica gel (Silicycle: silica flash® P60, 40-63 μm, 230-400 mesh) was used.

NMR spectra were recorded using a Bruker Avance III spectrometer operating at proton frequencies of 250, 400, 500 or 600 MHz. All measurements were taken at 298 K in 5 mm diameter tubes. Residual protio solvent resonances were used as an internal reference for ^1^H and ^13^C{^1^H} NMR spectra, and externally referenced to SiMe_4_. ^31^P{^1^H} NMR spectra were referenced externally to 85% H_3_PO_4_ (D_2_O). Coupling patterns were denoted as follows: s (singlet), d (doublet), dd (doublet of doublets), t (triplet), and m (multiplet), with coupling constants (J) expressed in Hertz (Hz).

Elemental analysis was performed by Sylvie Mittelheisser, with a Vario Micro Cube instrument from Elementar. HRMS analyses were conducted by Dr. Michael Pfeffer on a Bruker maxis 4G ESI-Q-TOF under direct injection conditions with CH_3_CN as solvent.

Solid-state IR spectroscopy (FTIR) was performed on a Bruker Alpha Platinum-ATR instrument.

Cyclic voltammetry was performed under N_2_ atmosphere in an electrochemical cell. A glassy carbon disc electrode, a silver wire, and a saturated calomel electrode (SCE) were used as working, counter, and reference electrodes, respectively. Dry dichloromethane (Aldrich) was used without further purification. Sample solutions (1 mM) with supporting electrolyte (0.1 M, TBAPF_6_; TBA = tetra-*n*-butylammonium) were prepared and solutions were bubbled with N_2_ for 5 min prior to each measurement. The sweeping rate of 100 mV·s^−1^ was controlled and the response current signals were recorded on a Versastat 3-200 potentiostat (Princeton Applied Research).

Sample preparation for spectrophotometric measurements was performed in screw cap quartz cuvettes. All solutions were purged with argon to remove oxygen and sealed under argon with septum caps. Optical spectroscopic studies were conducted at 293 K. Steady-state UV-Vis absorption spectra were recorded using a Cary 5000 spectrophotometer (Agilent Technologies).

Steady-state emission and excitation spectra were measured on a Fluorolog3-22 instrument from Horiba Jobin-Yvon, equipped with a Xenon lamp 450-Watt Illuminator (FL-1039A/40A), a water-cooled photomultiplier tube (PMT Hamamatsu R2658 or R928) and a DSS LN solid state detector for measurements in the near infrared region. The luminescence spectra were corrected for the spectral response of the system.

Photoluminescence lifetime studies were performed using a LifeSpecII from Edinburgh Instruments using a pulsed LED for excitation at 635.2 nm (Edinburgh Instruments, pulse width: 61.4 ps), where the cuvette holder was equipped with a temperature controller (TC 1, Quantum Northwest). Light emission was accumulated *via* time-correlated single photon counting (TCSPC). Typical error values for spectroscopic measurements: 5% for molar absorptivity and lifetimes, 10% for photoluminescence quantum yields.

Transient UV-Vis absorption spectroscopy with nanosecond time resolution was performed on an LP920-KS apparatus from Edinburgh Instruments. A frequency-tripled Nd:YAG laser (Quantel Brilliant, ca. 10 ns pulse width) equipped with an OPO from Opotek was used for excitation. An iCCD camera from Andor was used to detect transient absorption spectra, and single-wavelength kinetics were recorded with a photomultiplier tube.

Transient UV-Vis absorption spectroscopy with sub-picosecond time resolution was measured using a HARPIA-TA instrument (Light Conversion). In this experimental setup, the excitation light is generated by a PHAROS laser (Light Conversion, Yb:KGW laser, source wavelength = 1030 nm, pulse duration = ~190 fs, repetition rate = 5.0 kHz, output power = 1.0 W, pulse energy = 0.2 mJ), and the actual pump light wavelength was generated by an optical parametric amplifier called ORPHEUS (Light Conversion, used ~90% of fundamental pulse). The probe light was generated by a sapphire (5 mm thickness; ~10% of the fundamental pulse was used to generate a white light super-continuum). Sample solutions (absorbance = ~0.2 at the excitation wavelength) were measured in a 1 mm quartz cuvette at room temperature. The obtained transient absorption spectra were analyzed using the CarpetView software (Light Conversion).

## **Synthesis and characterization**

### **Synthesis and characterization of bridging diisocyanide ligand L*^t^*^Bu^**

Scheme S1: Synthesis of bridging isocyanide ligand **L^tBu^**.

**Synthesis of S1:**

To a round-bottom flask, 2,2′-oxydianiline (2.0 g, 9.99 mmol) was dissolved in 100 mL of dimethylformamide (DMF). The solution was cooled to 0 °C, and N-bromosuccinimide (3.91 g, 21.97 mmol, 2.2 eq) was added portionwise. The reaction mixture was then stirred overnight at room temperature. After completion, 100 mL of ethyl acetate and 100 mL of distilled water were added, and the organic layer was separated. The organic phase was washed twice with water, followed by a wash with brine, and then dried over anhydrous Na₂SO₄. The crude product was purified by column chromatography using a mixture of dichloromethane (CH₂Cl₂) and hexane as the eluent. Yield: 1.94 g (54 %).

**^1^H NMR (500 MHz, CDCl_3_):** *δ* (ppm) = 7.07 (dd, ^3^*J*(H-H) = 8.6, ^4^*J*(H-H) = 2.2 Hz, 2H; *H*-3), 6.89 (d, ^4^*J*(H-H) = 2.2 Hz, 2H; *H*-5), 6.69 (d, ^3^*J*(H-H) = 8.6 Hz, 2H; *H*-2), 3.87 (br, 4H, N*H*_2_).

**^13^C{^1^H} NMR (126 MHz, CDCl_3_):** *δ* (ppm) *=* 143.8 (*C*-6), 137.1 (*C*-1), 127.7 (*C*-3), 121.3 (*C*-5), 117.5 (*C*-2), 109.6 (*C*-4).

**HRMS:** Calculated m/z for [C_12_H_10_Br_2_N_2_O+H]^+^: 358.9213, found: 358.9217.

**Synthesis of S2:**

To a 250 mL Schlenk flask, 90 mL of acetic anhydride was added and cooled to 0 °C. Formic acid (45 mL) was then added dropwise to the flask, and the reaction mixture was degassed by bubbling nitrogen through it for 15 minutes. The mixture was subsequently heated to 50 °C and stirred for 2 hours. After cooling the reaction back to 0 °C, the solid substrate (1.5 g, 4.19 mmol) was added portionwise. The mixture was stirred overnight at room temperature and then poured into 1 L of ice-cold water. The resulting precipitate was collected by filtration, washed with additional cold water, and dried under reduced pressure. Yield: 1.68 g (97 %).

**^1^H NMR (400 MHz, DMSO):** *δ* (ppm) *=* 10.22 – 9.88 (m, 2H), 8.73 – 8.07 (m, 3.84H), 7.52 – 7.30 (m, 2.27H), 7.21 – 6.95 (m, 2H).

The NMR assignments were not performed due to the observation of several rotamers (formamide) in the solution.

**HRMS** Calculated m/z for [C_14_H_10_N_2_O_3_Br_2_+H]^+^: 412.9131, found: 412.9129.

**Synthesis of S3:**

To a two-neck flask equipped with a reflux condenser, formamide **S2** (850 mg, 2.05 mmol), (3,5-di-*tert*-butylphenyl)boronic acid (1.49 g, 6.2 mmol, 3.0 equiv), and K_3_PO_4_ (3.05 g, 14.4 mmol, 7.0 equiv) were added and suspended in a mixture of tetrahydrofuran (THF) and water (THF:H₂O, 24:30 mL). The resulting suspension was degassed for 15 minutes, after which PdXPhosG3 (139.0 mg, 0.16 mmol, 0.08 equiv) was added, and the mixture was further degassed for an additional 5 minutes. The reaction mixture was then heated to reflux and stirred overnight.

After cooling to room temperature, 50 mL of ethyl acetate and 50 mL of distilled water were added. The organic layer was separated and washed successively with 1 M NaOH solution and brine. The combined organic extracts were dried over anhydrous sodium sulfate (Na_2_SO_4_), filtered, and concentrated under reduced pressure. The crude solid was purified by silica gel column chromatography using ethyl acetate and cyclohexane mixture in a 1:1 ratio. Yield: 1.1 g (85 %)

**^1^H NMR (400 MHz, CDCl_3_):** *δ* (ppm) = 8.91 – 8.78 (m, 0.59H), 8.57 – 8.28 (m, 2.41H), 8.25 (d, *J* = 8.4 Hz, 1.10H), 8.10 (s, 1.11H), 7.99 (s, 0.4H), 7.47 – 7.33 (m, 3.94H), 7.25 – 7.17 (m, 3.49H), 7.10 (s, 1.32H), 7.04 (s, 0.39H), 1.31 (s, 36H).

The NMR assignments were not performed due to the observation of several rotamers (formamide) in the solution.

**HRMS** Calculated m/z for [C_42_H_52_N_2_O_3_+H]^+^: 633.4051, found: 633.4055.

**Synthesis of L*^t^*^Bu^:**

A 100 mL Schlenk flask was charged with formamide **S3** (400 mg, 0.63 mmol) and 25 mL of dry CH_2_Cl_2_. Diisopropylamine (326.2 mg, 3.2 mmol, 5.1 equiv) was then added to the solution under an inert atmosphere. The reaction mixture was cooled to 0 °C, and POCl_3_ (494.2 mg, 3.2 mmol, 5.1 equiv) was added dropwise under the same inert conditions. After stirring at room temperature for 4 hours, 15 mL of 1.5 M Na_2_CO_3_ solution was added, and the mixture was stirred overnight.

The reaction was quenched by adding 25 mL of CH_2_Cl_2_ and 25 mL of distilled water. The organic layer was separated and subsequently washed with 25 mL of saturated NaHCO_3_ solution, followed by a brine wash. The organic phase was dried over anhydrous Na_2_SO_4_, filtered, and then dried to obtain the crude product. The crude was purified by column chromatography using ethyl acetate and petroleum ether. Yield: 169 mg (45 %)

**^1^H NMR (500 MHz, CD_2_Cl_2_):** *δ* (ppm) = 7.57 (d, *J* = 8.3 Hz, 2H, *H*-2), 7.46 (t, *J* = 1.8 Hz, 2H, *H*-10), 7.43 (dd, *J* = 8.3, 1.8 Hz, 2H, *H*-3), 7.30 (d, ^4^*J*(H-H) = 1.8 Hz, 4H, *H*-8), 7.23 (d, ^4^*J*(H-H) = 1.8 Hz, 2H, *H*-5), 1.32 (s, 36H, *H*-12).

**^13^C{^1^H} NMR (126 MHz, CD_2_Cl_2_):** *δ* (ppm) = 170.7 (N*C*), 152.1 (*C*-9), 151.7 (*C*-6), 146.0 (*C*-4), 138.7(*C*-7), 128.9 (*C*-2), 124.1 (*C*-3), 123.2 (*C*-10), 121.9 (*C*-8), 118.3 (*C*-5), 117.6 (*C*-1), 35.3 (*C*-11), 31.5 (*C*-12).

**EA**: Anal. Calc. for [C_42_H_48_N_2_O]·[0.3CH_2_Cl_2_] (%): C 81.64; H 7.87; N 4.50; Found: C 81.92; H 8.08; N 4.70.

**HRMS** Calculated m/z for [C_42_H_48_N_2_O+Na]^+^: 619.3659, found: 619.3655.

**FTIR:** **ν** (cm^-1^) 2967, 2933, 2907 2864, 2118 (ν_C≡N_), 1608, 1596, 1561, 1501, 1473, 1451, 1429, 1391, 1360, 1326, 1286, 1260, 1248, 1224, 1205, 1181, 1124, 1071, 1021, 969, 930, 899, 882, 875, 860, 835, 821, 780, 773, 711, 673, 658, 645, 599, 591, 572, 540, 512, 489, 461.

### **Synthesis and characterization of homoleptic rhodium(I) complex [Hom-Rh_2_]*^t^*^Bu^**

Scheme S2: Synthesis of **[Hom-Rh_2_]^tBu^**.

An oven-dried Schlenk tube was charged with ligand **L*^t^*^Bu^** (30 mg, 0.05 mmol, 2.1 eq) and [Rh(cod)_2_]BF_4_ (9 mg, 0.02 mmol, 1.0 eq), then evacuated before the addition of 5 mL CH_2_Cl_2_. The reaction mixture was stirred overnight, after which the volatiles were removed under reduced pressure. The resulting crude solid was washed with cold n-pentane and dried under reduced pressure to obtain an analytically pure complex. Yield: 25 mg (36 %).

**^1^H NMR (500 MHz, CD_3_CN):** *δ* (ppm) = 7.72 (d, *J* = 1.6 Hz, 8H, *H*-5), 7.49 (t, *J* = 1.8 Hz, 8H, *H*-10), 7.26 (d, *J* = 1.8 Hz, 16H, *H*-8), 7.24 – 7.19 (m, 16H, *H*-2 and *H*-3), 1.22 (s, 144H, *H*-12).

**^13^C{^1^H} NMR (126 MHz, CD_3_CN):** *δ* (ppm) = 152.8 (*C*-9), 152.1 (*C*-6), 147.1 (*C*-4), 139.0 (*C*-7), 127.9 (*C*-2), 124.5 (*C*-10), 124.3 (*C*-3), 122.4 (*C*-8), 118.1 (*C*-5, assigned based on ^13^C DEPT-135), 117.3 (*C*-1), 35.6 (*C*-11), 31.6 (*C*-12).

**EA**: Anal. Calc. for [C_168_H_192_N_8_O_4_Rh_2_B_2_F_8_]·[1CH_2_Cl_2_] (%): C 71.18; H 6.86; N 3.93; Found: C 71.42; H 7.14; N 3.67.

**HRMS** Calculated m/z for [C_168_H_192_N_8_O_4_Rh_2_]^2+^: 1295.6583, found: 1295.6588.

**FTIR:** ν (cm^-1^) 2954, 2904, 2868, 2146 (ν_C≡N_), 1597, 1564, 1496, 1429, 1392, 1363, 1322, 1249, 1187, 1124, 1053, 966, 926, 898, 863, 817, 752, 711, 654, 565, 519, 457.

### **Synthesis and characterization of heteroleptic rhodium(I) complex [Het-Rh_2_]*^t^*^Bu^**

Scheme S3: Synthesis of **[Het-Rh_2_]^tBu^**.

An oven-dried Schlenk tube was charged with ligand **L*^t^*^Bu^** (50 mg, 0.08 mmol, 1.0 eq), bis(diphenylphosphino)methane (32.2 mg, 0.08 mmol, 1.0 eq), and [Rh(cod)_2_]BF_4_ (34.0 mg, 0.08 mmol, 1.0 eq), then evacuated before the addition of 5 mL acetonitrile. The reaction mixture was stirred overnight, after which the volatiles were removed under reduced pressure. The resulting crude solid was washed with diethyl ether and purified by crystallization from dichloromethane and n-hexane. Single crystals suitable for X-ray diffraction were obtained by slow diffusion of diethyl ether into a concentrated acetonitrile solution at room temperature. Yield: 93 mg (47 %)

**^1^H NMR (500 MHz, CD_3_CN):** *δ* (ppm) = 8.17 – 7.77 (m, 16H, *H*-15), 7.51 (t, ^3^*J*(H-H) = 1.8 Hz, 4H, *H*-10), 7.47 (d, ^3^*J*(H-H) = 1.8 Hz, 4H, *H*-5), 7.34 (dd, ^3^*J*(H-H) = 8.4, ^4^*J*(H-H) = 1.8 Hz, 4H, *H*-3), 7.31 (d, *J* = 1.8 Hz, 8H, *H*-8), 7.26 (m, 24H, *H*-16 and *H*-17), 6.68 (d, ^4^*J*(H-H) = 8.4 Hz, 4H, *H*-2), 4.17 – 4.06 (m, 4H, *H*-13), 1.28 (s, 72H, *H*-12).

**^31^P{^1^H} NMR (202 MHz, CD_3_CN):** *δ* (ppm) = 23.9 – 22.9 (m).

**^13^C{^1^H} NMR (126 MHz, CD_3_CN):** *δ* (ppm) = 152.8 (*C*-9), 150.8 (*C*-6), 146.0 (*C*-4), 138.9 (*C*-7), 136.2 – 135.8 (m, *C*-14, coupling with distinct ^31^P and ^103^Rh nuclei), 133.5 – 133.4 (m, *C*-15, coupling with distinct ^31^P and ^103^Rh nuclei), 131.9 (*C*-17), 129.9 – 129.8 (m, *C-*16, coupling with distinct ^31^P and ^103^Rh nuclei), 128.2 (*C*-2), 124.6 (*C*-3), 124.2 (*C*-10), 122.3 (*C*-8), 118.5 (*C*-1), 35.7 (*C*-11), 31.6 (*C*-12), 29.5 – 29.3 (m, *C*-12, coupling with distinct ^31^P and ^103^Rh nuclei leads to multiple splitting, resulting in a weak signal with poor resolution). Isocyanide carbon could not be detected. Clear splitting of *C*-15 and *C*-16 was only observed in the ^13^C DEPT-135 spectrum.

**HRMS** Calculated m/z for [C_134_H_140_N_4_O_2_P_4_Rh_2_]^2+^: 1083.4013, found: 1083.4034

**EA**: Anal. Calc. for [C_134_H_140_N_4_O_2_P_4_Rh_2_B_2_F_8_]·[1CH_2_Cl_2_] (%): C, 66.81; H, 5.90; N, 2.31; Found: C, 66.83; H, 6.24; N, 2.17.

**FTIR: ν** (cm^-1^) 2990, 2906, 2866, 2111 (ν_C≡N_), 1597, 1561, 1497, 1482, 1435, 1393, 1363, 1330, 1316, 1278, 1190, 1093, 1054, 998, 967, 924, 899, 828, 785, 735, 694, 659, 561, 515, 478, 446.

### **Synthesis and characterization of bridging diisocyanide ligand L^Me^**

Scheme S4: Synthesis of bridging isocyanide ligand **L^Me^**.

**Synthesis of S4:**

To a two-neck flask equipped with a reflux condenser, formamide **S2** (966 mg, 2.33 mmol), 3,5-dimethylphenylboronic acid (1.08 g, 7.0 mmol, 3.0 equiv), and K_3_PO_4_ (3.47 g, 16.3 mmol, 7.0 equiv) were added and suspended in a mixture of tetrahydrofuran and water (THF:H_2_O, 24:30 mL). The resulting suspension was degassed for 15 minutes, after which PdXPhosG3 (157.99 mg, 0.19 mmol, 0.08 equiv) was added, and the mixture was further degassed for an additional 5 minutes. The reaction mixture was then heated to reflux and stirred overnight.

After cooling to room temperature, 50 mL of ethyl acetate and 50 mL of distilled water were added. The organic layer was separated and washed successively with 1 M NaOH solution and brine. The combined organic extracts were dried over anhydrous sodium sulfate (Na_2_SO_4_), filtered, and concentrated under reduced pressure. The resulting crude solid was dissolved in 10 mL of ethyl acetate, and the pure product was precipitated by the addition of cyclohexane. The solid was collected by filtration and dried under reduced pressure. Yield: 822 mg (76 %).

**^1^H NMR (400 MHz, CDCl_3_):** *δ* (ppm) = 9.36 (d, *J* = 11.3 Hz, 0.18H), 9.13 (d, *J* = 11.3 Hz, 0.38H), 8.80 (m, 0.55H), 8.58 (s, 0.39H), 8.50 (d, *J* = 1.8 Hz, 0.41H), 8.47 – 8.39 (m, 1.51H), 8.35 – 8.26 (m, 2.17H), 7.41 – 7.33 (m, 2.62H), 7.21 (m, 0.53H), 7.13 (d, *J* = 2.0 Hz, 0.39H), 7.10 (d, *J* = 2.0 Hz, 1.10H), 7.06 (s, 3.90H), 6.97 (m, 2H), 2.34 – 2.31 (m, 12H). The NMR assignments were not performed due to the observation of several rotamers (formamide) in the solution.

**HRMS** Calculated m/z for [C_30_H_28_N_2_O_3_+H]^+^: 465.2173, found: 465.2171.

**Synthesis of L^Me^:**

A 100 mL Schlenk flask was charged with formamide **S4** (400 mg, 0.86 mmol) and 40 mL of dry CH_2_Cl_2_. Diisopropylamine (443 mg, 4.4 mmol, 5.1 equiv) was then added to the solution under an inert atmosphere. The reaction mixture was cooled to 0 °C, and POCl_3_ (673 mg, 4.4 mmol, 5.1 equiv) was added dropwise under the same inert conditions. After stirring at room temperature for 4 hours, 15 mL of 1.5 M Na_2_CO_3_ solution was added, and the mixture was stirred overnight.

The reaction was quenched by adding 50 mL of CH_2_Cl_2_ and 50 mL of distilled water. The organic layer was separated and subsequently washed with 50 mL of saturated NaHCO_3_ solution, followed by a brine wash. The organic phase was dried over anhydrous Na_2_SO_4_, filtered, and passed through a plug of silica to remove impurities. The crude product was then dried and redissolved in a minimal amount of ethyl acetate. Upon addition of petroleum ether, the pure product precipitated as a white solid, which was collected by filtration and dried under reduced pressure. Yield: 215 mg (58%)

**^1^H NMR (500 MHz, CDCl_3_):** *δ* (ppm) = 7.53 (d, ^3^*J*(H-H) = 8.3 Hz, 2H, *H*-2), 7.39 (dd, ^3^*J*(H-H) = 8.3, ^4^*J*(H-H) = 1.9 Hz, 2H, *H*-3), 7.16 (d, ^4^*J*(H-H) = 1.8 Hz, 2H, *H*-5), 7.09 (s, 4H, *H*-8), 7.02 (s, 2H, *H*-10), 2.35 (s, 12H, *H*-11).

**^13^C{^1^H} NMR (126 MHz, CDCl_3_):** *δ* (ppm) = 170.1 (N-*C*), 151.5 (*C*-6), 144.7 (*C*-4), 138.9 (*C*-7), 138.8 (*C*-9) 130.4 (*C*-10), 128.6 (*C*-2), 125.2 (*C*-8), 123.6 (*C*-3), 117.7 (*C*-5), 117.5 (*C*-1), 21.5 (*C*-11).

**EA**: Anal. Calc. for [C_42_H_48_N_2_O]·[0.2C_4_H_8_O_2_] (EtOAc) (%): C 83.51; H 5.62; N 6.45; Found: C 83.38; H 5.55; N 6.42.

**HRMS** Calculated m/z for [C_30_H_24_N_2_O+H]^+^: 429.1961, found: 429.1959

**FTIR: ν** (cm^-1^) 2914m 2851, 2123 (ν_C≡N_), 1606, 1568, 1506, 1471, 1427, 1388, 1375, 1316, 1247, 1165, 1126, 1074, 1036, 995, 957, 880, 848, 809, 786, 709, 686, 658, 631, 593, 568, 535, 499, 452

### **Synthesis and characterization of heteroleptic rhodium(I) complex [Het-Rh_2_]^Me^**

Scheme S5: Synthesis of **[Hom-Rh_2_]^Me^**.

An oven-dried Schlenk tube was charged with ligand **L^Me^** (50.5 mg, 0.12 mmol, 1.0 eq), bis(diphenylphosphino)methane (45.3 mg, 0.12 mmol, 1.0 eq), and [Rh(cod) _2_]BF_4_ (47.8 mg, 0.12 mmol, 1.0 eq), then evacuated before the addition of 10 mL dichloromethane. The reaction mixture was stirred overnight, after which the volatiles were removed under reduced pressure. The resulting crude solid was washed with diethyl ether and purified by crystallization from dichloromethane and n-hexane. Single crystals suitable for X-ray diffraction were obtained by layering n-hexane on a concentrated dichloromethane solution of **[Het-Rh_2_]^Me^**. Yield: 93 mg (44 %)

**^1^H NMR (500 MHz, CD_3_CN):** *δ* (ppm) = 7.92 (m, 16H, *H*-14), 7.48 (d, ^4^*J*(H-H) = 1.8 Hz, 4H, *H*-5), 7.31 (dd, ^4^*J*(H-H) = 8.4, ^3^*J*(H-H) = 1.8 Hz, 4H, *H*-3), 7.26 – 7.20 (m, 32H, *H*-8*, H*-15 and *H*-16), 7.12 – 7.08 (m, 4H, *H*-10), 6.69 (d, ^4^*J*(H-H) = 8.4 Hz, 4H, *H*-2), 4.12 – 4.03 (m, 4H, *H*-12), 2.35 (s, 24H, *H*-11).

**^31^P{^1^H} NMR (202 MHz, CD_3_CN):** *δ* (ppm) = 24.1-23.1 (m).

**^13^C{^1^H} NMR (126 MHz, CD_3_CN):** *δ* (ppm) = 150.6 (*C*-6), 145.2 (*C*-4), 139.9 (*C*-9), 139.3 (*C*-7), 136.0 (m, *C*-13, coupling with distinct ^31^P and ^103^Rh nuclei), 133.8 – 133.0 (m, *C-*14, coupling with distinct ^31^P and ^103^Rh nuclei), 131.9 (*C*-16), 131.4 (*C*-10), 130.0-129.6 (m, *C*-15, coupling with distinct ^31^P and ^103^Rh nuclei), 128.3 (*C*-2), 126.0 (*C*-8), 124.2 (*C*-3), 118.1 (*C*-1), 117.5 (*C*-5), 29.5 – 29.1 (m, *C-*12, coupling with distinct ^31^P and ^103^Rh nuclei leads to multiple splitting, resulting in a weak signal with poor resolution), 21.4 (*C*-11). Isocyanide carbon could not be detected.

**EA**: Anal. Calc. for [C_110_H_92_N_4_O_2_P_4_Rh_2_B_2_F_8_]·[0.7CH_2_Cl_2_] (%): C 64.40; H 4.56; N 2.71; Found: C 64.60; H 5.00; N 2.47.

**HRMS** Calculated m/z for [C_110_H_92_N_4_O_2_P_4_Rh_2_]^2+^: 915.2135, found: 915.2137.

**FTIR:** ν (cm^-1^) 2918, 2853, 2111 (ν_C≡N_), 1603, 1570, 1473, 1393, 1330, 1308, 1242, 1178, 1052, 996, 957, 822, 780, 735, 692, 631, 515, 480.

### **Synthesis and characterization of bridging diisocyanide ligand L*^t^*^Bu2^**

Scheme S6: Synthesis of the ligand **L^tBu2^**.

**Synthesis of S5:**

(2,2’-Oxy)dianiline (1.0 g, 4.99 mmol) was weighed in a two-neck flask equipped with a dropping funnel. The solid was dissolved in AcOH (90 mL). Then, to the dropping funnel, AcOH (30 mL) and bromine (1.13 mL, 22.0 mmol, 4.4 equiv) were added, and the resulting mixture was added dropwise to the flask. The reaction mixture was then stirred for 3 hours at room temperature

After 3 h, the reaction mixture was poured into an Erlenmeyer flask containing 250 mL ice-cold distilled water. DCM was added to the Erlenmeyer flask, and the organic layer was separated and washed successively with distilled water. The organic extracts were dried over anhydrous sodium sulfate (Na_2_SO_4_), filtered, and concentrated under reduced pressure. Yield: 2.51 g (72%).

**^1^H NMR (500 MHz, CDCl_3_):** *δ* (ppm) = 7.39 (d, *^3^J*(H-H) = 2.1 Hz, 2H, *H*-3), 6.85 (d, *^3^J*(H-H) = 2.1 Hz, 2H, *H*-5), 4.19 (s, 4H, N-*H*, br).

**^13^C{^1^H} NMR (126 MHz, CDCl_3_):** *δ* (ppm) = 143.3 (*C*-2), 135.9 (*C*-4), 130.5 (*C*-3), 120.3 (*C*-5), 110.0 (*C*-6), 108.8 (*C*-1).

**HRMS** Calculated m/z for [C_12_H_8_N_2_OBr_4_H]^+^: 512.7443, found: 512.7446.

**Synthesis of S6:**

****To a two-neck flask, acetic anhydride (40 mL) was added and cooled down to 0 °C with an ice bath. Into the flask, formic acid (20 mL) was added dropwise, and the mixture was degassed for 15 minutes. Then, the reaction flask was equipped with a reflux condenser, and the reaction mixture was heated to 50 °C. After 2 h of stirring at 50 °C, the reaction mixture was cooled to 0 °C, and **S5** (1.0 g, 1.9 mmol) was added portion-wise. The reaction mixture was then stirred for 16 hours at room temperature.

The reaction mixture was added to ice-cold water and filtered. The obtained solid was dried under reduced pressure. Yield: 841 mg (86 %).

**^1^H NMR (400 MHz, DMSO):** *δ* (ppm) = 9.90 (d, *J* = 20.3 Hz, 1.6H), 9.58 (d, *J* = 11.2 Hz, 0.4H), 9.29 (s, 0.2H), 8.19 (d, *J* = 19.2 Hz, 2H), 7.82 (d, *J* = 18.4 Hz, 1.9H), 7.38 (s, 0.4H), 7.13 (d, *J* = 25.6 Hz, 1.6H).

The NMR assignments were not performed due to the observation of several rotamers (formamide) in the solution.

**HRMS** Calculated m/z for [C_14_H_8_N_2_O_3_Br_4_+H]^+^: 568.7341, found: 568.7344.

**Synthesis of S7:**

^^To a two-neck flask equipped with a reflux condenser, formamide **S6** (700 mg, 1.22 mmol), (3,5-di-*tert*-butylphenyl)boronic acid (2.51 g, 10.4 mmol, 8.5 equiv), and K_3_PO_4_ (3.64 g, 17.1 mmol, 14.0 equiv) were added and suspended in a mixture of tetrahydrofuran and water (THF:H_2_O, 24:30 mL). The resulting suspension was degassed for 15 minutes, after which PdXPhosG3 (165.8 mg, 0.20 mmol, 0.16 equiv) was added, and the mixture was further degassed for an additional 5 minutes. The reaction mixture was then heated to reflux and stirred overnight.

After cooling to room temperature, 50 mL of ethyl acetate and 50 mL of distilled water were added. The organic layer was separated and washed successively with 1 M NaOH solution and brine. The combined organic extracts were dried over anhydrous sodium sulfate (Na_2_SO_4_), filtered, and concentrated under reduced pressure. The crude solid was purified by silica gel column chromatography using a mixture of ethyl acetate and petroleum ether. Yield: 660 mg (52 %).

**^1^H NMR (400 MHz, CDCl_3_):** *δ* (ppm) = 8.56 (d, *J* = 10.9 Hz, 0.6H), 8.36 (d, *J* = 9.9 Hz, 1H), 8.17 – 8.06 (m, 0.5H), 8.00 (d, *J* = 10.8 Hz, 0.6H), 7.77 (s, 0.3H), 7.54 – 7.26 (m, 14.4H), 7.23 – 7.14 (m, 1.2H), 7.04 – 6.96 (m, 1.2H), 6.83 (s, 0.5H), 1.41 – 1.25 (m, 72H).

The NMR assignments were not performed due to the observation of several rotamers (formamide) in the solution.

**HRMS** Calculated m/z for [C_70_H_92_N_2_O_3_+H]^+^: 1009.7181, found: 1009.7175.

**Synthesis of L*^t^*^Bu2^:**

A 100 mL Schlenk flask was charged with formamide **S7** (350 mg, 0.35 mmol, 1.0 equiv) and 25 mL of dry CH_2_Cl_2_. Diisopropylamine (178.9 mg, 1.8 mmol, 5.1 equiv) was then added to the solution under an inert atmosphere. The reaction mixture was cooled to 0 °C, and POCl_3_ (271.1 mg, 1.8 mmol, 5.1 equiv) was added dropwise under the same inert conditions. After stirring at room temperature for 4 hours, 15 mL of 1.5 M Na_2_CO_3_ solution was added, and the mixture was stirred overnight.

The reaction was quenched by adding 25 mL of CH_2_Cl_2_ and 25 mL of distilled water. The organic layer was separated and subsequently washed with 25 mL of saturated NaHCO_3_ solution, followed by a brine wash. The organic phase was dried over anhydrous Na_2_SO_4_ and filtered. The crude solid was purified by column chromatography using ethyl acetate and petroleum ether. Yield: 233 mg (69 %).

**^1^H NMR (500 MHz, CDCl_3_):** *δ* (ppm) = 7.56 (t, *J* = 1.83 Hz, 2H, *H*-10), 7.52 – 7.45 (m, 8H, *H*-16, *H*-8 and *H*-5), 7.35 (d, *J* = 1.8 Hz, 4H, *H*-14), 7.24 (d, *J* = 1.8 Hz, 2H, *H*-3), 1.42 (s, 36H, *H*-12), 1.35 (s, 36H, *H*-18).

**^13^C{^1^H} NMR (126 MHz, CDCl_3_):** *δ* (ppm) = 172.5 (N-*C*), 152.4 (*C*-6), 151.8 (*C*-15), 151.2 (*C*-9), 144.9 (*C*-2), 142.4 (*C*-4), 139.0 (*C*-7), 135.9 (*C*-13), 125.0 (*C*-5), 123.7 (*C*-8), 122.9 (*C*-10 or *C*-16), 122.8 (*C*-16 or *C-*10), 121.9 (*C*-14), 116.5 (*C*-3), 116.1 (*C*-1), 35.3 (*C*-11), 35.1 (*C*-17), 31.7 (*C*-12), 31.6 (*C*-18).

**HRMS** Calculated m/z for [C_70_H_88_N_2_O+H]^+^: 973.6969, found: 973.6972.

**EA**: Anal. Calc. for [C_70_H_88_N_2_O] [0.2 EtOAC]: C 85.80; H 9.11; N 2.83; Found: C 85.88; H 9.00; N 2.74.

**FTIR:** ν (cm^-1^) 2959, 2905, 2867, 2360, 2335, 2117 (ν_C≡N_), 1594, 1560, 1520, 1476, 1462, 1421, 1391, 1362, 1306, 1275, 1247, 1205, 1147, 1081, 1008, 938, 899, 869, 837, 819, 778, 738, 713, 671, 615, 582, 547, 425, 409.

### **Synthesis and characterization of heteroleptic rhodium(I) complex [Het-Rh_2_]*^t^*^Bu2^**

Scheme S7: Synthesis of **[Het-Rh_2_]^tBu2^**.

An oven-dried Schlenk tube was charged with ligand **L*^t^*^Bu2^** (35 mg, 0.04 mmol, 1.0 eq), bis(diphenylphosphino)methane (13.8 mg, 0.04 mmol, 1.0 eq), and [Rh(cod)₂]BF₄ (14.6 mg, 0.04 mmol, 1.0 eq), then evacuated before the addition of 5 mL dichloromethane. The reaction mixture was stirred overnight, after which the volatiles were removed under reduced pressure. The resulting crude solid was purified by crystallization from THF and diethyl ether. Yield: 10 mg (35 %).

**^1^H{^31^P} NMR (600 MHz, CD_3_CN):** *δ* (ppm) = 7.65 (t, *J* = 1.8 Hz, 4H, *H*-10), 7.52 (t, *J* = 1.8 Hz, 4H, *H*-16), 7.49 (d, *J* = 1.9 Hz, 4H, *H*-3), 7.37 – 7.34 (m, 24H, *H*-14 and *H*-21), 7.32 (d, *J* = 1.9 Hz, 4H, *H*-5), 7.15 (d, *J* = 1.7 Hz, 8H, *H*-8), 7.08 – 7.02 (m, 8H, *H*-23), 6.79 (t, *J* = 7.9 Hz, 16H, *H*-22), 4.11 (s, 4H, *H*-19), 1.34 (s, 72H, *H*-12), 1.26 (s, 72H, *H*-18).

**^31^P{^1^H} NMR (243 MHz, CD_3_CN):** *δ* (ppm) =18.9-18.2 (m).

**^13^C{^1^H} NMR (151 MHz, CD_3_CN):** *δ* (ppm) = 152.9 (*C*-15), 152.4 (*C*-9), 152.2 (*C*-6)**,** 144.4 (*C*-2), 141.7 (*C*-4)**,** 138.8 (*C*-7)**,** 137.0 (*C*-13)**,** 134.2 (*C*-20), 132.9 (*C*-21), 131.7 (*C*-23), 129.5 (*C*-22), 126.7 (*C*-5)**,** 124.7 (*C*-10)**,** 124.5 (*C*-16)**,** 124.4 (*C*-8)**,** 122.4 (*C*-14)**,** 117.1 (*C*-3)**,** 116.1 (*C*-1), 35.9 (*C*-11), 35.7 (*C*-17), 32.1 (*C*-12), 31.6 (*C*-18). C-19 and isocyanide carbon signal could not be detected due to coupling with distinct ^31^P and ^103^Rh nuclei.

**EA**: Anal. Calc. for C_190_H_220_N_4_O_2_P_4_Rh_2_B_2_F_8_·2·C_4_H_8_O: C 73.41; H 7.34; N 1.73; Found: C 73.06; H 7.20; N 1.81.

**HRMS** Calculated m/z for [C_190_H_220_N_4_O_2_P_4_Rh_2_]^2+^: 1459.7143, found: 1459.7160.

**FTIR:** ν (cm^-1^) 2955, 2905, 2867, 2362, 2328, 2084 (ν_C≡N_), 1592, 1559, 1476, 1436, 1422, 1391, 1362, 1308, 1274, 1247, 1202, 1149, 1121, 1056, 997, 930, 898, 870, 780, 738, 713, 692, 671, 626, 545, 504.

### **Synthesis and characterization of bridging diisocyanide ligand L^Mes^**

Scheme S8: Synthesis of ligand **L^Mes^**.

**Synthesis of S8:**

**S8** was synthesized by a modified literature procedure.^[1]^ 1-Bromo-3,5-diiodobenzene (3.0 g, 7.3 mmol, 1.0 equiv), mesityl boronic acid (2.41 g, 14.7 mmol, 2 equiv),, sodium carbonate (4.67 g, 44.0 mmol, 6 equiv), were charged in a two-necked 500 ml flask and suspended in a toluene/ethanol/water mixture (160/80/80 mL). The suspension was degassed for 30 min prior to the addition of the catalyst. Pd(PPh_3_)_4_ (508.8 mg, 0.44 mmol, 0.06 equiv), was added, and the reaction mixture was purged with N_2_ for an additional 15 min. The reaction mixture was stirred and refluxed at 95 °C. After 16 h, the reaction was quenched by the addition of 75 mL of CH_2_Cl_2_ and 75 mL of distilled water. The organic layer was collected, washed with brine and subsequently dried over anhydrous Na_2_SO_4_. The crude product was purified by column chromatography using a mixture of CH_2_Cl_2_ and n-hexane as the eluent. Yield: 2.19 g (76 %)

**^1^H NMR (400 MHz, CDCl_3_):** *δ* (ppm) = 7.27 (s), 6.93 (s), 6.86 (s), 2.32 (s), 2.05 (s).

**Synthesis of S9:**

**S9** was synthesized by a modified literature procedure.^[2]^ To a microwave vial **S8** (1.0 g, 2.54 mmol, 1.0 equiv), potassium acetate (748.5 mg, 7.63 mmol, 3.0 equiv), and bis(pinacolato)diboron (774.7 mg, 3.05 mmol, 1.2 equiv) were added, and the flask was evacuated for 3 cycles and backfilled with N_2_. Subsequently, 22 mL of dry 1,4-dioxane was added, and the reaction mixture was degassed with N_2_ for 30 min. Pd(dppf)Cl_2_·CH_2_Cl_2_ (81.4 mg, 0.1 mmol, 0.04 equiv) was then added as a catalyst, followed by additional degassing for 10 min. The reaction mixture was heated at 80 °C for 2 days. After cooling to room temperature, a saturated aqueous NH₄Cl solution was added, and the mixture was extracted twice with ethyl acetate. The combined organic layers were dried over anhydrous Na_2_SO_4_ and filtered. The filtrate was passed through a short pad of silica gel, concentrated under reduced pressure, washed with cold methanol, and finally dried in vacuo to afford compound S9. Yield: 755 mg (67 %).

**^1^H NMR (400 MHz, CDCl_3_):** *δ* (ppm) = 7.53 (d, *J* = 2.0 Hz, 2H), 7.04 (t, *J* = 2.0 Hz, 1H), 6.91 (s, 4H), 2.31 (s, 6H), 2.04 (s, 12H), 1.34 (s, 12H).

**Synthesis of S10:**

**S1** (140 mg, 0.34 mmol, 1.0 equiv), potassium carbonate (280.4 mg, 2.03 mmol, 6.0 equiv) and **S9** (460.6 mg, 1.01 mmol, 3.0 equiv) were added to a microwave vial and suspended in a mixture of tetrahydrofuran and water (THF:H_2_O, 15:5 mL). The resulting suspension was degassed for 15 minutes, after which PdXPhosG3(22.9 mg, 0.03 mmol, 0.08 equiv) was added, and the mixture was further degassed for an additional 5 minutes. The reaction mixture was then heated to 80 °C and stirred for 3 h.

After cooling to room temperature, 25 mL of ethyl acetate and 25 mL of distilled water were added. The organic layer was separated and washed successively with 1 M NaOH solution and brine. The combined organic extracts were dried over anhydrous Na_2_SO_4_, filtered, and concentrated under reduced pressure. The crude solid was purified by silica gel column chromatography using a mixture of ethyl acetate and petroleum ether. Yield: 122 mg (41 %).

**^1^H NMR (400 MHz, CDCl_3_):** *δ* (ppm) = 8.83 (dd, *J* = 11.4, 7.5 Hz, 0.56H), 8.49 – 8.44 (m, 2.22H), 8.33 – 8.27 (m, 1.51H), 8.11 (s, 1.16H), 8.00 (s, 0.31H), 7.46-7.39 (m, 3.12H), 7.23 – 7.19 (m, 4.87H), 7.14 – 7.11 (m, 1.77H), 6.93 – 6.87 (m, 10.18H), 2.32 (s, 12H), 2.02 (s, 24H).

The NMR assignments were not performed due to the observation of several rotamers (formamide) in the solution.

**HRMS** Calculated m/z for [C_62_H_60_N_2_O_3_+K]^+^: 919.4236; found: 919.4224.

**Synthesis of L^Mes^:**

A 100 mL Schlenk flask was charged with formamide **S10** (100 mg, 0.11 mmol, 1.0 equiv) and 15 mL of dry CH_2_Cl_2_. Diisopropylamine (58.6 mg, 0.56 mmol, 5.1 equiv) was then added to the solution under an inert atmosphere. The reaction mixture was cooled to 0 °C, and POCl_3_ (88.7 mg, 0.56 mmol, 5.1 equiv) was added dropwise under the same inert conditions. After stirring at room temperature for 4 hours, 7 mL of 1.5 M Na_2_CO_3_ solution was added, and the mixture was stirred overnight.

The reaction was quenched by adding 25 mL of CH_2_Cl_2_ and 25 mL of distilled water. The organic layer was separated and subsequently washed with 25 mL of saturated NaHCO_3_ solution, followed by a brine wash. The organic phase was dried over anhydrous sodium sulfate (Na_2_SO_4_) and filtered. The crude solid was purified by column chromatography using ethyl acetate and petroleum ether. Yield: 58 mg (60 %).

**^1^H NMR (500 MHz, CDCl_3_):** *δ* (ppm) = 7.54 (d, *J* = 8.3 Hz, 2H, *H*-2), 7.47 (dd, *J* = 8.3, 2.0 Hz, 2H, *H*-3), 7.27 (d, *J* = 1.6 Hz, 4H, *H*-8), 7.24 (d, *J* = 2.0 Hz, 2H, *H*-5), 6.96 (t, *J* = 1.5 Hz, 2H, *H*-10), 6.94 (s, 8H, *H*-13), 2.33 (s, 12H, *H*-15), 2.04 (s, 24H, *H*-16).

**^13^C{^1^H} NMR (126 MHz, CDCl_3_):** *δ* (ppm) = 170.3 (N*C*), 151.6 (*C*-6), 144.0 (*C*-4), 142.3 (*C*-9), 138.7 (*C*-7), 138.3 (*C*-11), 137.1 (*C*-14), 135.9 (*C*-12), 131.0 (*C*-10), 128.8 (*C*-2), 128.4 (*C*-13), 126.3 (*C*-8), 123.7 (*C*-3), 117.8 (*C*-1), 117.5 (*C*-5), 21.2 (*C*-15), 21.0 (*C*-16).

**HRMS** Calculated m/z for [C_62_H_56_N_2_O+NH_4_]^+^: 862.4731; found: 862.4742.

**FTIR:** ν (cm^-1^) 2949, 2917, 2856, 2120 (ν_C≡N_), 1611, 1597, 1566, 1501 1485, 1440, 1425, 1389, 1263, 1228, 1181, 1124, 1078, 1028, 962, 879, 850, 826, 791, 747, 721, 569.

### **Synthesis and characterization of homoleptic rhodium(I) complex [Hom-Rh_2_]^Mes^**

Scheme S9: Synthesis of rhodium(I) complex **[Hom-Rh_2_]^Mes^**.

An oven-dried Schlenk tube was charged with ligand **L^Mes^** (11.5 mg, 0.014 mmol, 2.0 eq) and [Rh(cod)_2_]BF_4_ (2.8 mg, 0.007 mmol, 1.0 eq), then evacuated before the addition of 1 mL of dichloromethane. The reaction mixture was stirred overnight, after which the volatiles were removed under reduced pressure. The resulting crude solid was purified by crystallization from dichloromethane and n-hexane. Single crystals suitable for X-ray diffraction were obtained by layering n-hexane on a concentrated dichloromethane solution of **[Hom-Rh_2_]^Mes^**. Yield: 12 mg (47 %).

**^1^H NMR (500 MHz, CD_2_Cl_2_):** *δ* (ppm) = 7.62 (d, *J* = 1.8 Hz, 8H, *H*-5), 7.17 (d, *J* = 1.5 Hz, 16H, *H*-8), 7.04 (dd, *J* = 8.4, 1.8 Hz, 8H, *H*-3), 6.99 (d, *J* = 8.4 Hz, 8H, *H*-2), 6.95 (t, *J* = 1.5 Hz, 8H, *H*-10), 6.89 (s, 32H, *H*-13)2.30 (s, 48H, *H*-15), 1.95 (s, 48H, *H*-16), 1.89 (s, 48H, *H*-16).

**^13^C{^1^H} NMR (126 MHz, CD_2_Cl_2_):** *δ* (ppm) = 153.7 (d, *J* = 53 Hz, N*C*, coupling due to ^103^Rh, I= 1/2), 151.2 (*C*-6), 145.6 (*C*-4), 142.9 (*C*-9), 138.6 (*C*-7), 138.2 (*C*-11), 137.4 (*C*-14), 135.8 (*C*-12), 132.0 (*C*-10), 128.6 (*C*-13), 127.2 (*C*-2), 126.5 (*C*-8), 123.8 (*C*-3), 117.0 (*C*-1), 116.3 (*C*-5), 21.1 (*C*-15), 21.0 (*C*-16).

**EA**: Anal. Calc. for [C_248_H_224_N_8_O_4_Rh_2_B_2_F_8_]·[1.5 CH_2_Cl_2_, 1.5 C_5_H_12_] (%): C 77.26; H 6.18; N 2.80; Found: C 77.18; H 6.55; N 2.41.

**HRMS** Calculated m/z for [C_248_H_224_N_8_O_4_Rh_2_]^2+^: 1791.7835, found: 1791.7785.

**FTIR:** ν (cm^-1^) 2948, 2917, 2854, 2361, 2334, 2143 (ν_C≡N_), 2009, 1700, 1611, 1595, 1571, 1521, 1485, 1440, 1424, 1380, 1263, 1229, 1184, 1124, 1055, 1029, 960.6, 938, 880, 850, 824, 790, 765, 726, 657, 584, 569, 539, 522, 452.

# **NMR spectra**


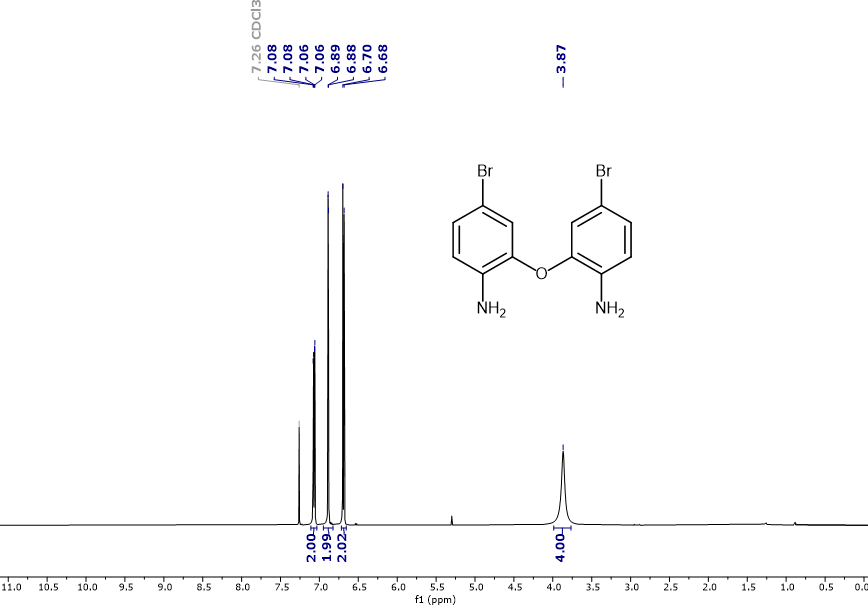


Figure S1: ^1^H NMR spectrum (500 MHz, 298 K) of **S1** in CDCl_3_.


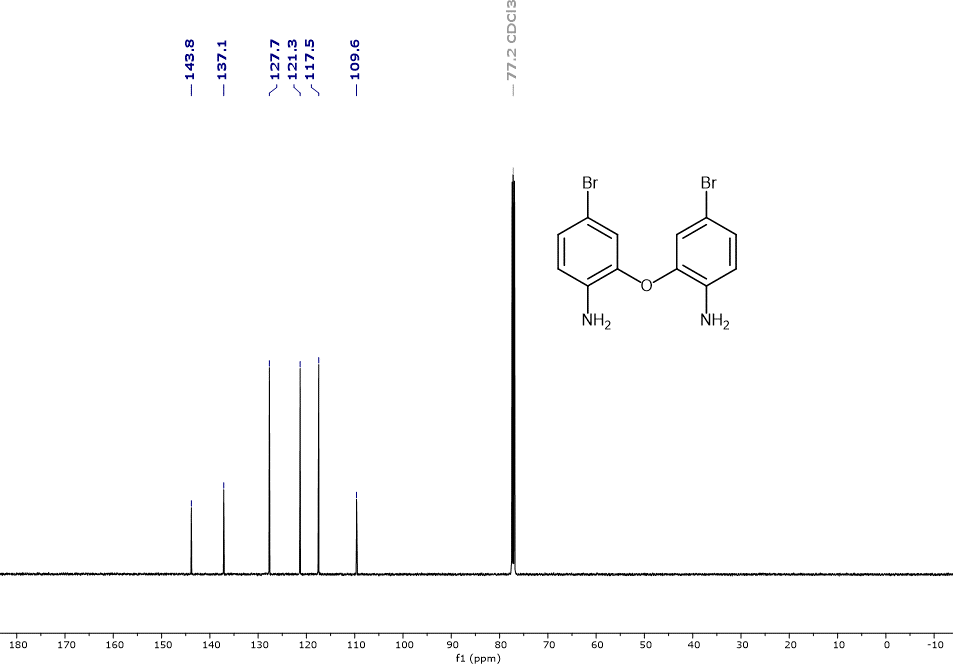


Figure S2: ^13^C{^1^H} NMR spectrum (126 MHz, 298 K) of **S1** in CDCl_3_.


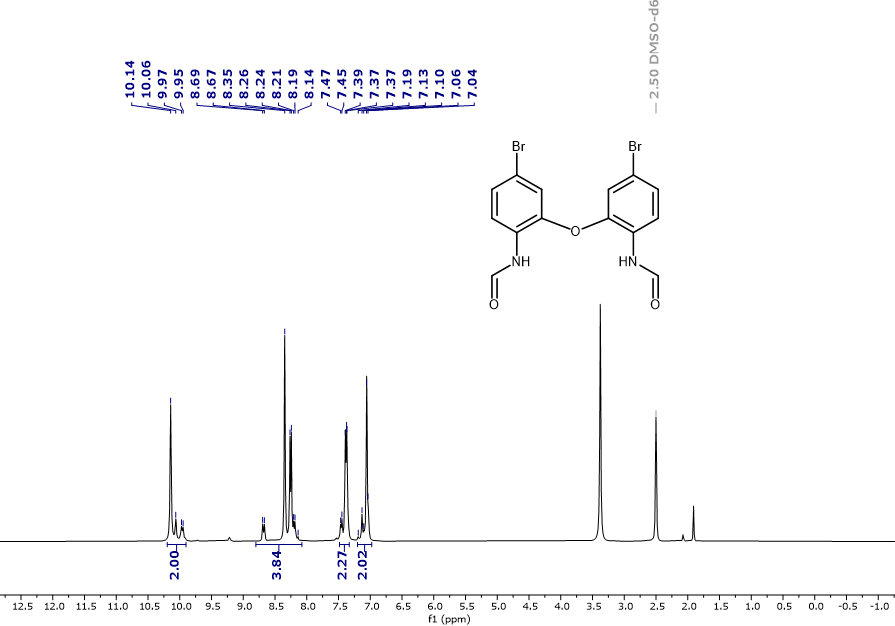


Figure S3: ^1^H NMR spectrum (400 MHz, 298 K) of **S2** in DMSO.


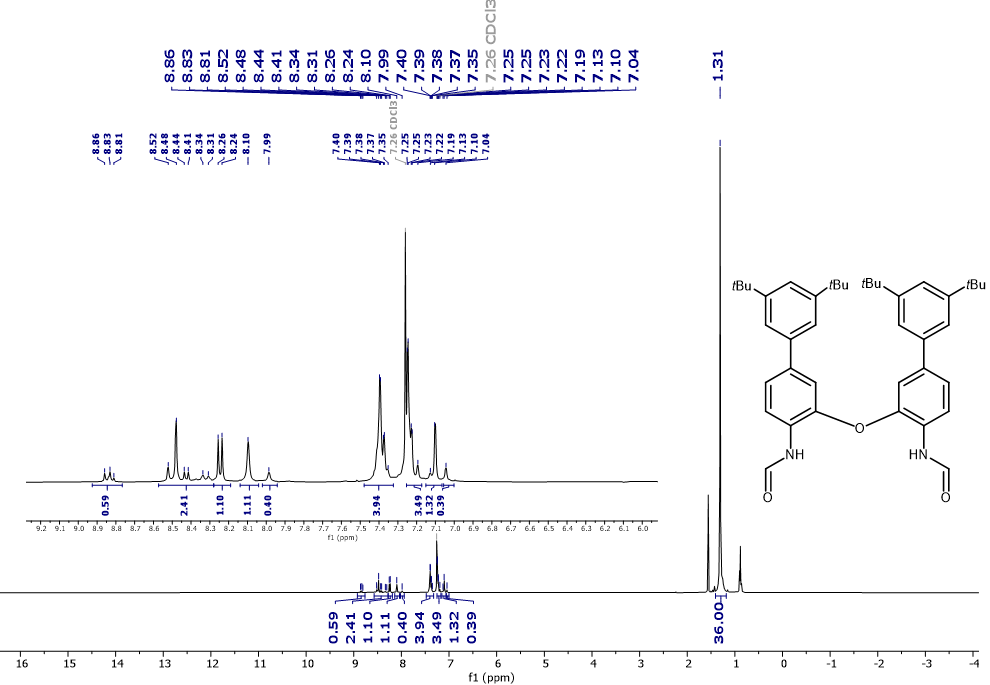


Figure S4: ^1^H NMR spectrum (400 MHz, 298 K) of **S3** in CDCl_3_.


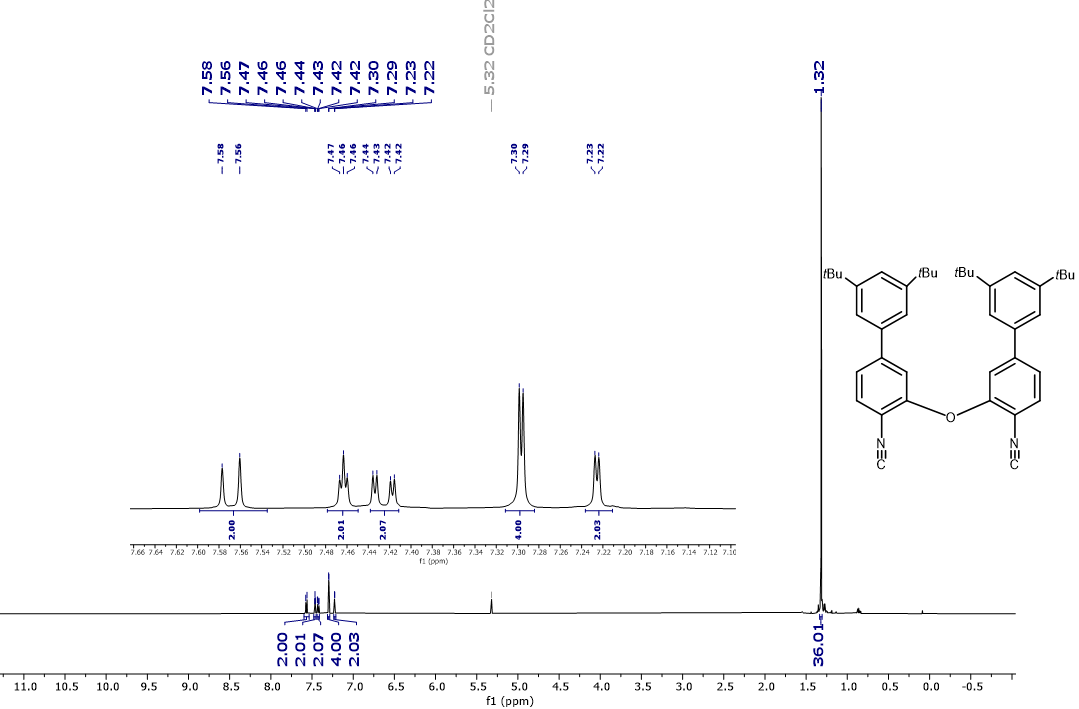


Figure S5: ^1^H NMR spectrum (500 MHz, 298 K) of **L^tBu^** in CD_2_Cl_2_.


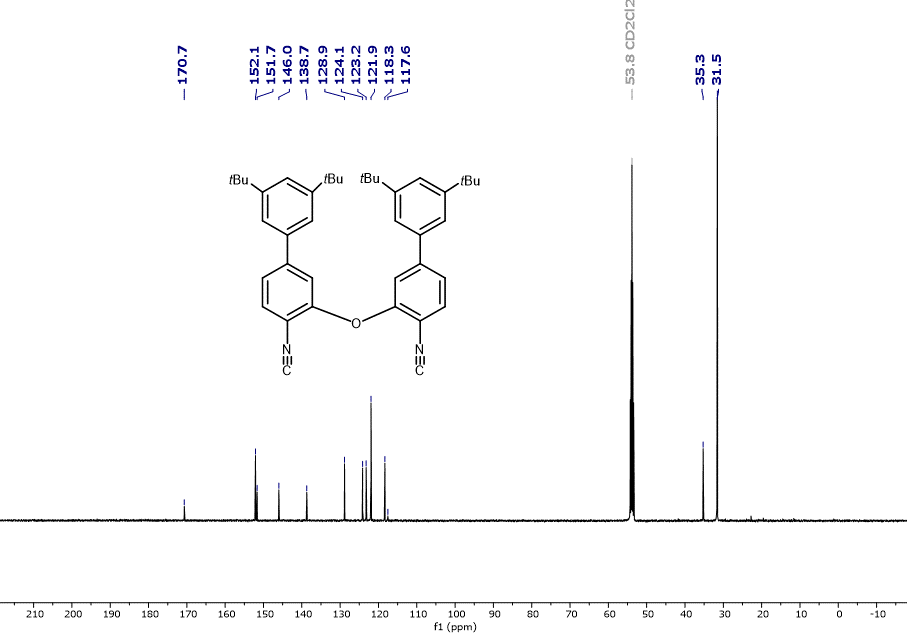


Figure S6: ^13^C{^1^H} NMR spectrum (126 MHz, 298 K) of **L^tBu^** in CD_2_Cl_2_.


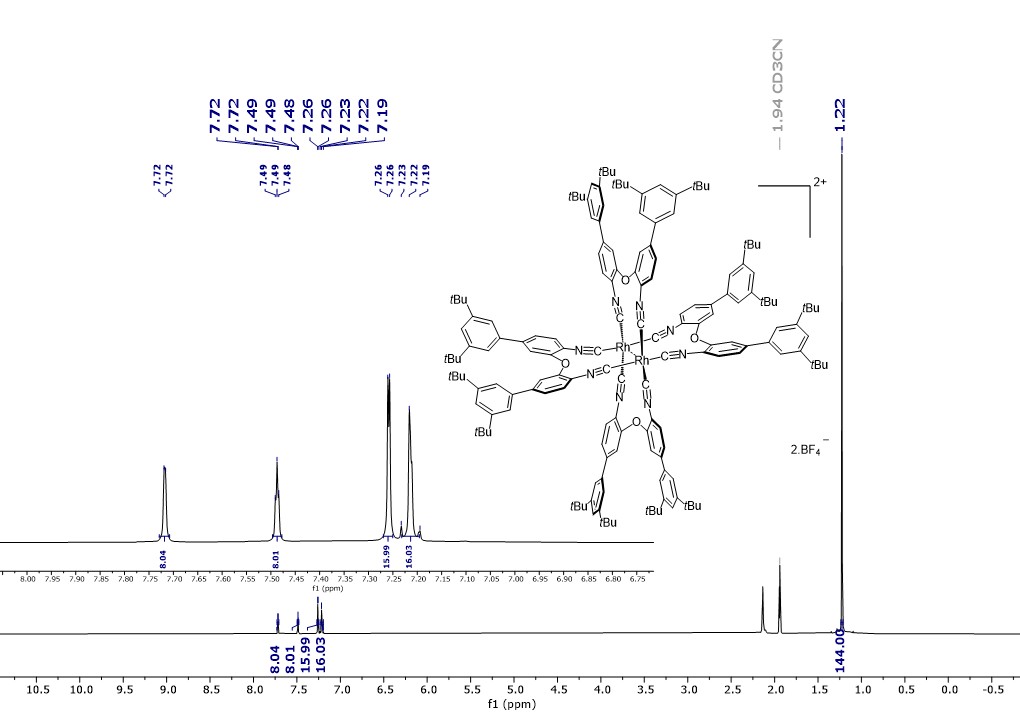


Figure S7: ^1^H NMR spectrum (500 MHz, 298 K) of **[Hom-Rh_2_]^tBu^** in CD_3_CN.


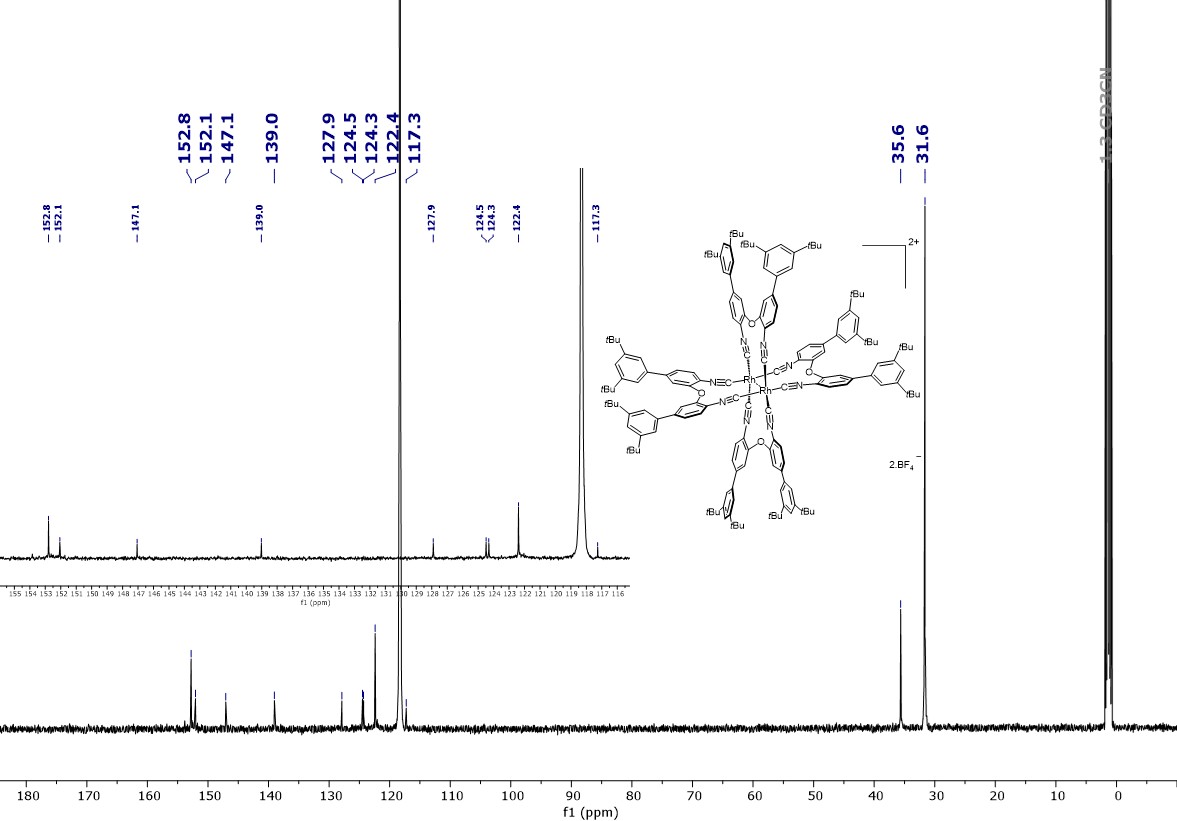


CD_3_CN

Figure S8: ^13^C{^1^H} NMR spectrum (126 MHz, 298 K) of **[Hom-Rh_2_]^tBu^** in CD_3_CN.


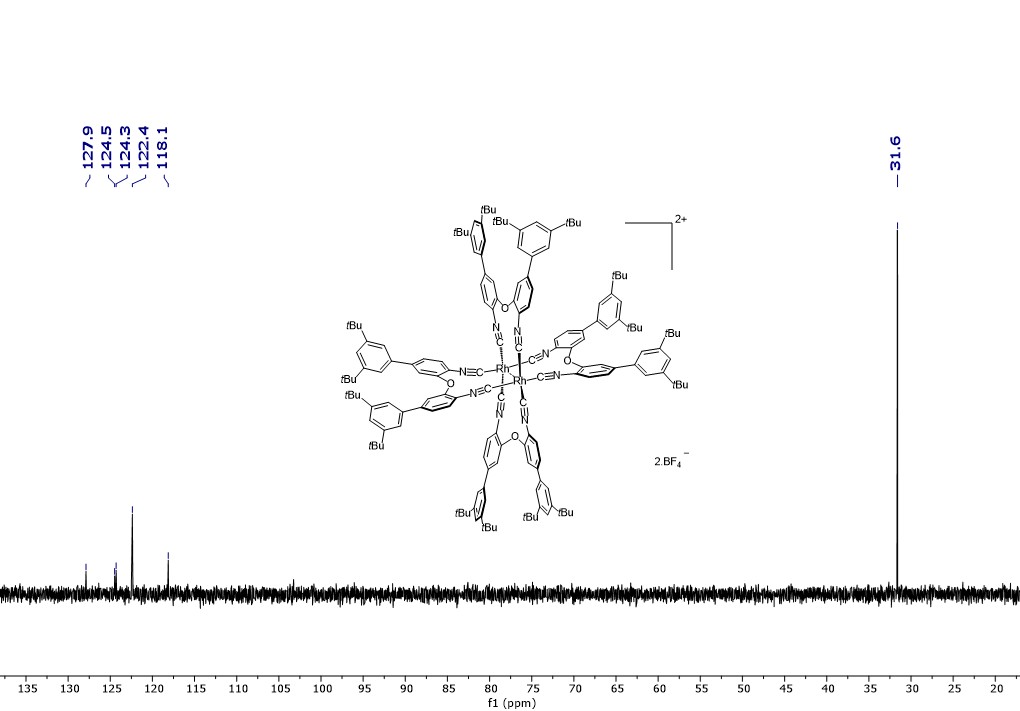


Figure S9: ^13^C DEPT-135 NMR spectrum (126 MHz, 298 K) of **[Hom-Rh_2_]^tBu^** in CD_3_CN.


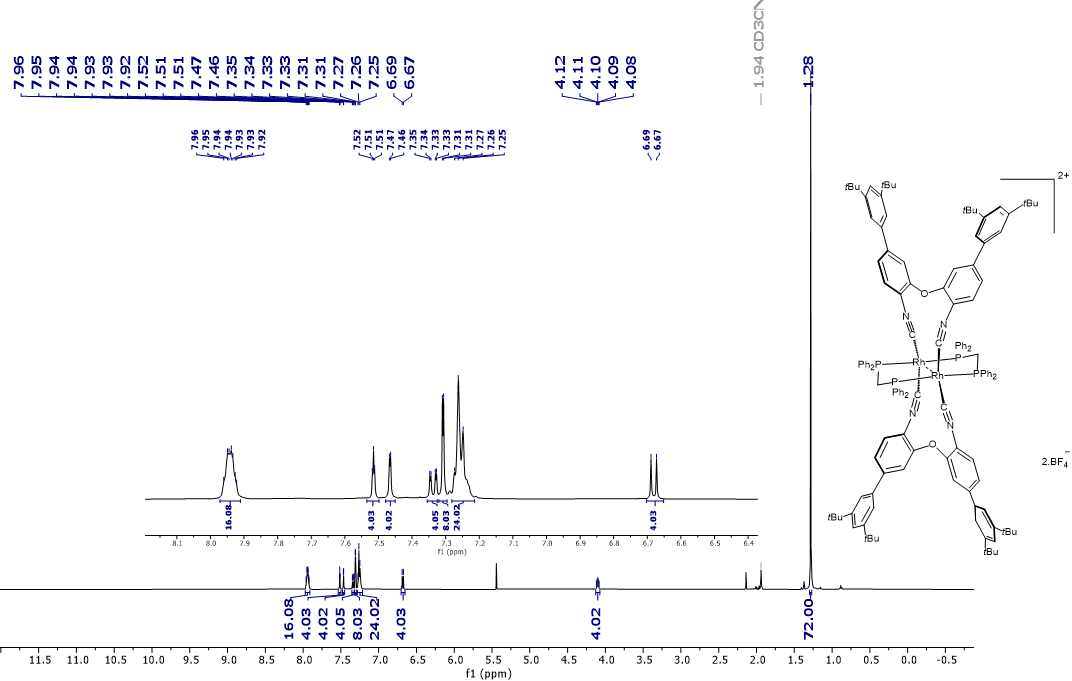


Figure S10: ^1^H NMR spectrum (500 MHz, 298 K) of **[Het-Rh_2_]^tBu^** in CD_3_CN.


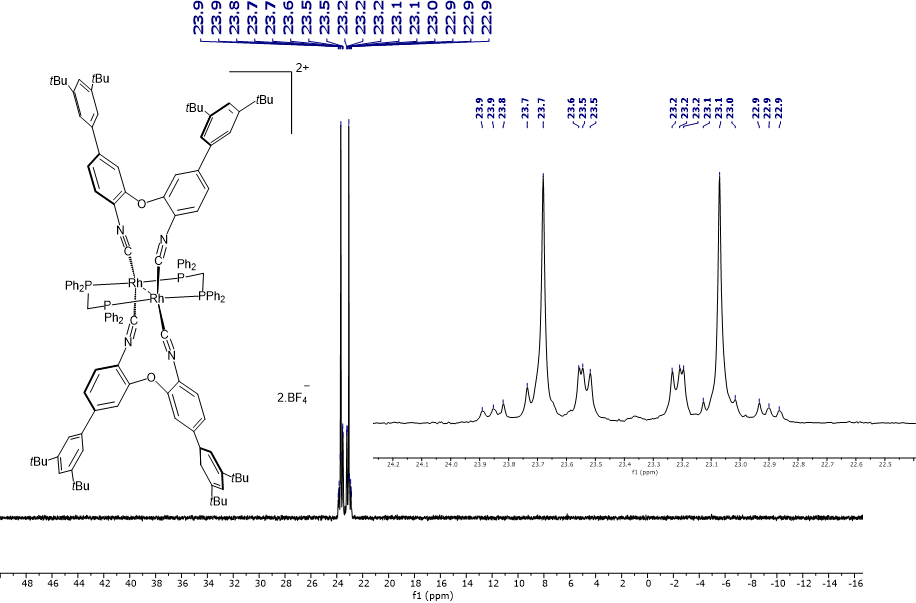


Figure S11: ^31^P{^1^H} NMR spectrum (202 MHz, 298 K) of **[Het-Rh_2_]^tBu^** in CD_3_CN.


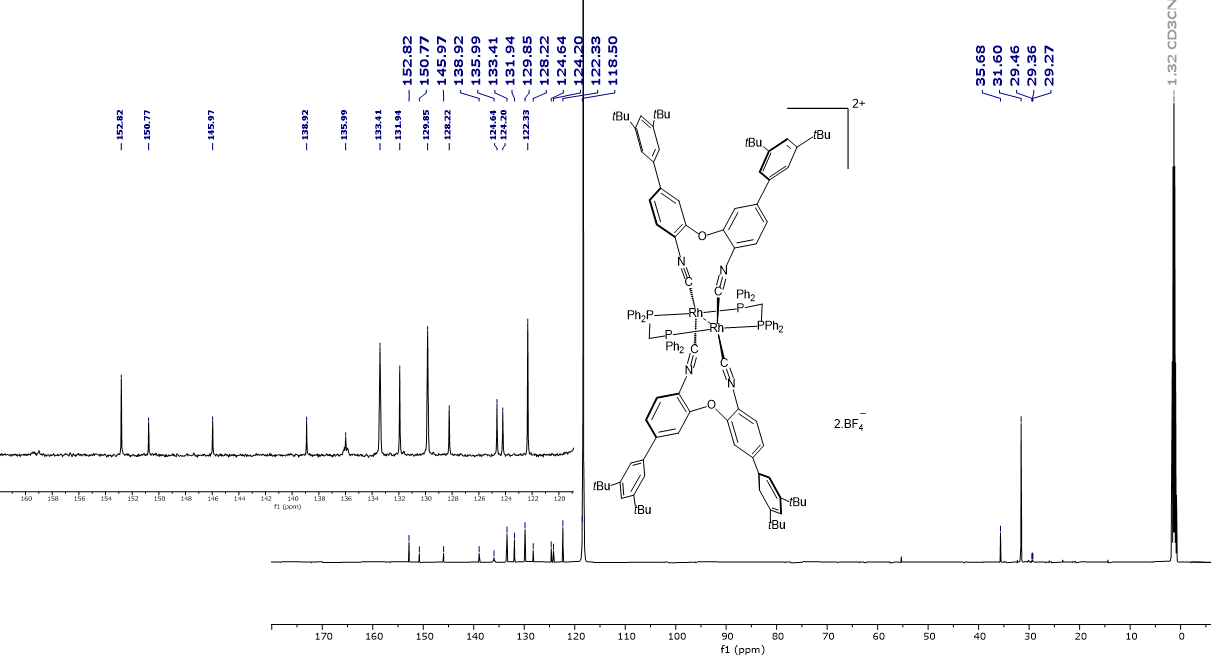


Figure S12: ^13^C{^1^H} NMR spectrum (126 MHz, 298 K) of **[Het-Rh_2_]^tBu^** in CD_3_CN.


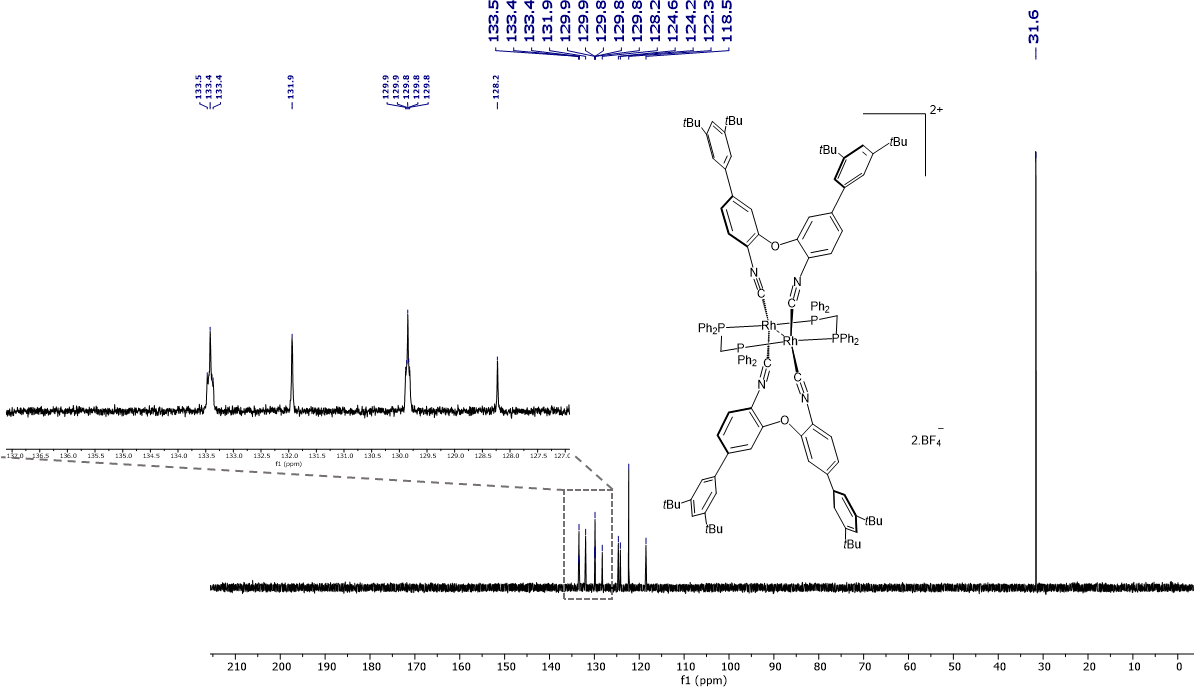


Figure S13: ^13^C DEPT-135 NMR spectrum (126 MHz, 298 K) of **[Het-Rh_2_]^tBu^** in CD_3_CN.


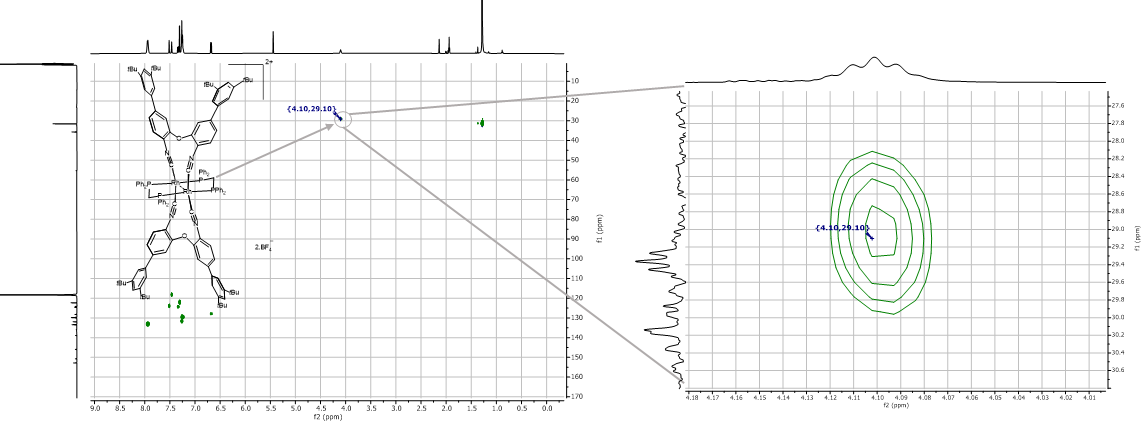


Figure S14: 2D ^1^H-^13^C HMQC spectrum of **[Het-Rh_2_]^tBu^** in CD_3_CN.


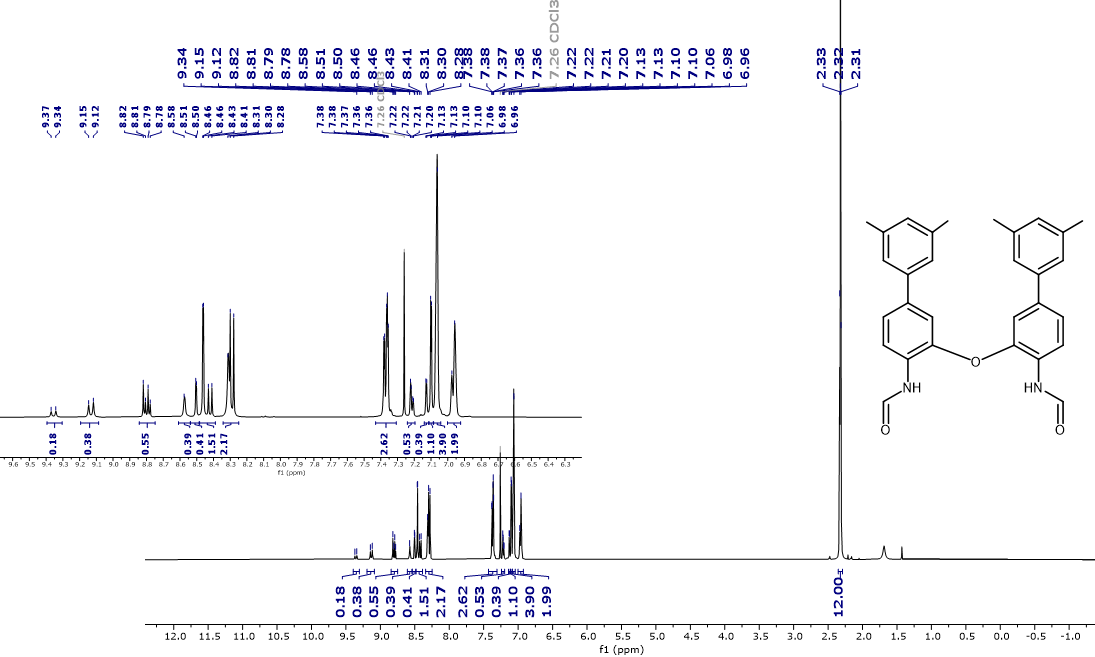


Figure S15: ^1^H NMR spectrum (400 MHz, 298 K) of **S4** in CDCl_3_.


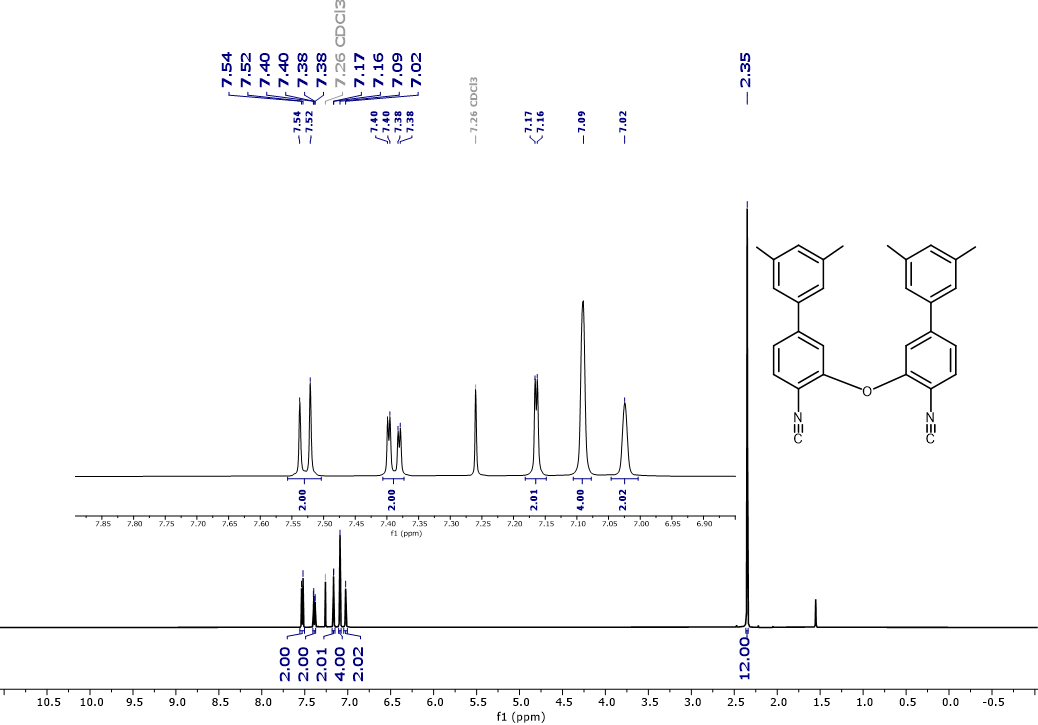


Figure S16: ^1^H NMR spectrum (500 MHz, 298 K) of **L^Me^** in CDCl_3_.


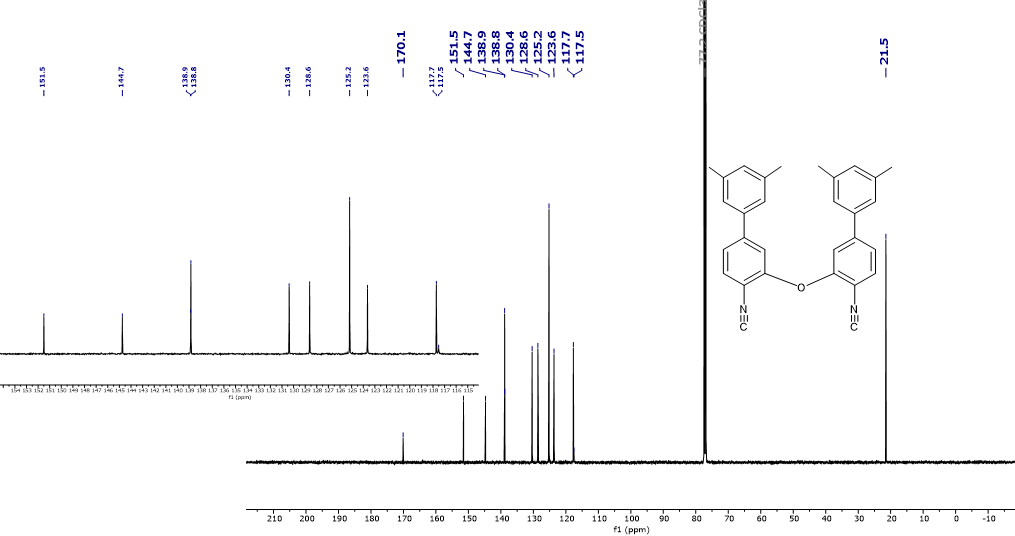


CDCl_3_

Figure S17: ^13^C{^1^H} NMR spectrum (126 MHz, 298 K) of **L^Me^** in CDCl_3_


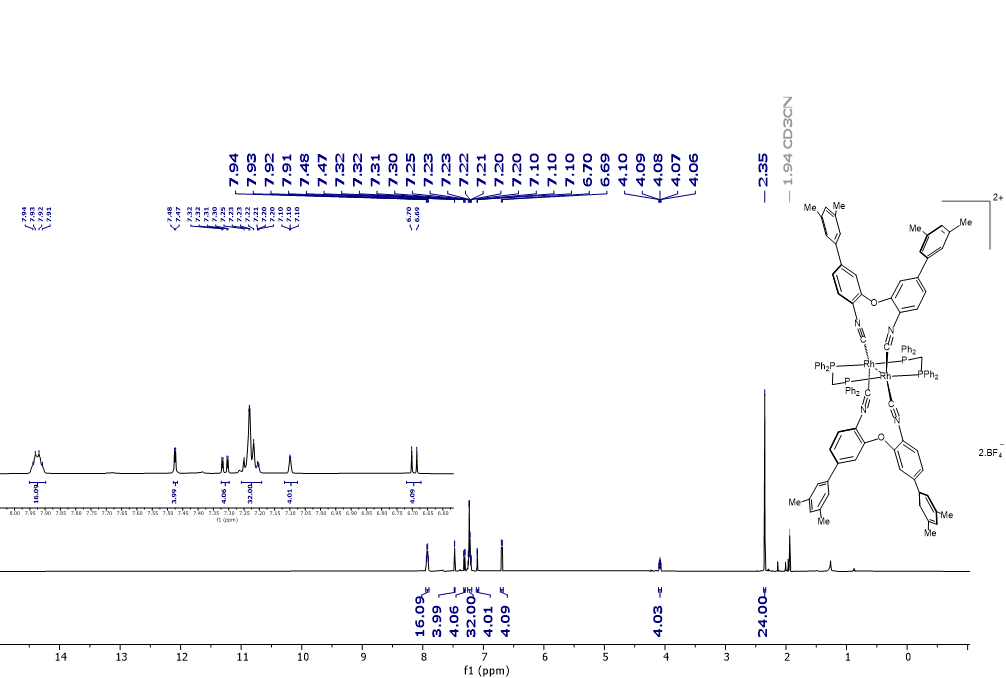


Figure S18: ^1^H NMR spectrum (500 MHz, 298 K) of **[Het-Rh_2_]^Me^** in CD_3_CN.


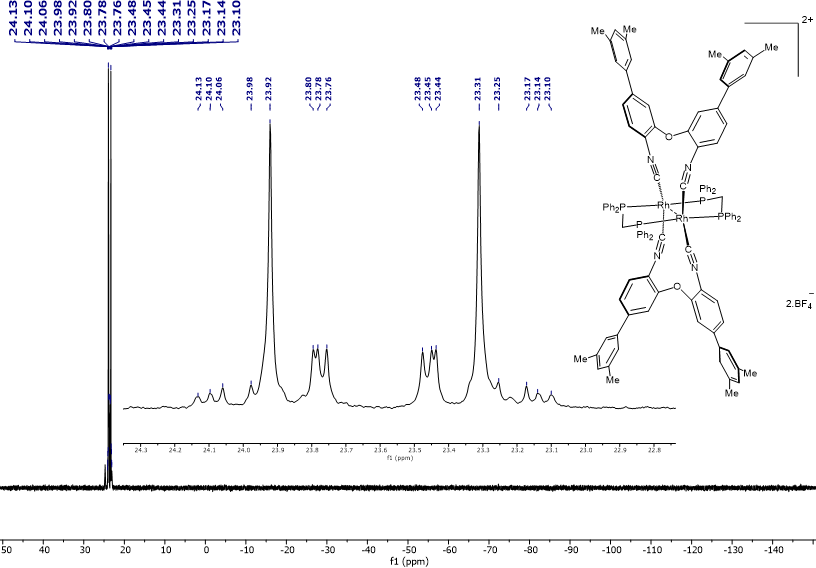


Figure S19: ^31^P{^1^H} NMR spectrum (202 MHz, 298 K) of **[Het-Rh_2_]^Me^** in CD_3_CN.


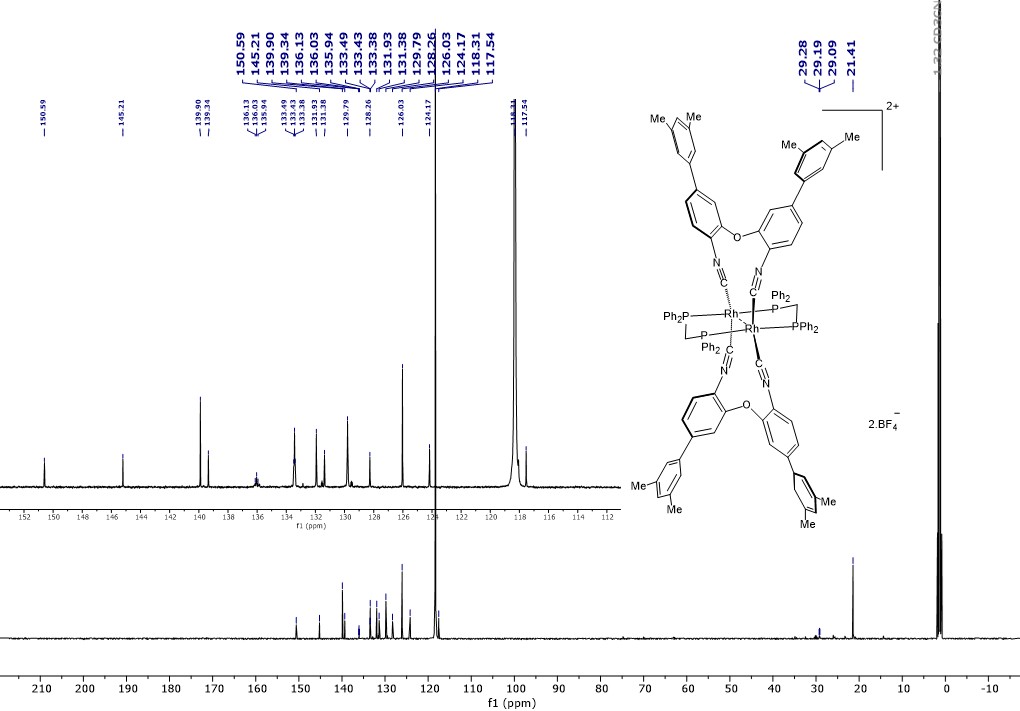


CD_3_CN

CD_3_CN

Figure S20: ^13^C{^1^H} NMR spectrum (126 MHz, 298 K) of **[Het-Rh_2_]^Me^** in CD_3_CN.


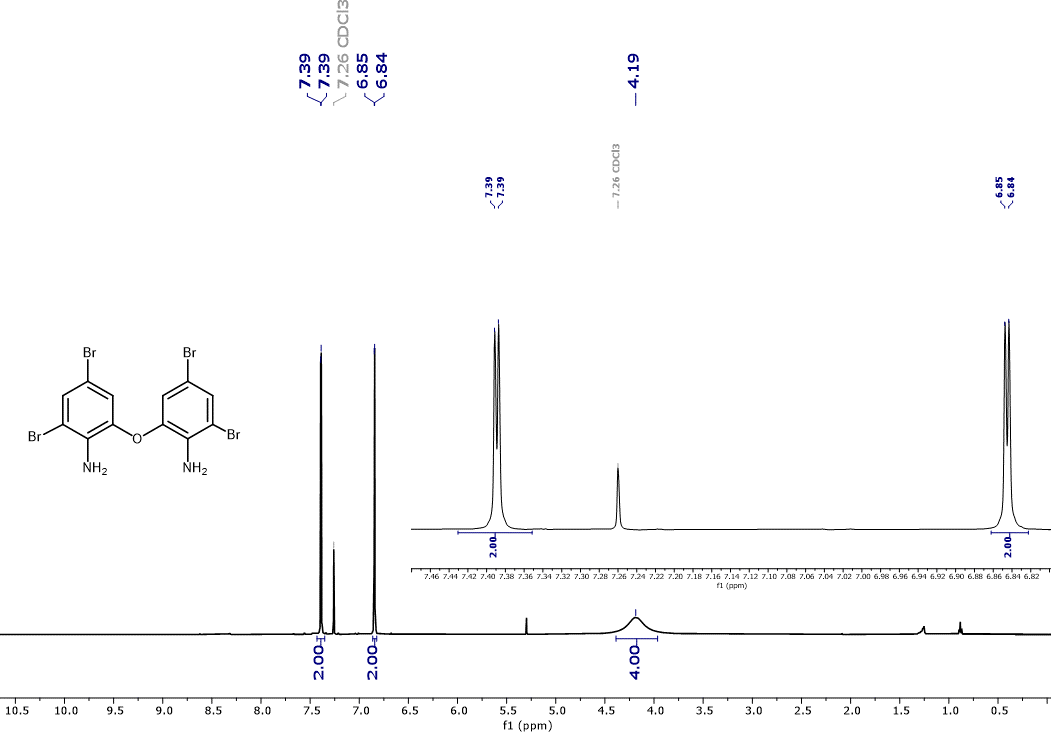


Figure S21: ^1^H NMR spectrum (500 MHz, 298 K) of **S5** in CDCl_3_.


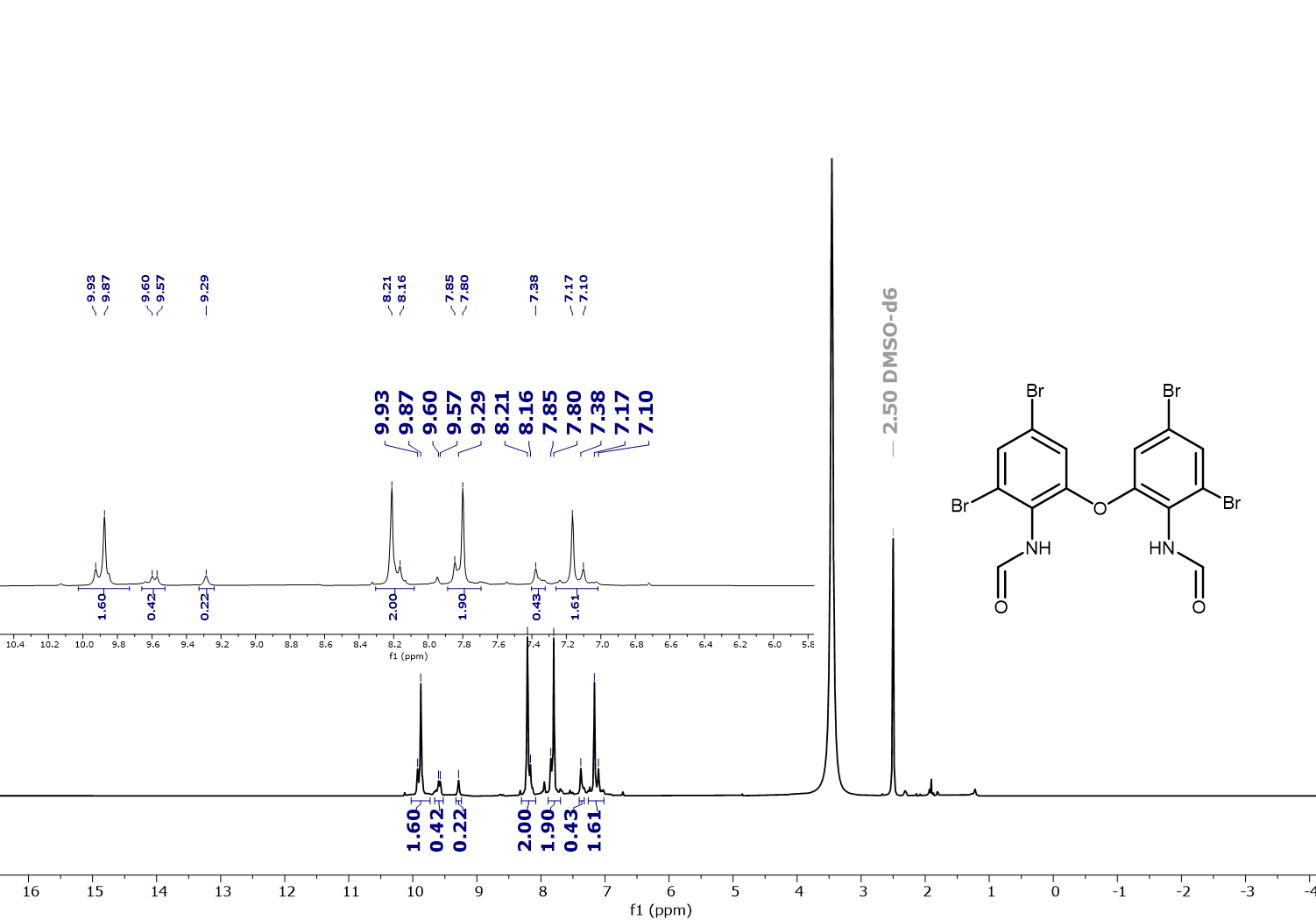


Figure S22: ^1^H NMR spectrum (400 MHz, 298 K) of **S6** in DMSO-d6.


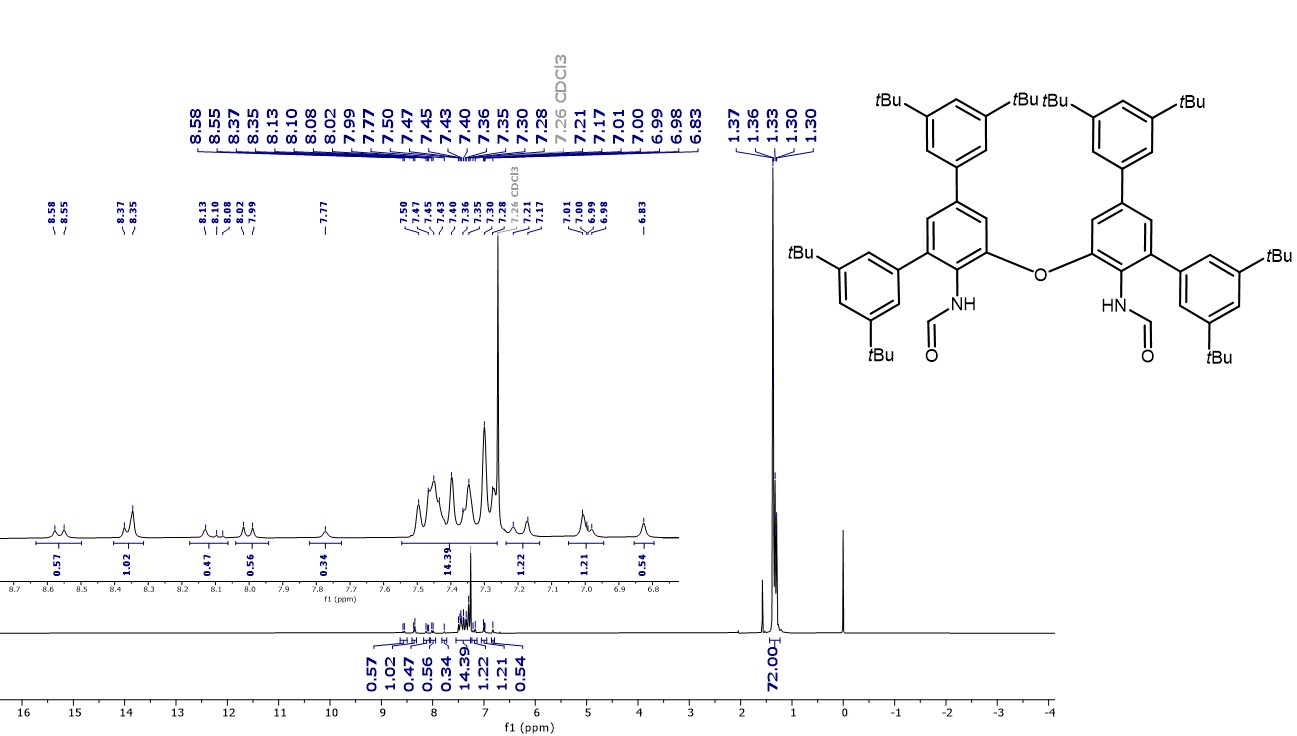


Figure S23: ^1^H NMR spectrum (400 MHz, 298 K) of **S7** in CDCl_3_.


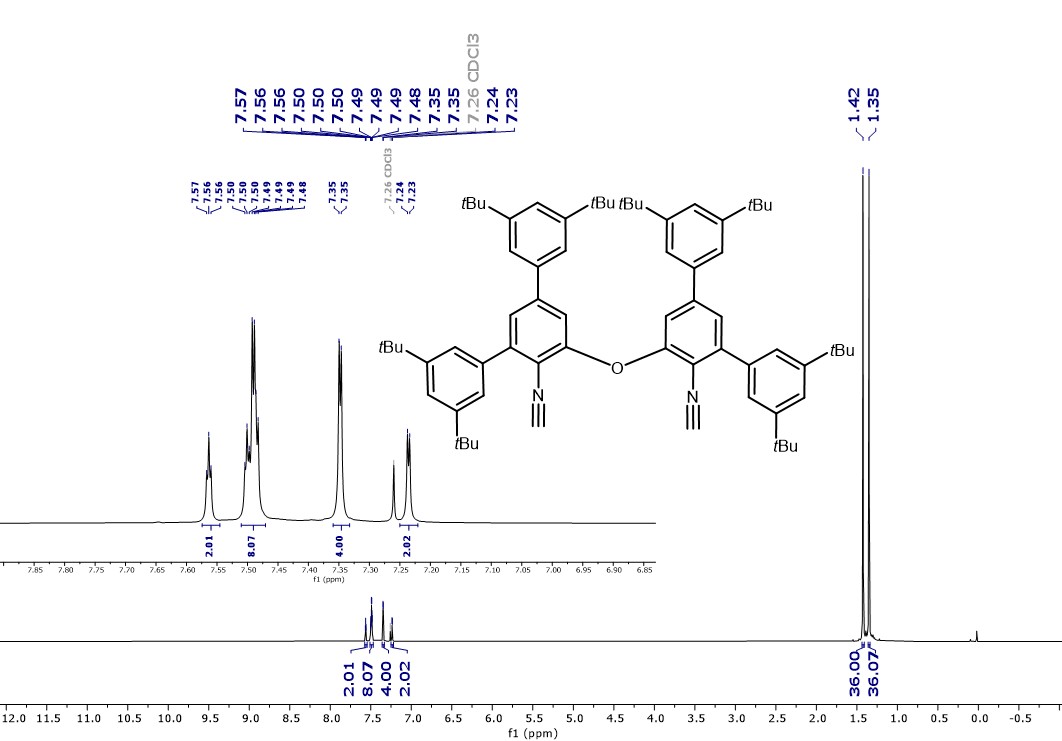


Figure S24: ^1^H NMR spectrum (500 MHz, 298 K) of **L^tBu2^** in CDCl_3_.


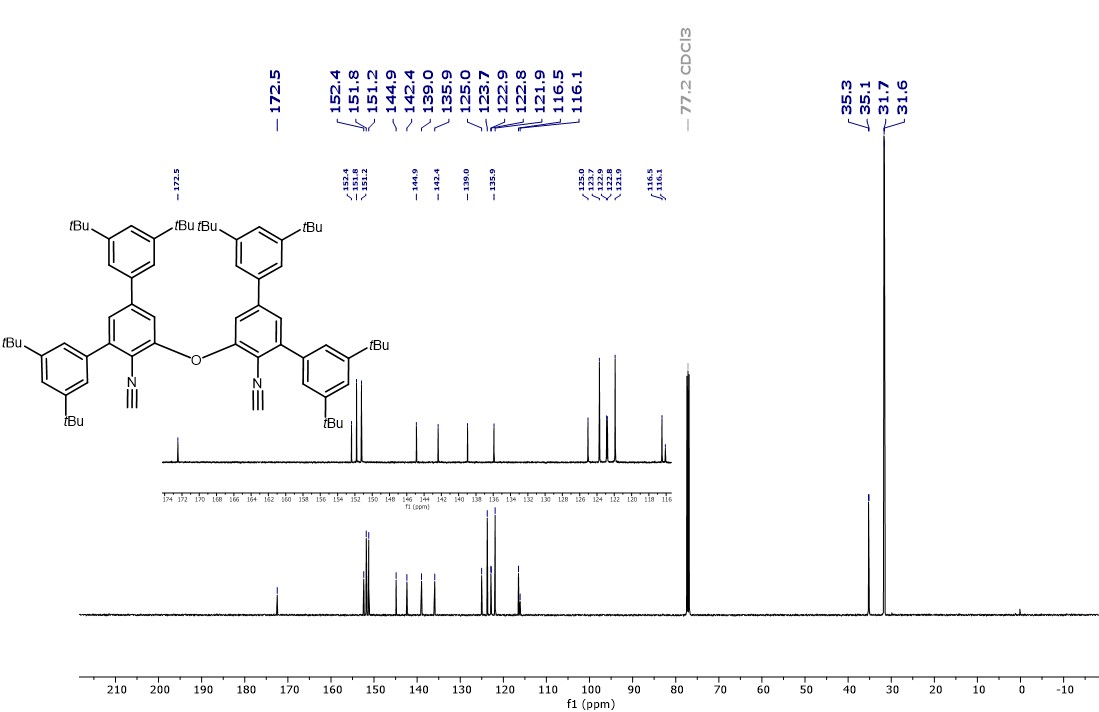


Figure S25: ^13^C{^1^H} NMR spectrum (126 MHz, 298 K) of **L^tBu2^** in CDCl_3_.


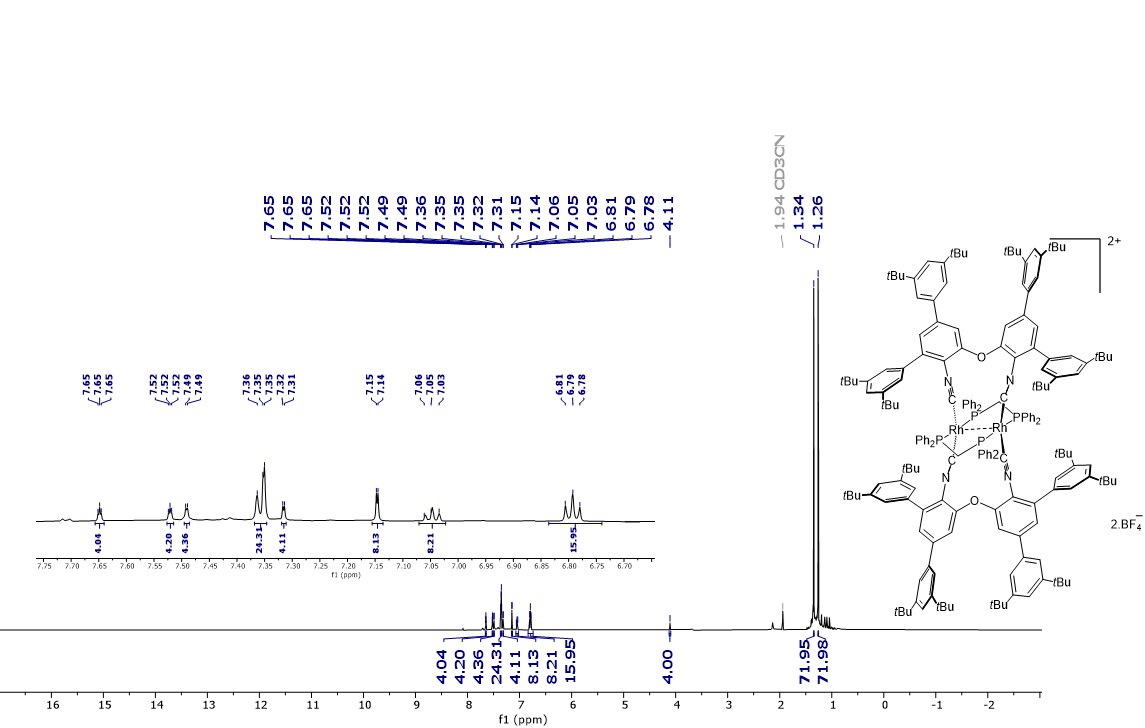


Figure S26: ^1^H{^31^P} NMR spectrum (600 MHz, 298 K) of **[Het-Rh_2_]*^t^*^Bu2^** in CD_3_CN.


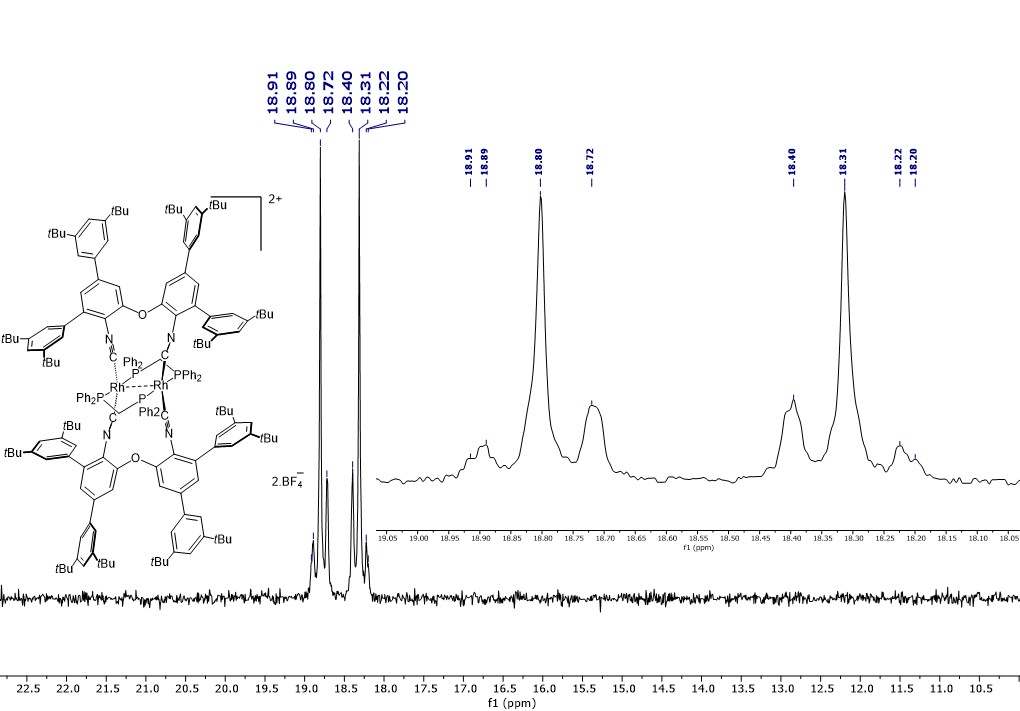


Figure S27: ^31^P{^1^H} NMR spectrum (243 MHz, 298 K) of **[Het-Rh_2_]^tBu2^** in CD_3_CN.


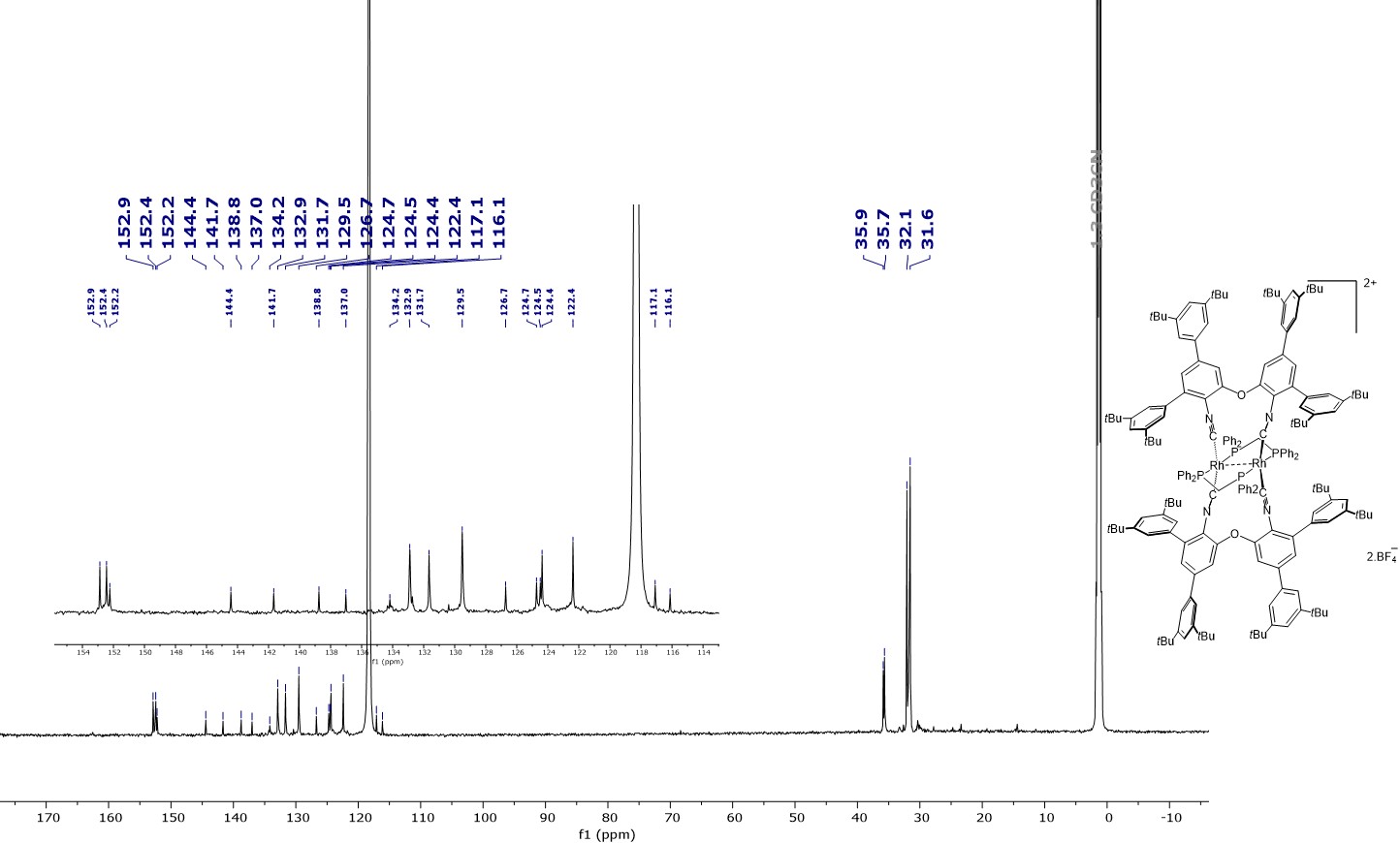


CD_3_CN

CD_3_CN

Figure S28: ^13^C{^1^H} NMR spectrum (151 MHz, 298 K) of **[Het-Rh_2_]^tBu2^** in CD_3_CN.


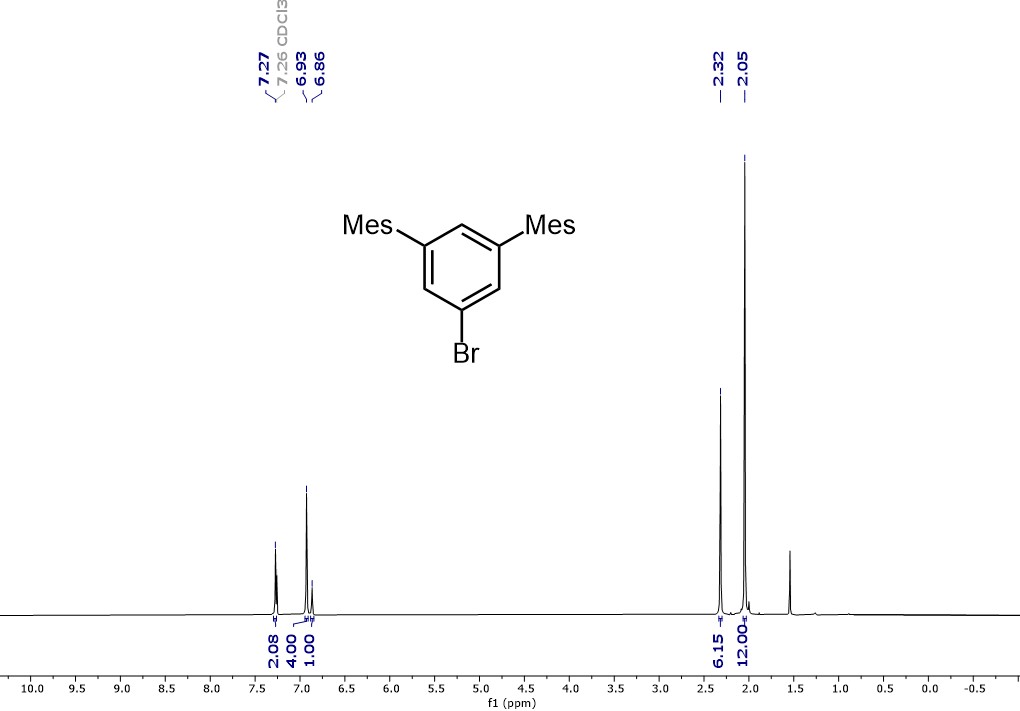


Figure S29: ^1^H NMR spectrum (400 MHz, 298 K) of **S8** in CDCl_3_.


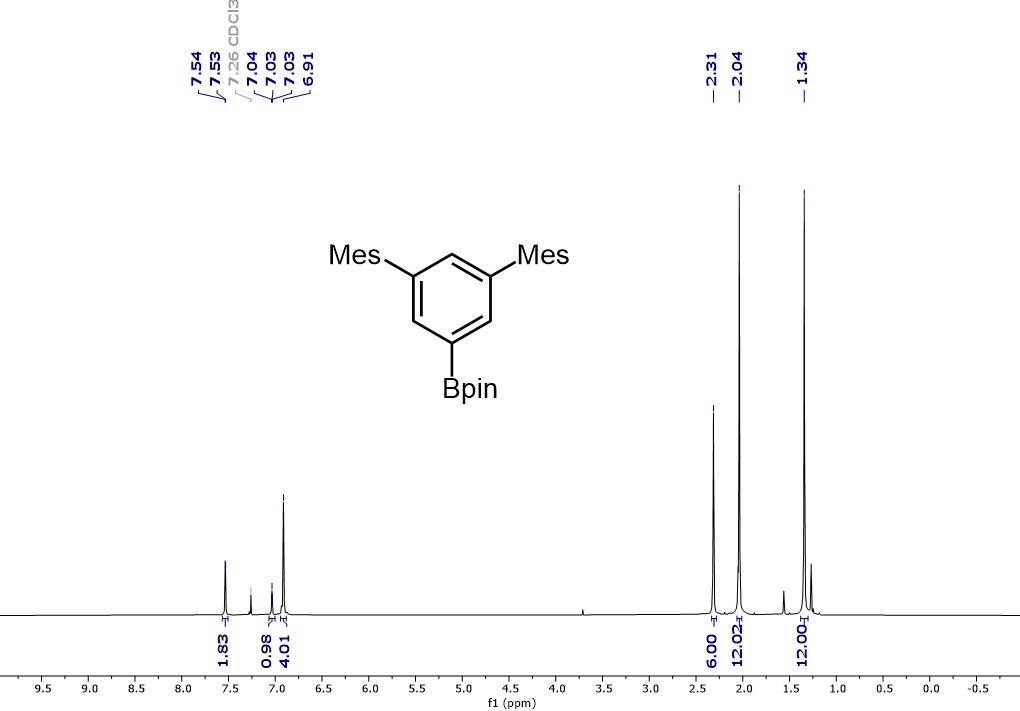


Figure S30: ^1^H NMR spectrum (400 MHz, 298 K) of **S9** in CDCl_3_.


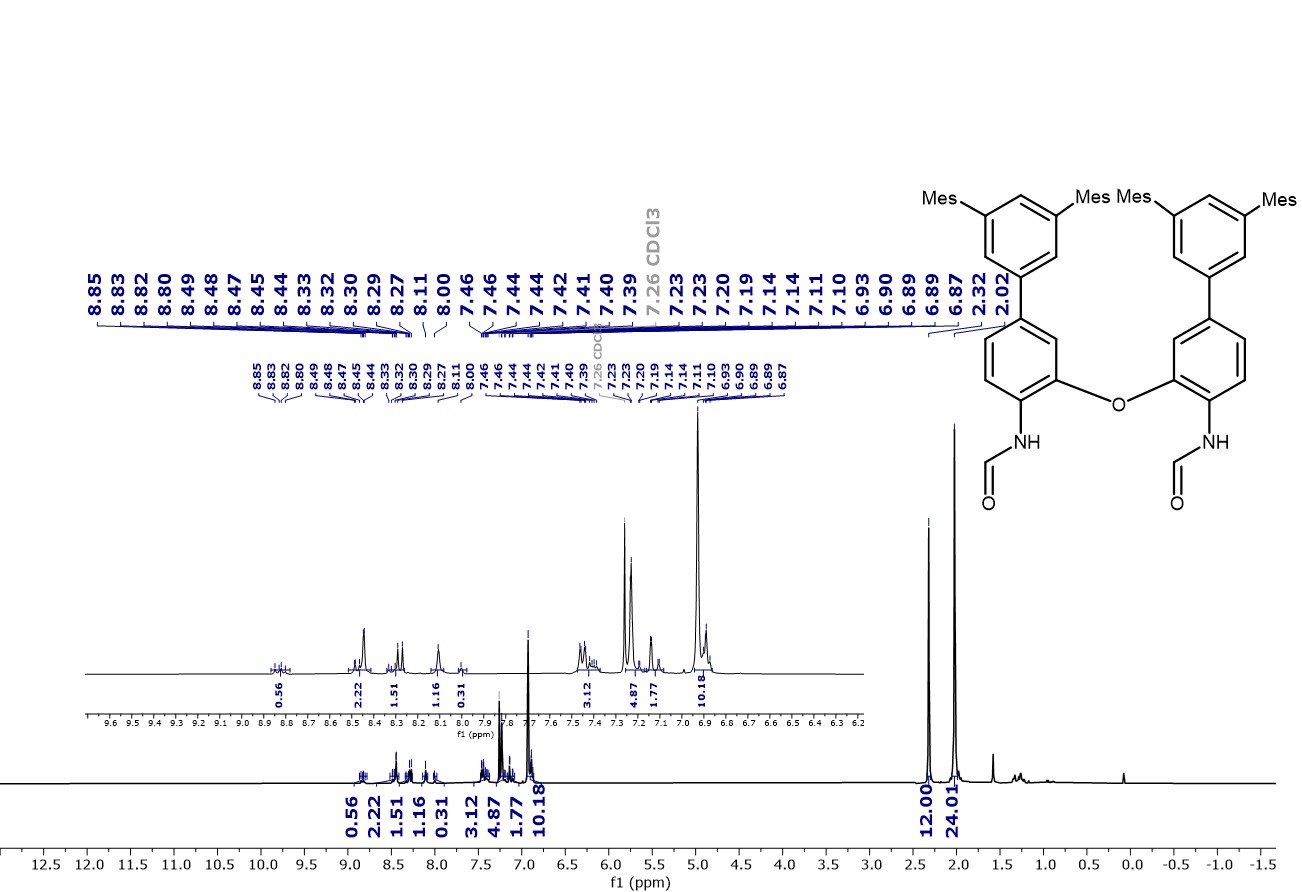


Figure S31: ^1^H NMR spectrum (400 MHz, 298 K) of **S10** in CDCl_3_.


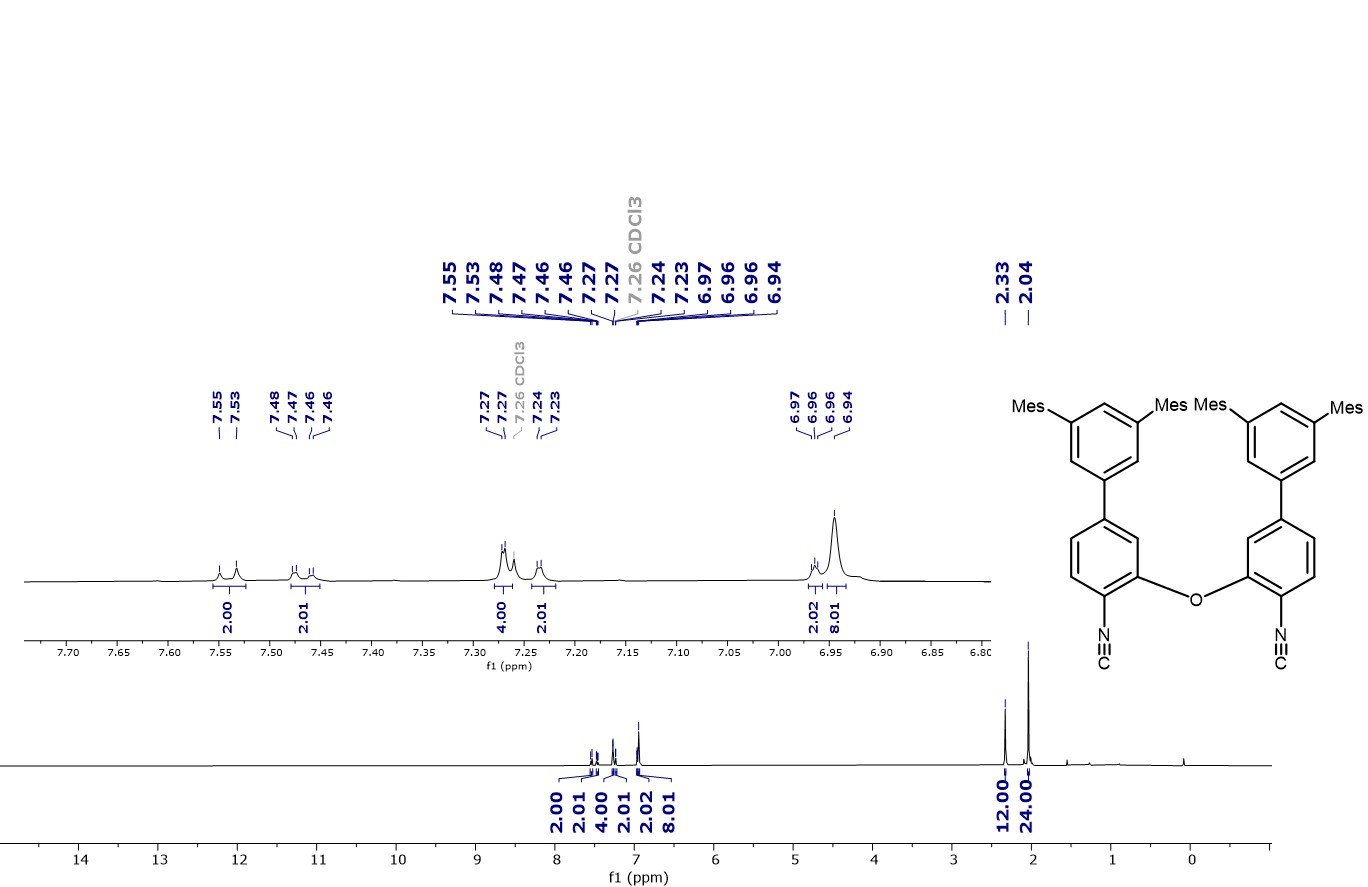


Figure S32: ^1^H NMR spectrum (500 MHz, 298 K) of **L^Mes^** in CDCl_3_.


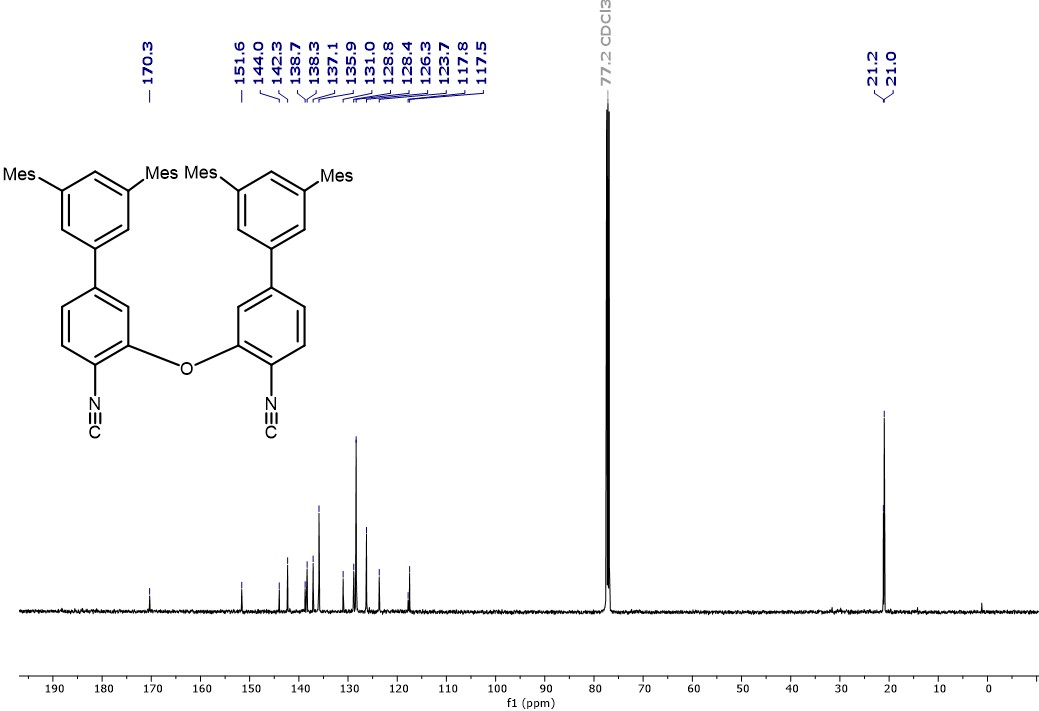


Figure S33: ^13^C{^1^H} NMR spectrum (126 MHz, 298 K) of **L^Mes^** in CDCl_3_.


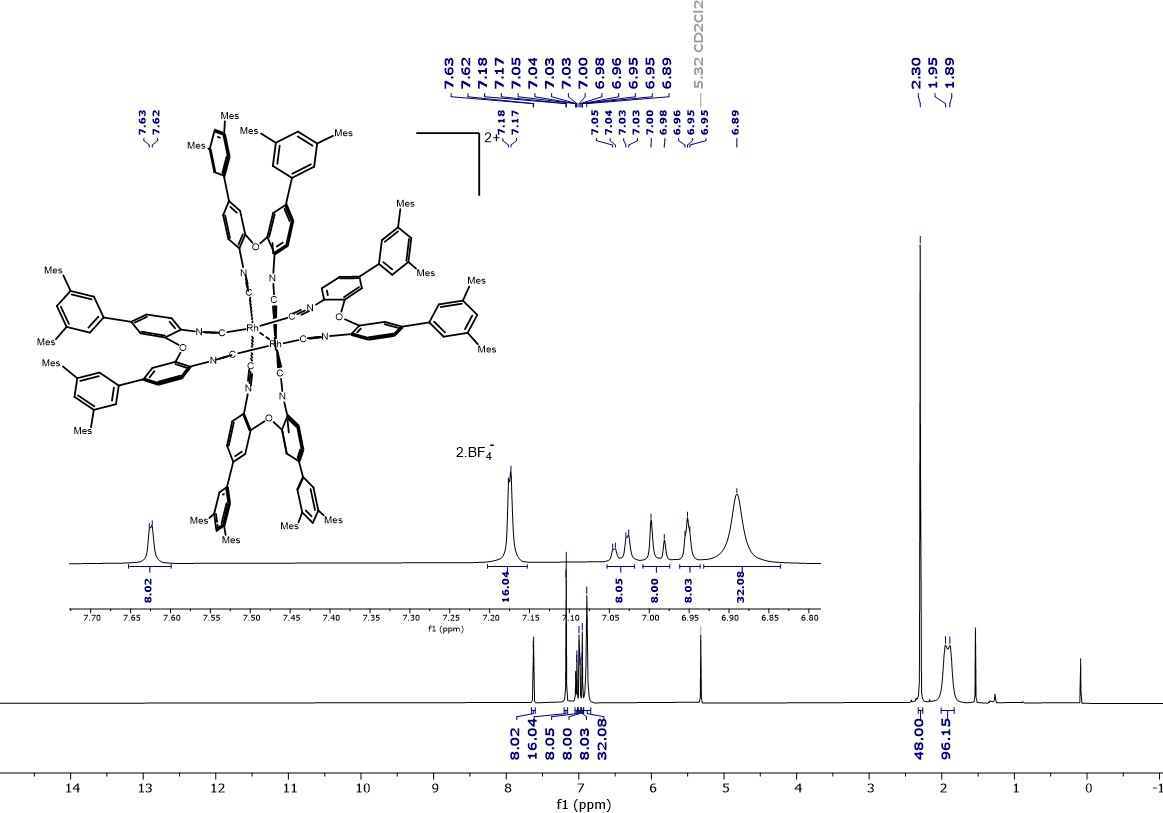


Figure S34: ^1^H NMR spectrum (500 MHz, 298 K) of **[Hom-Rh_2_]^Mes^** in CD_2_Cl_2_.


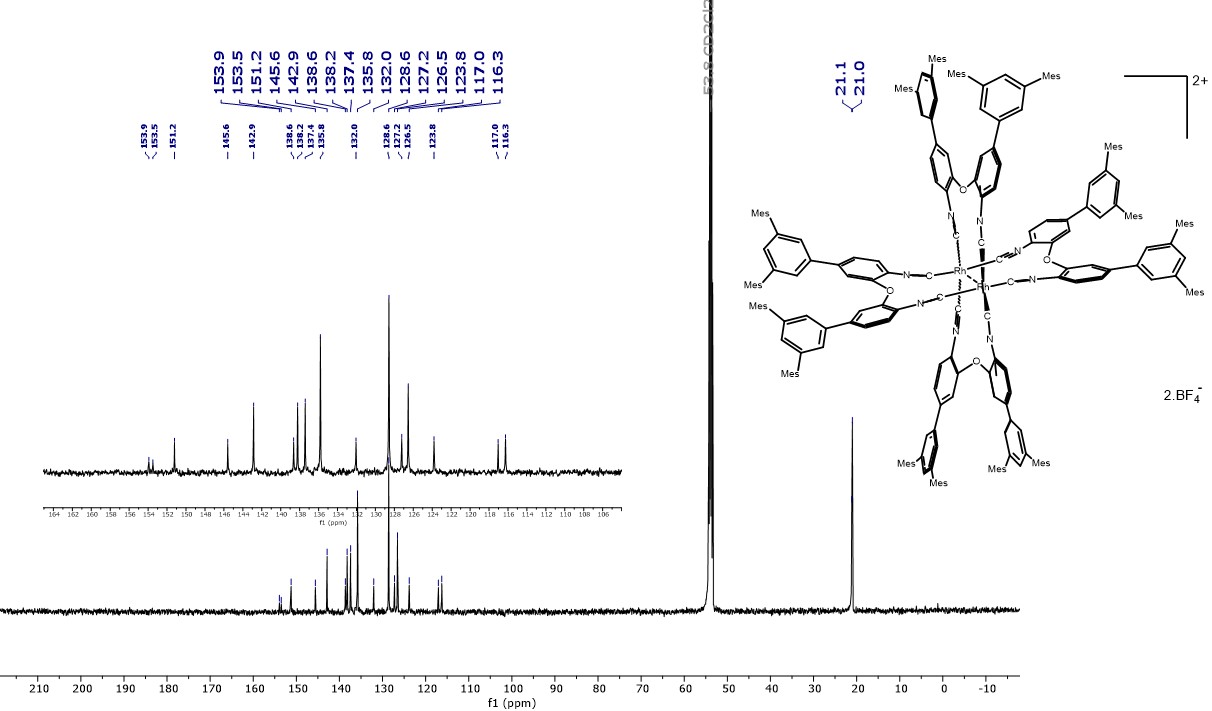


CD_2_Cl_2_

Figure S35: ^13^C{^1^H} NMR spectrum (126 MHz, 298 K) of **[Hom-Rh_2_]^Mes^** in CD_2_Cl_2_.

# **HRMS-ESI spectra**


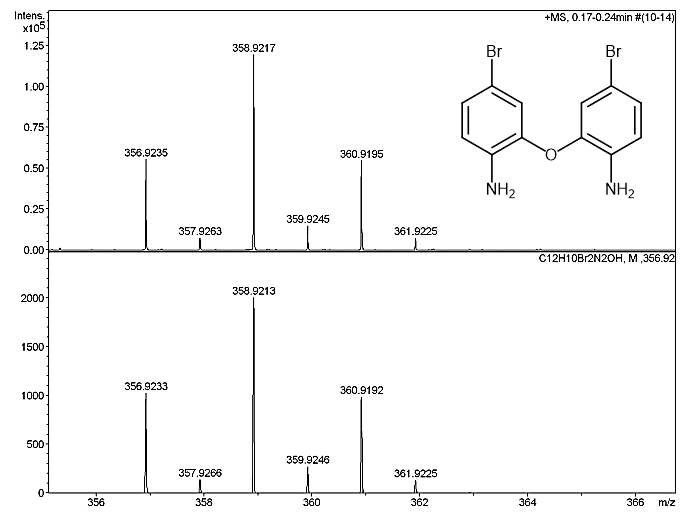


Figure S36: Top: HRMS-ESI (positive ions) mass spectrum of compound **S1**. Bottom: Simulated mass spectrum of [**S1**+H]^+^.


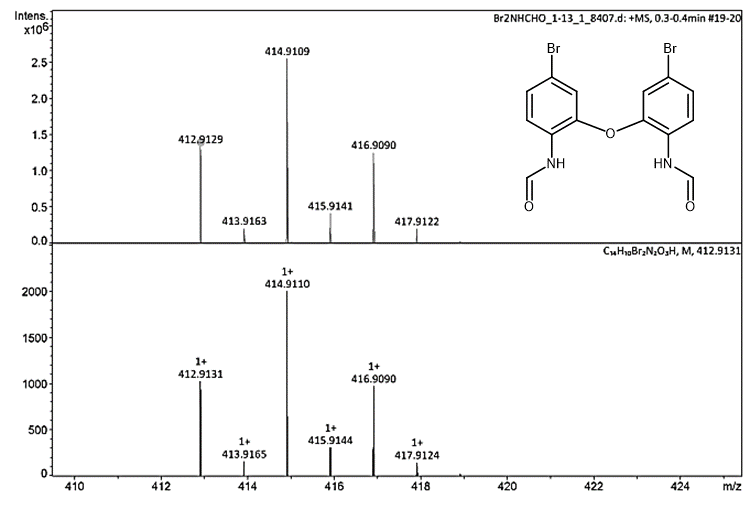


Figure S37: Top: HRMS-ESI (positive ions) mass spectrum of compound **S2**. Bottom: Simulated mass spectrum of [**S2**+H]^+^.


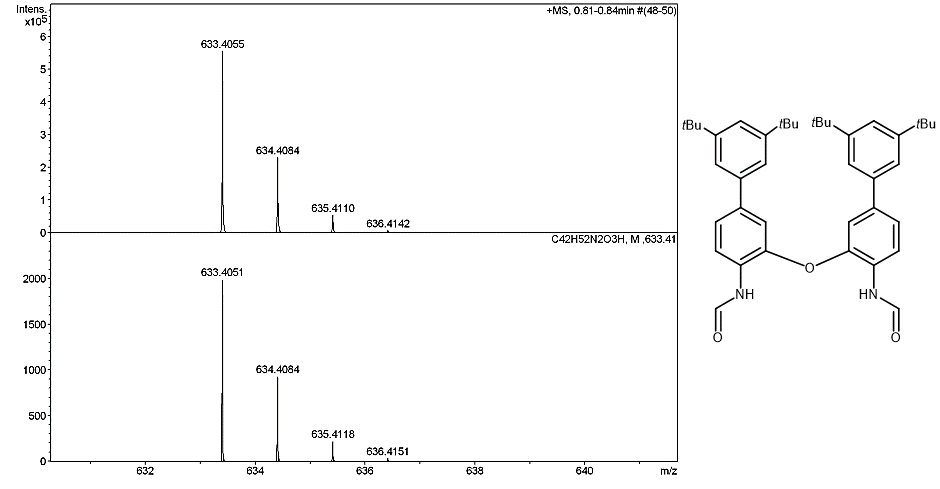


Figure S38: Top: HRMS-ESI (positive ions) mass spectrum of compound **S3**. Bottom: Simulated mass spectrum of [**S3**+H]^+^.


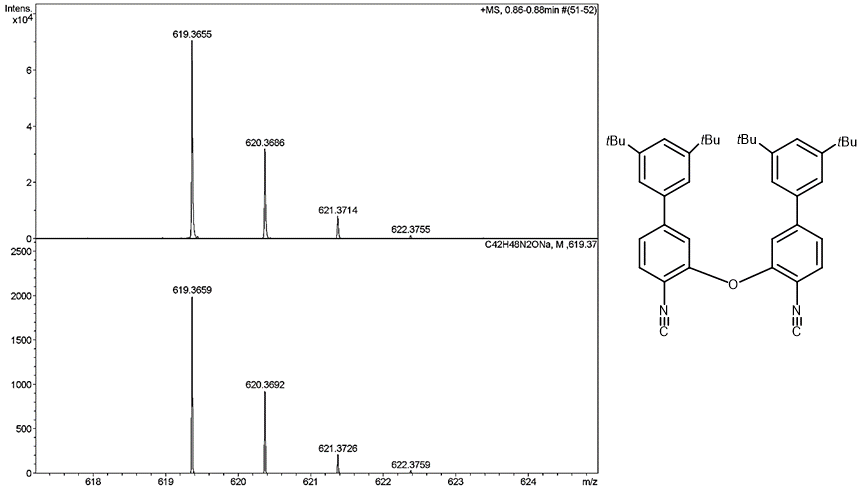


Figure S39: Top: HRMS-ESI (positive ions) mass spectrum of compound **L^tBu^**. Bottom: Simulated mass spectrum of [**L^tBu^** +Na]^+^.


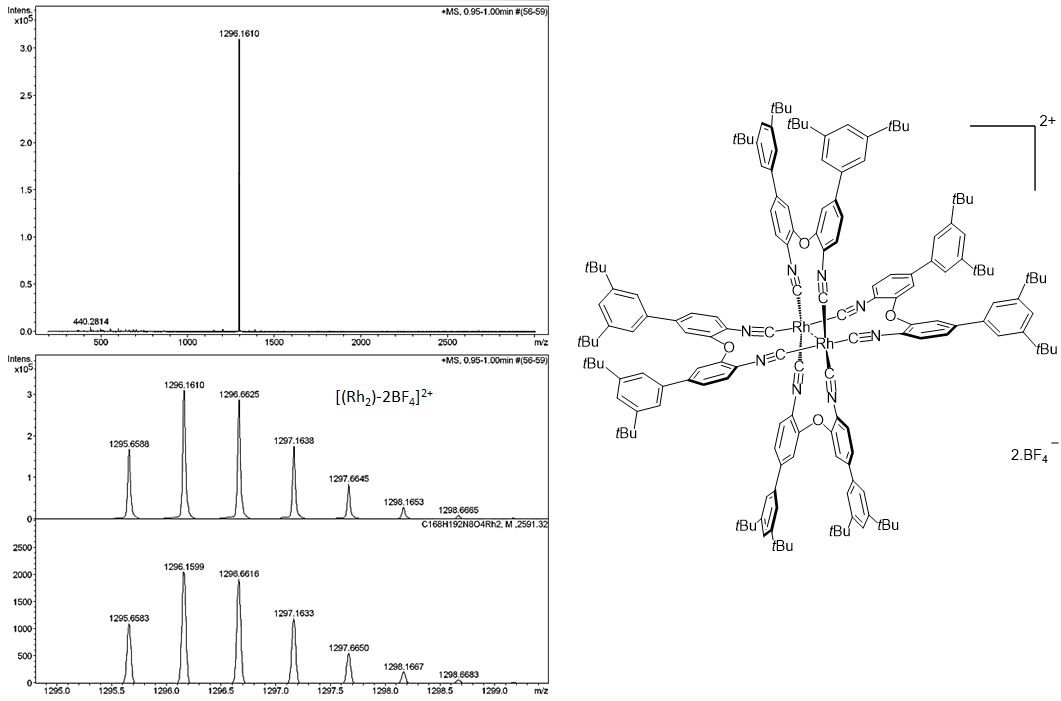


Figure S40: Top and middle: HRMS-ESI (positive ions) mass spectrum of compound **{[Hom-Rh_2_]^tBu^-2BF_4_}^2+^**. Bottom: Simulated mass spectrum of **{[Hom-Rh_2_]^tBu^-2BF_4_}^2+^**.


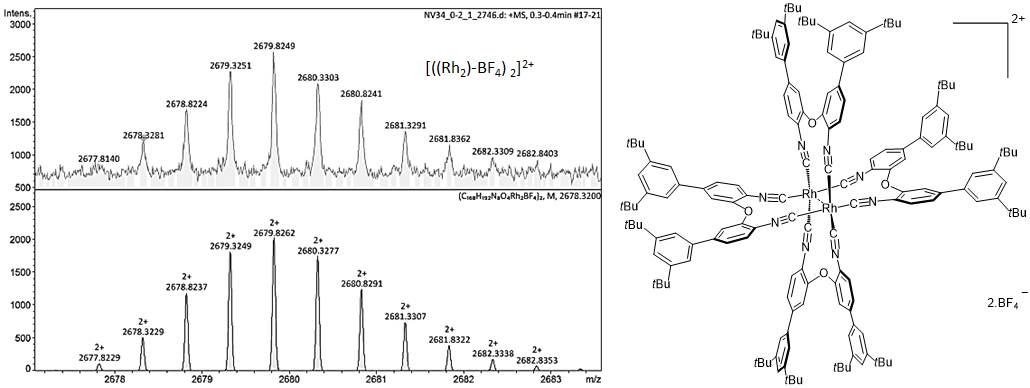


Figure S41: Top: HRMS-ESI (positive ions) mass spectrum of compound **{([Hom-Rh_2_]^tBu^-BF_4_)_2_}^2+^**. Bottom: Simulated mass spectrum of **{([Hom-Rh_2_]^tBu^-BF_4_)_2_}^2+^.**


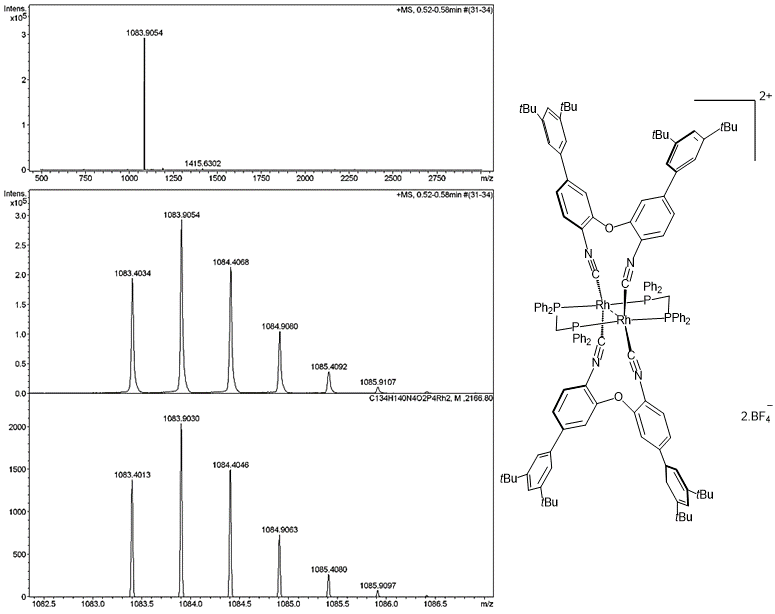


Figure S42: Top and middle: HRMS-ESI (positive ions) mass spectrum of compound **{[Het-Rh_2_]^tBu^-2BF_4_}^2+^.** Bottom: Simulated mass spectrum of **{[Het-Rh_2_]^tBu^-2BF_4_}^2+^.**


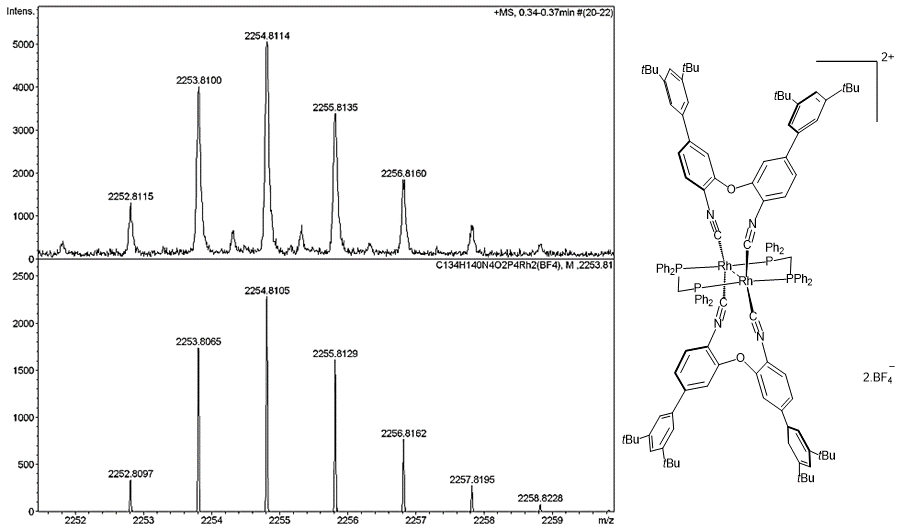


Figure S43: Top: HRMS-ESI (positive ions) mass spectrum of compound **{[Het-Rh_2_]^tBu^-BF_4_}^+^**. Bottom: Simulated mass spectrum of **{[Het-Rh_2_]^tBu^-BF_4_}^+^.**


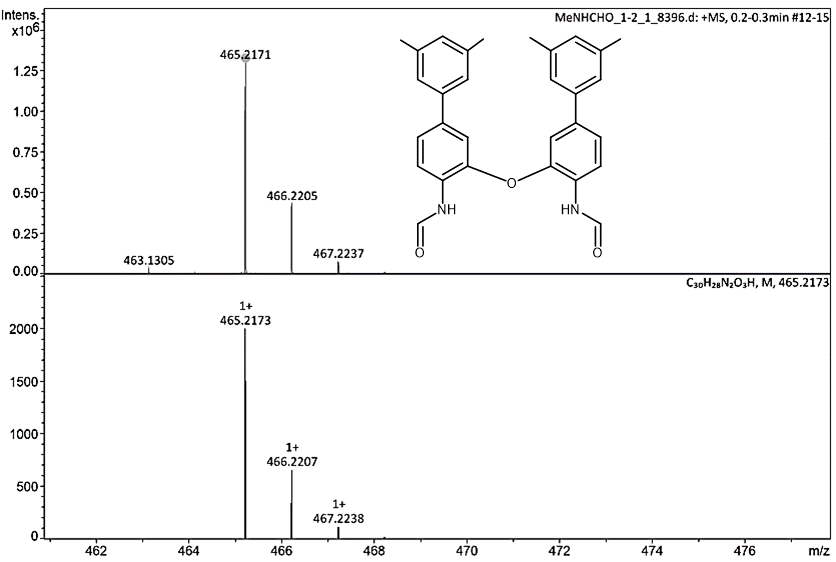


Figure S44: Top: HRMS-ESI (positive ions) mass spectrum of compound **S4**. Bottom: Simulated mass spectrum of [**S4**+H]^+^.


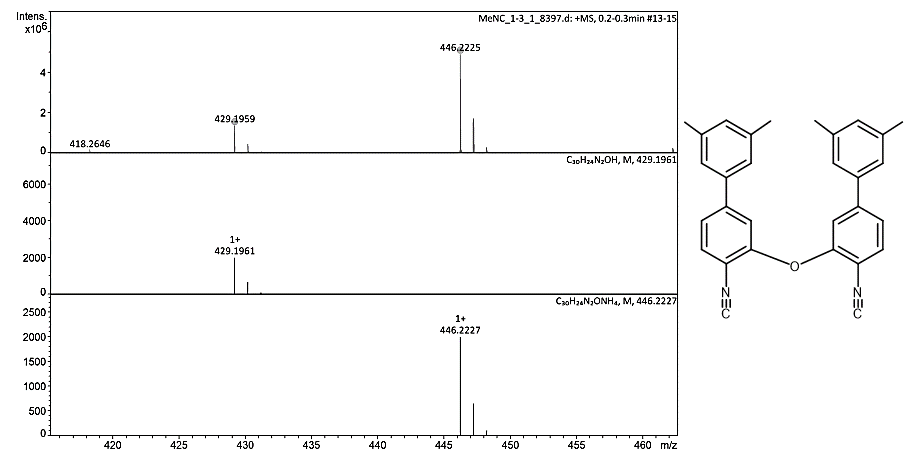


Figure S45: Top: HRMS-ESI (positive ions) mass spectrum of compound **L^Me^**. Middle: Simulated mass spectrum of [**L^Me^**+H]^+^. Bottom: Simulated mass spectrum of [**L^Me^**+NH_4_]^+^.


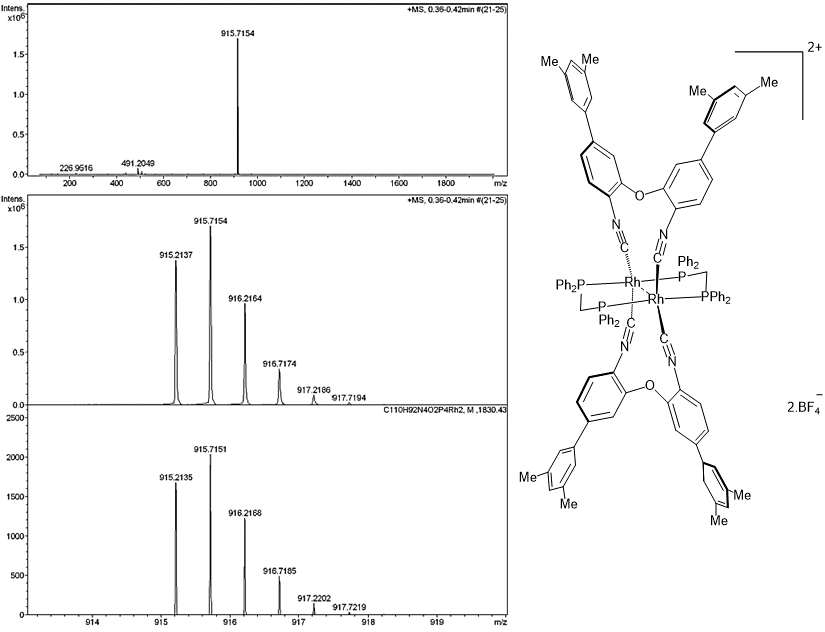


Figure S46: Top and middle: HRMS-ESI (positive ions) mass spectrum of compound **{[Het-Rh_2_]^Me^-2BF_4_}^2^**^+^. Bottom: Simulated mass spectrum of **{[Het-Rh_2_]^Me^-2BF_4_}^2+^.**


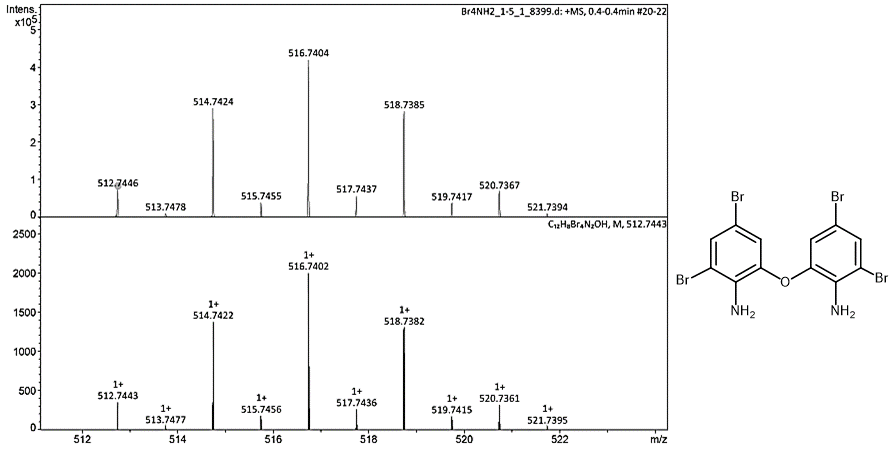


Figure S47: Top: HRMS-ESI (positive ions) mass spectrum of compound **S5**. Bottom: Simulated mass spectrum of [**S5**+H]^+^.


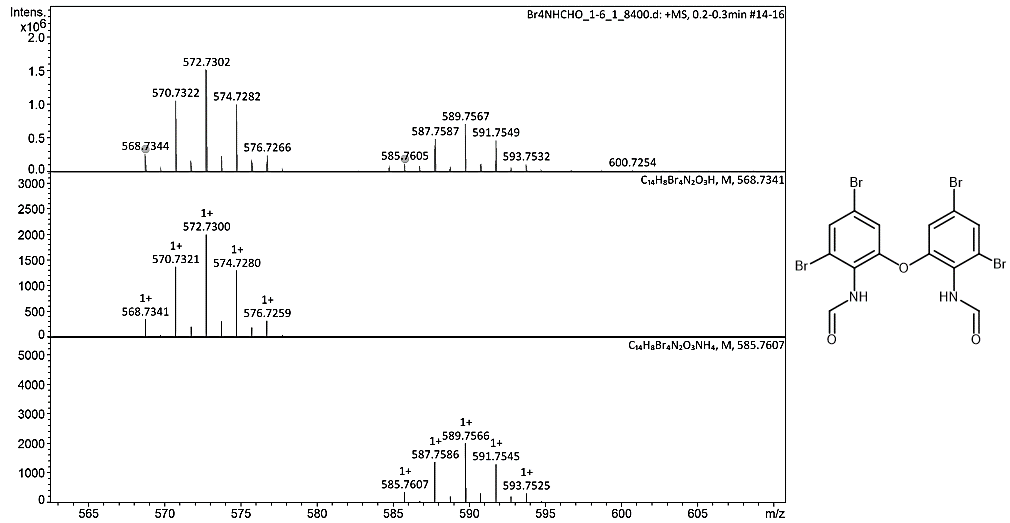


Figure S48: Top: HRMS-ESI (positive ions) mass spectrum of compound **S6**. Middle: Simulated mass spectrum of [**S6**+H]^+^. Bottom: Simulated mass spectrum of [**S6**+NH_4_]^+^.


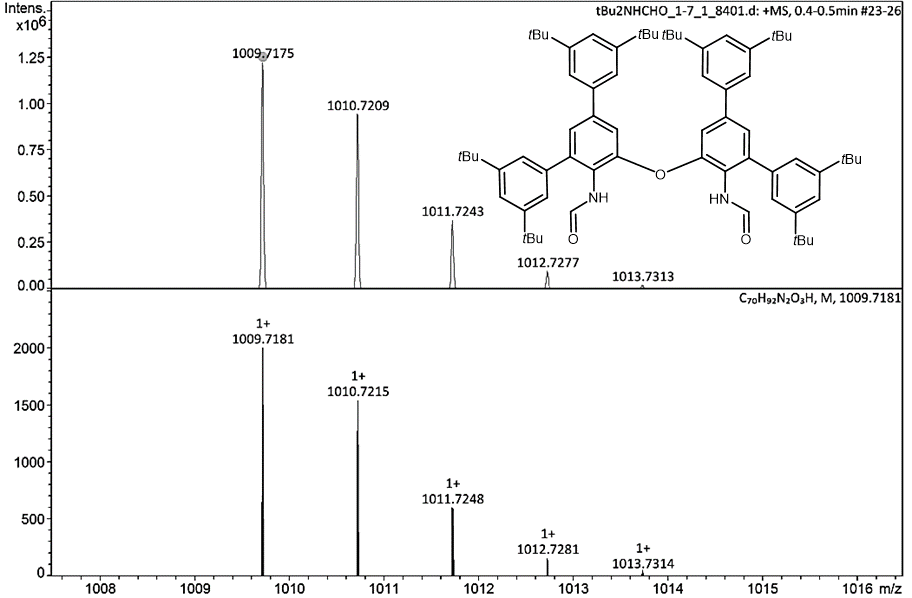


Figure S49: Top: HRMS-ESI (positive ions) mass spectrum of compound **S7**. Bottom: Simulated mass spectrum of [**S7**+H]^+^.


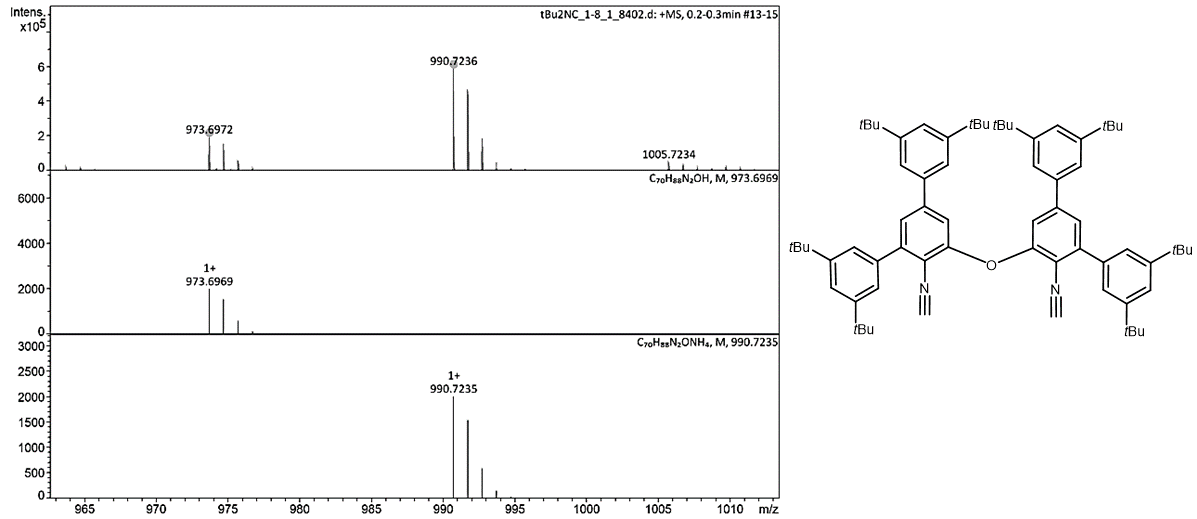


Figure S50: Top: HRMS-ESI (positive ions) mass spectrum of compound **L^tBu2^**. Middle: Simulated mass spectrum of [**L^tBu2^**+H]^+^. Bottom: Simulated mass spectrum of [**L^tBu2^**+NH_4_]^+^.


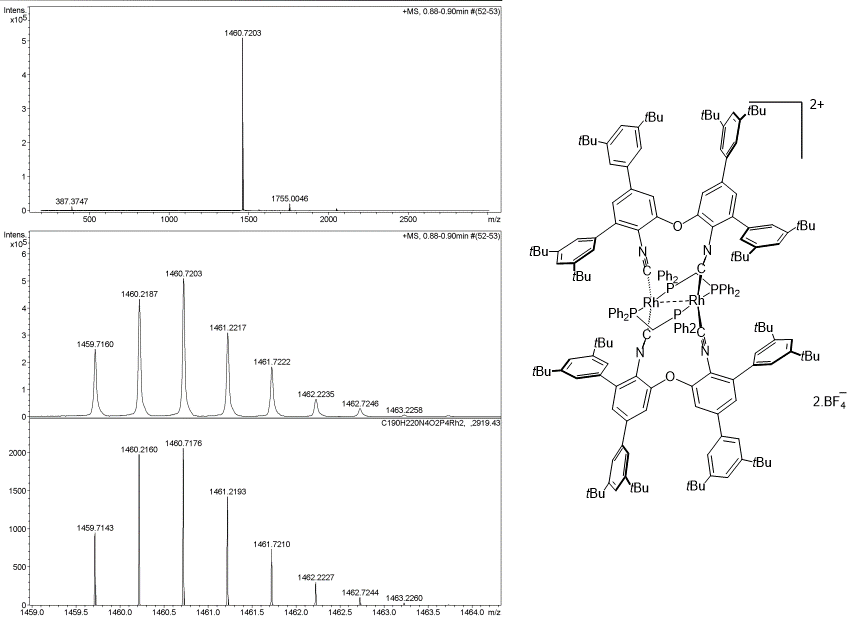


Figure S51: Top and middle: HRMS-ESI (positive ions) mass spectrum of compound **{[Het-Rh_2_]^tBu2^-2BF_4_}^2+^.** Bottom: Simulated mass spectrum of **{[Het-Rh_2_]^tBu2^-2BF_4_}^2+^.**


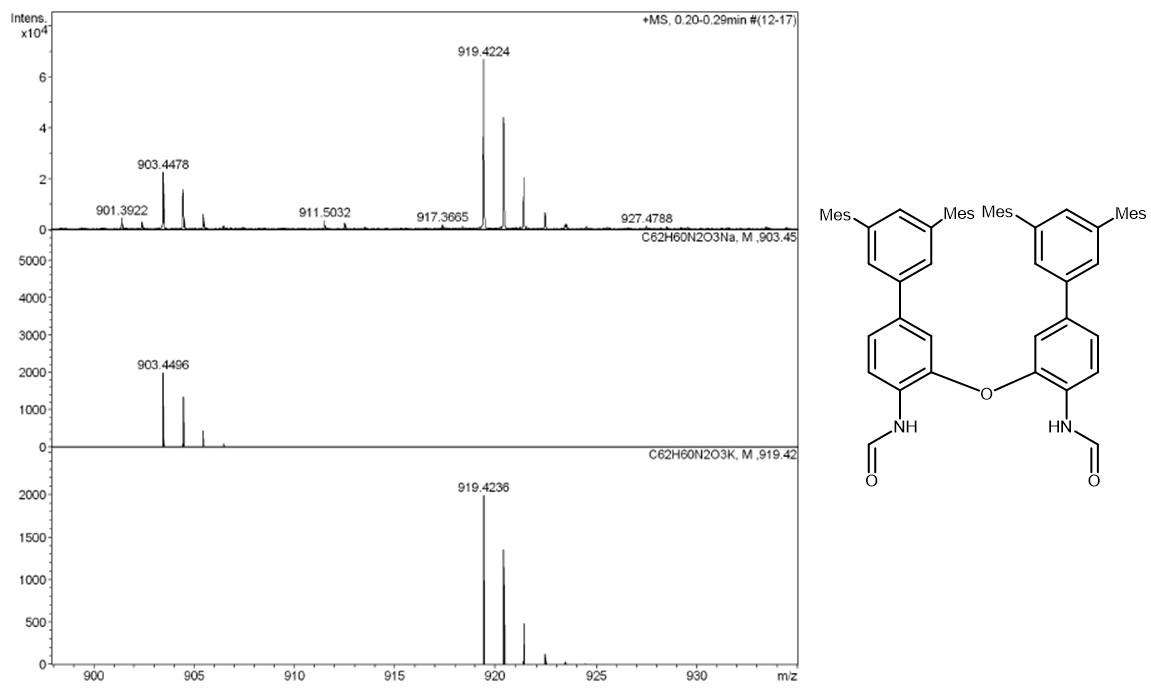


Figure S52: Top: HRMS-ESI (positive ions) mass spectrum of compound **S10**. Middle: Simulated mass spectrum of [**S10**+Na]^+^. Bottom: Simulated mass spectrum of [**S10**+K]^+^.


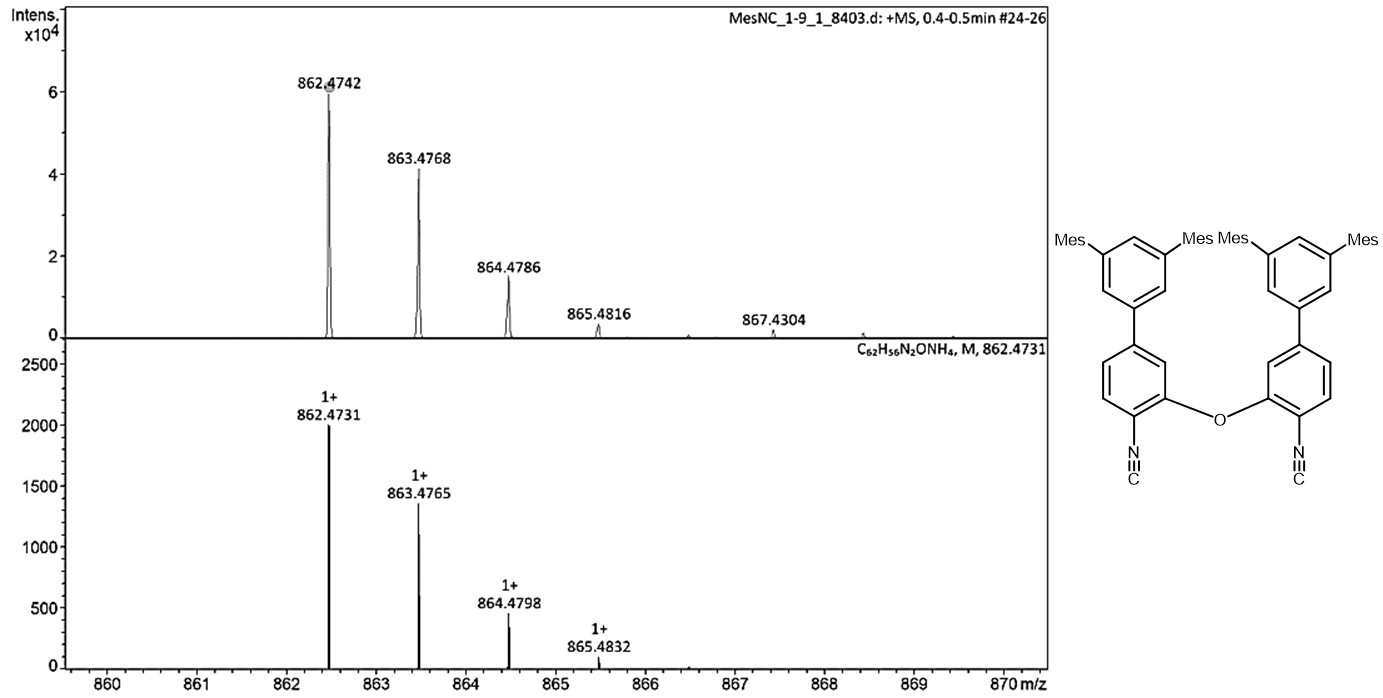


Figure S53: HRMS-ESI (positive ions) mass spectrum of compound **L^Mes^**. Bottom: Simulated mass spectrum of [**L^Mes^**+NH_4_]^+^.


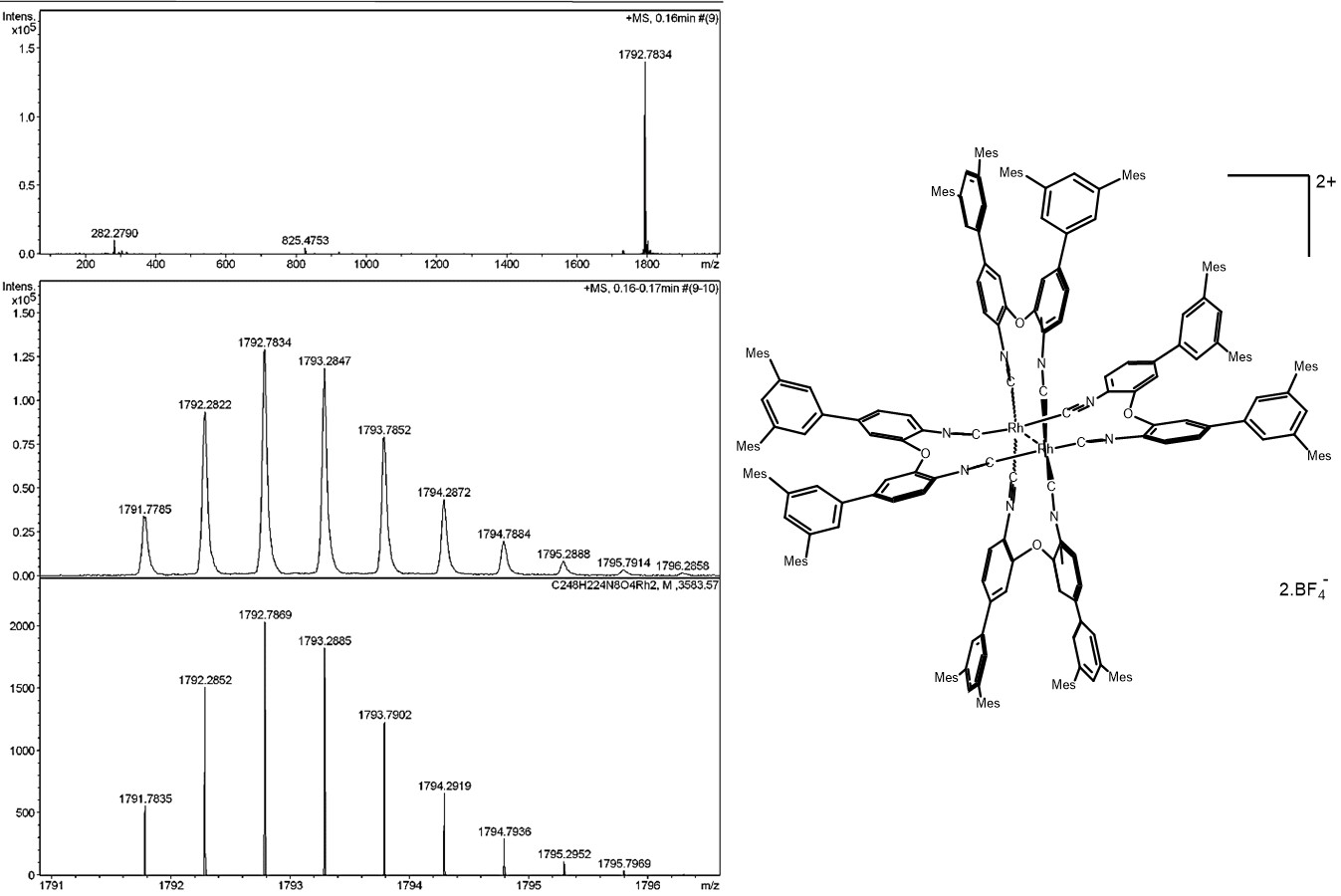


Figure S54: Top and middle: HRMS-ESI (positive ions) mass spectrum of compound **{[Hom-Rh_2_]^Mes^-2BF_4_}^2+^.** Bottom: Simulated mass spectrum of **{[Hom-Rh_2_]^Mes^-2BF_4_}^2+^.**

# **Infrared spectra**


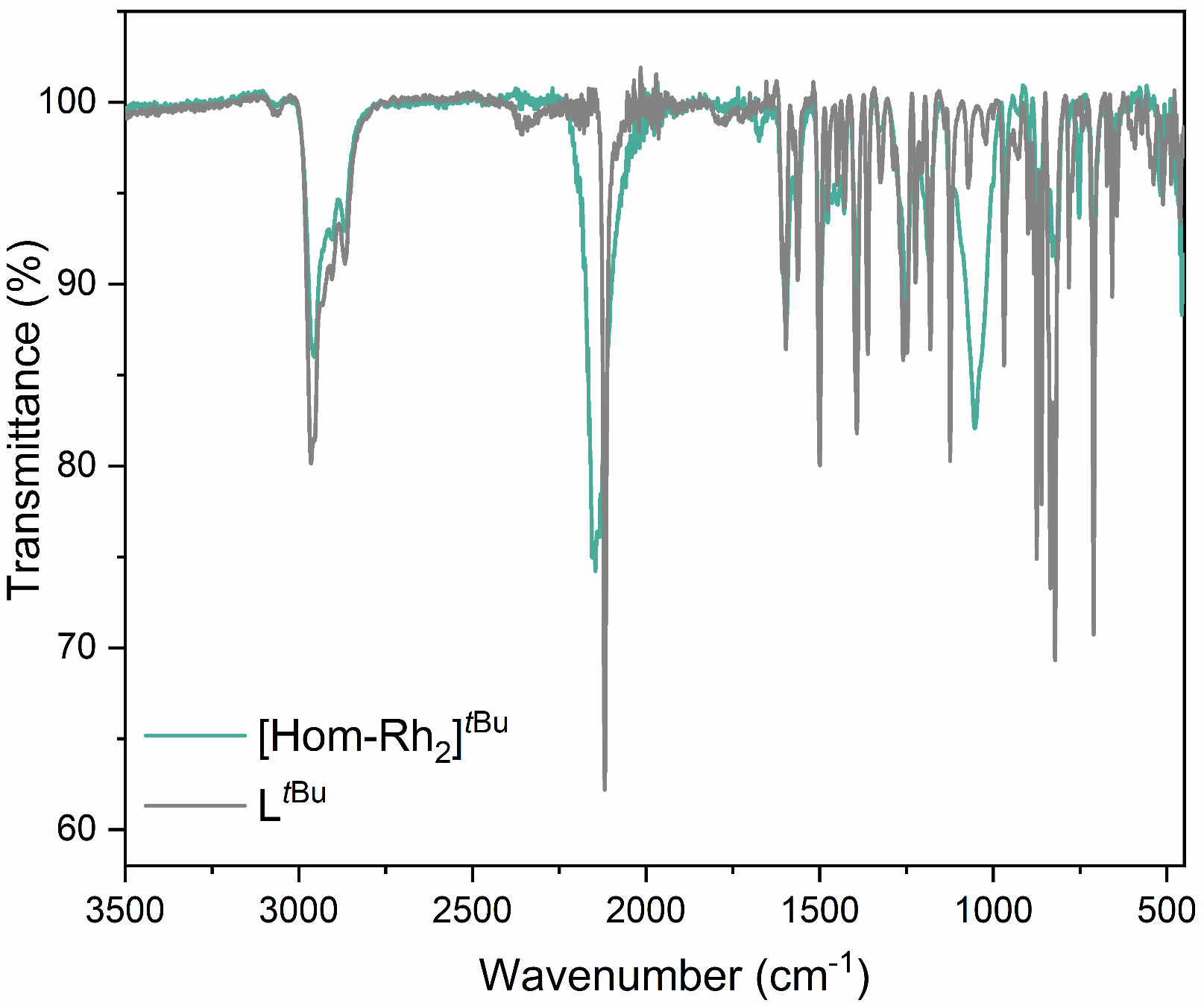


Figure S55: Solid state IR spectra of **L^tBu^** and **[Hom-Rh_2_]^tBu^**.


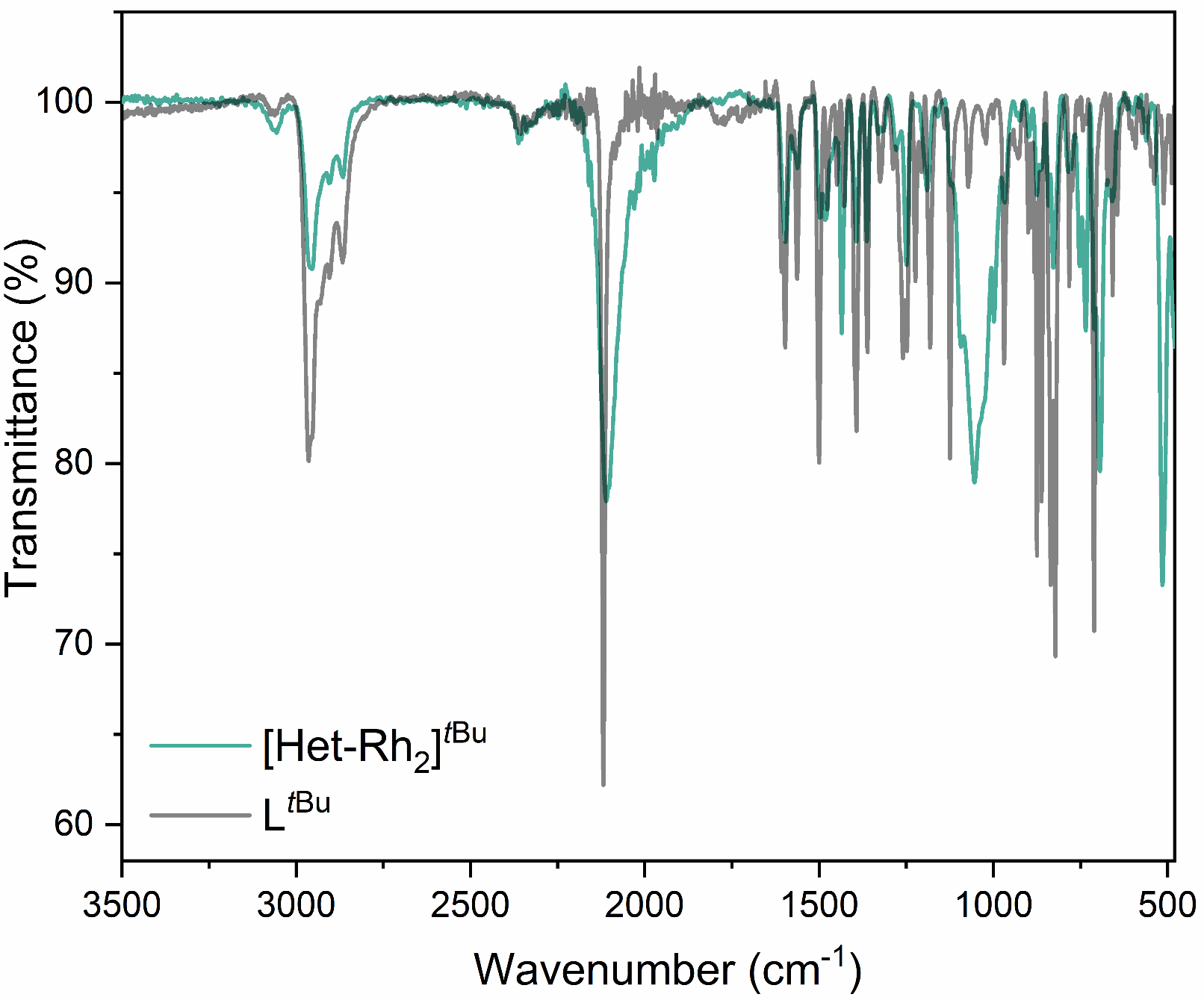


Figure S56: Solid state IR spectra of **L^tBu^** and **[Het-Rh_2_]^tBu^**.


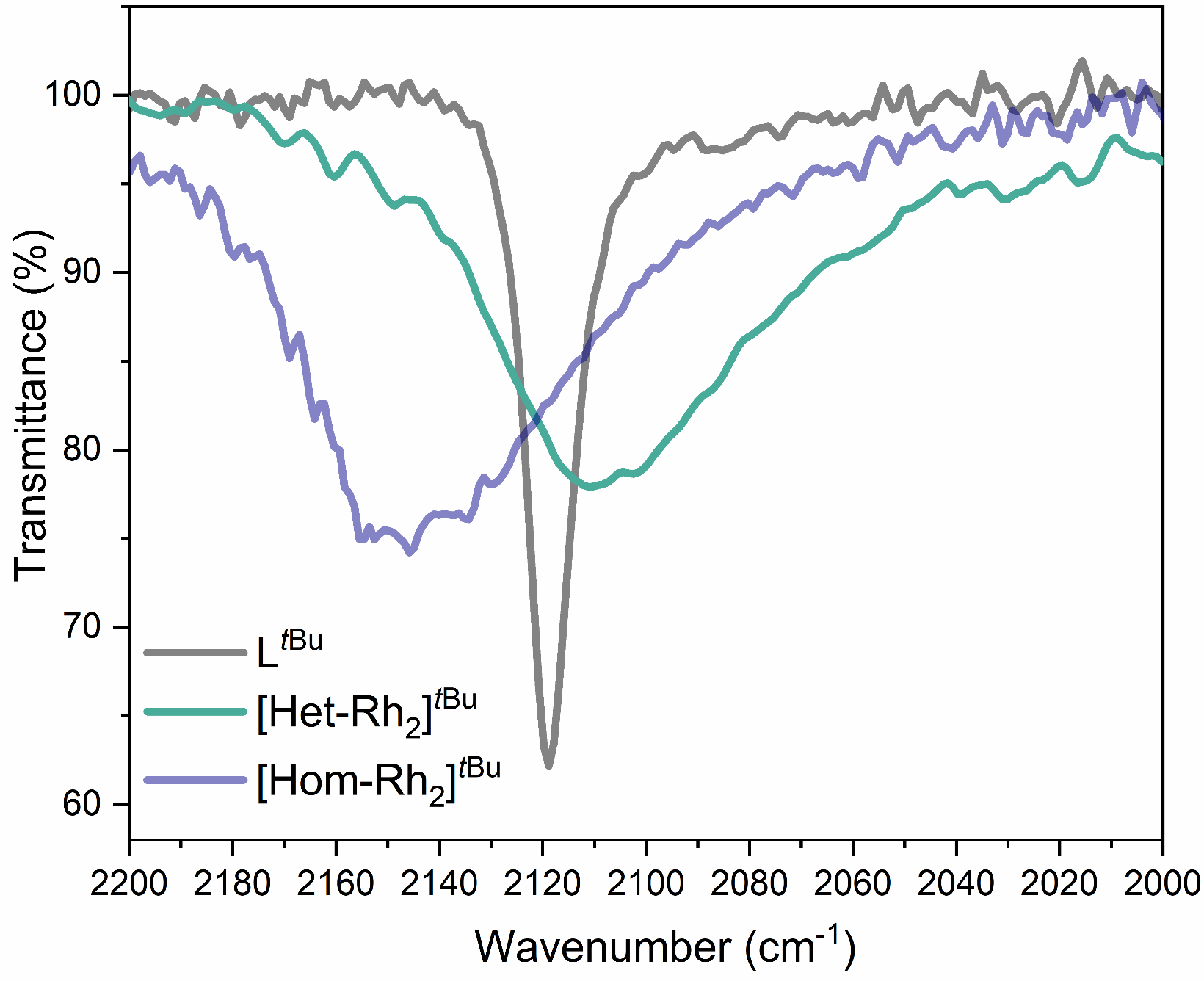


Figure S57: Comparison of the C≡N stretching frequencies of **L^tBu^**, **[Hom-Rh_2_]^tBu^** and **[Het-Rh_2_]^tBu^** in the solid state IR.


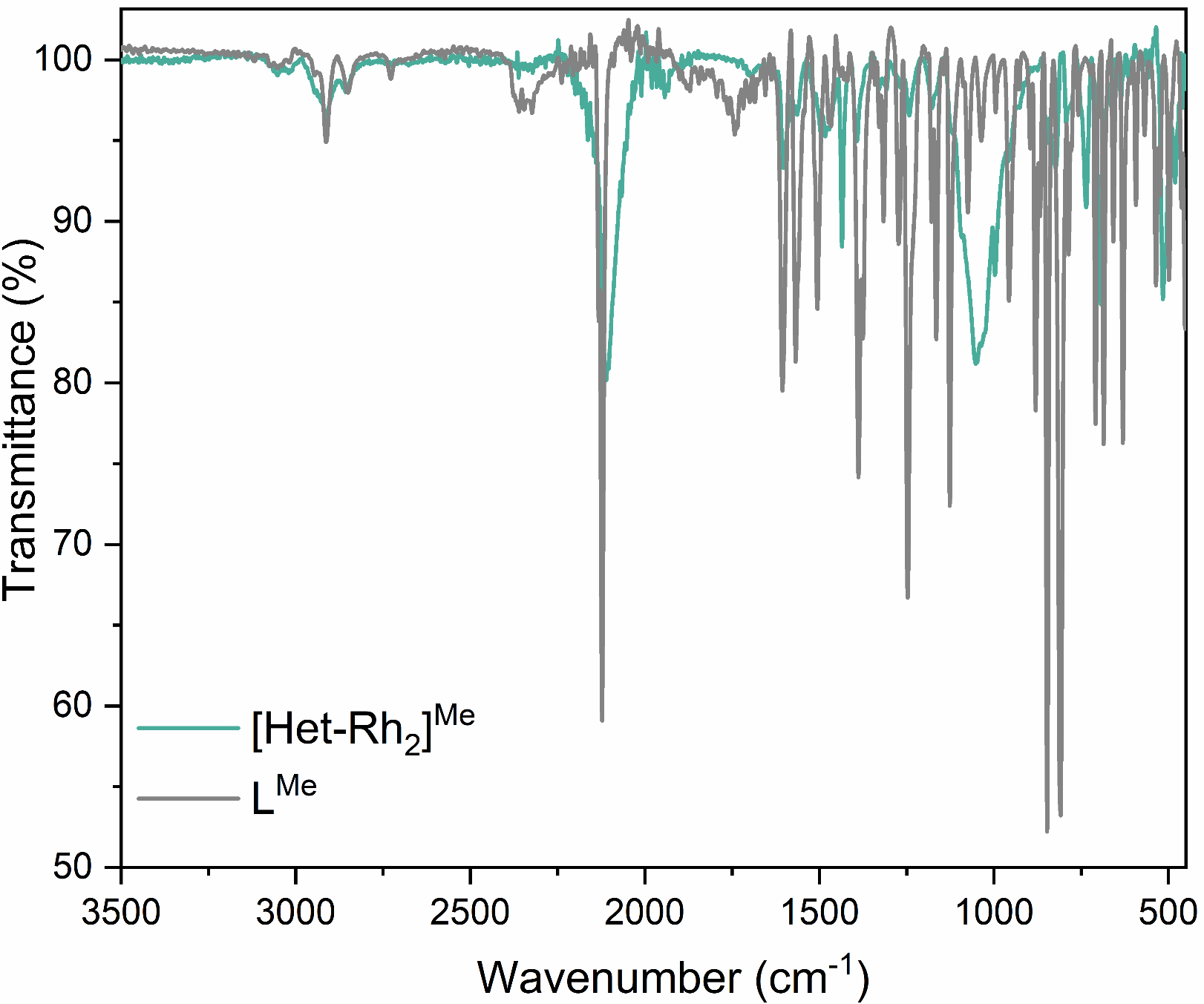


Figure S58: Solid state IR spectra of **L^Me^** and **[Het-Rh_2_]^Me^.**


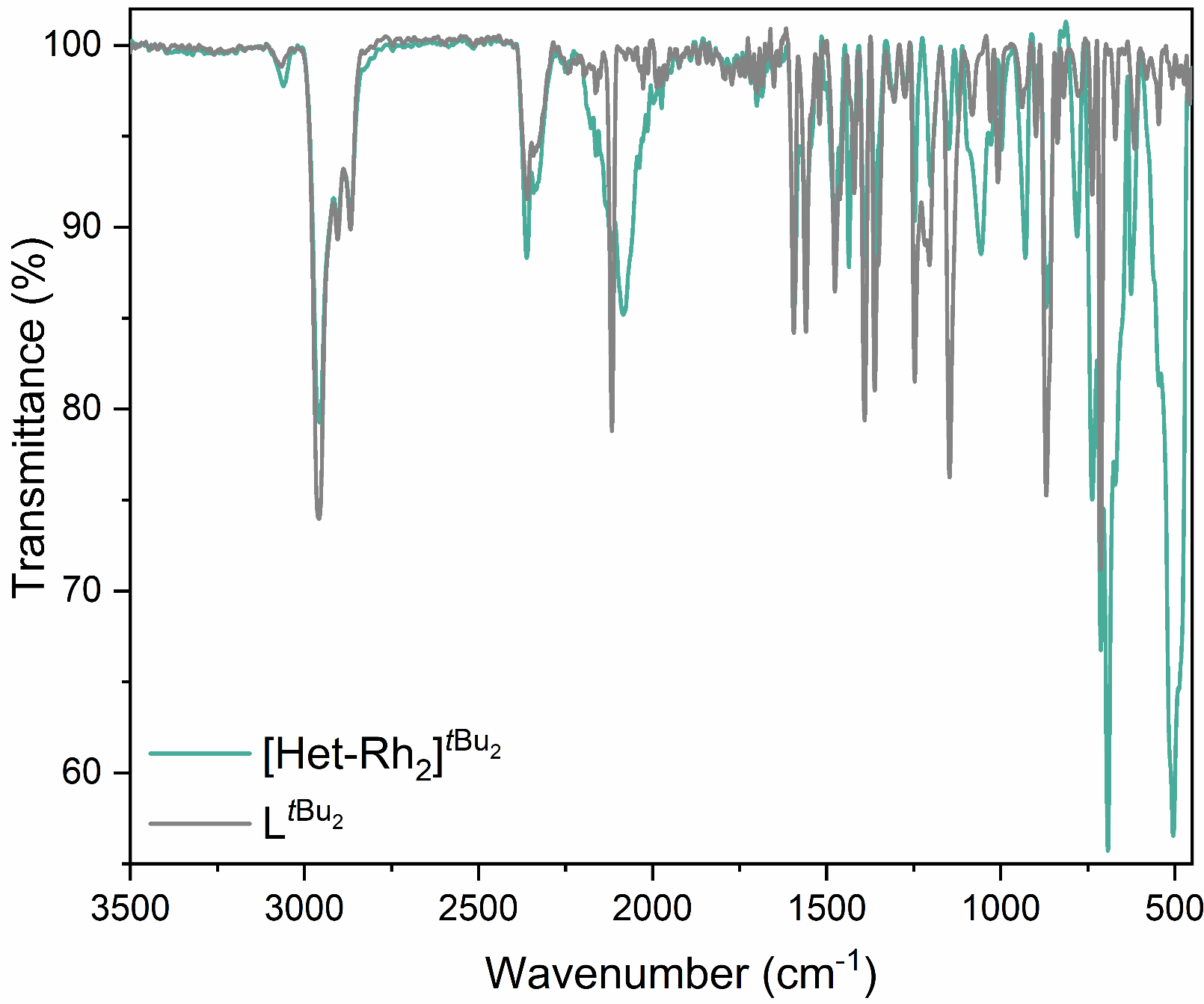


Figure S59: Solid state IR spectra of **L^tBu2^** and **[Het-Rh_2_]^tBu2^**.


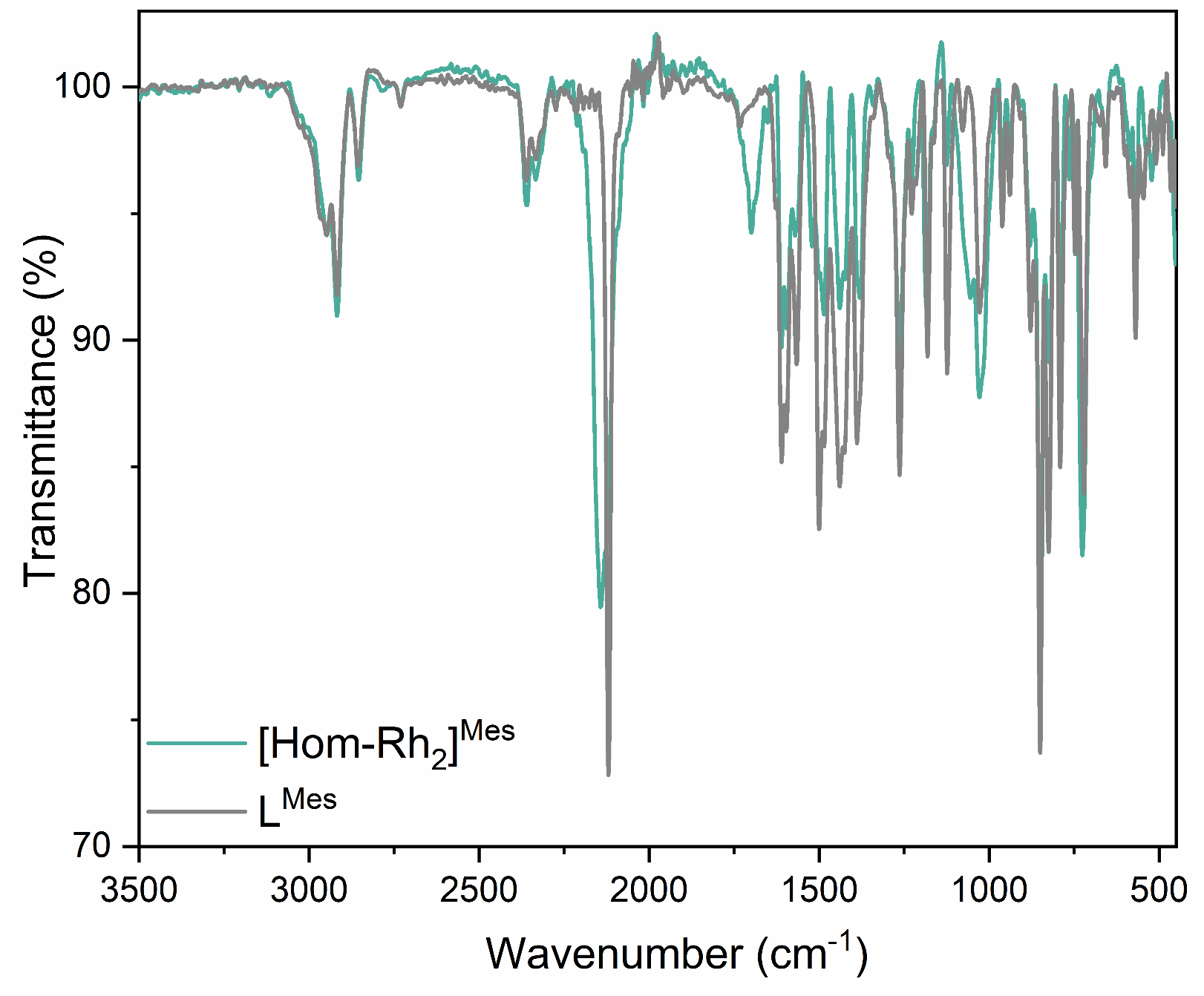


Figure S60: Solid state IR spectra of **L^Mes^** and **[Hom-Rh_2_]^Mes^.**

# **X-ray crystallography**

A suitable crystal was selected for each compound, and the crystal was mounted on a MITIGEN holder in perfluoroether oil on a STOE STADIVARI diffractometer. The crystal was kept at 150 K during data collection. Using Olex2,^[3]^ the structure was solved with the ShelXT^[4]^ structure solution program using Intrinsic Phasing and refined with the ShelXT^[4]^ refinement package using Least Squares minimization. The remaining non-hydrogen atoms were located from successive difference Fourier map calculations. The refinements were carried out by using full-matrix least-squares techniques on *F*^2^ by using the program SHELXL.^[3-4]^ The H-atoms were introduced into the geometrically calculated positions (SHELXL procedures) unless otherwise stated and refined riding on the corresponding parent atoms. In each case, the locations of the largest peaks in the final difference Fourier map calculations, as well as the magnitude of the residual electron densities, were of no chemical significance. Summary of the crystal data, data collection and refinement for all compounds are given in Table S1-S3.

Crystallographic data for the structures reported in this paper have been deposited with the Cambridge Crystallographic Data Centre as a supplementary publication no. 2522554-2522558. Copies of the data can be obtained free of charge on application to CCDC, 12 Union Road, Cambridge CB21EZ, UK (fax: (+(44)1223-336-033; email: deposit@ccdc.cam.ac.uk).

Table S1: Crystallographic data for **L^tBu^** and **[Hom-Rh_2_]^Mes^.**

| **Compound** | **L*^t^*^Bu^** | **[Hom-Rh_2_]^Mes^** |
| --- | --- | --- |
| CCDC number | 2522554 | 2522557 |
| Empirical formula | C_42_H_48_N_2_O | C_270.5_H_277_B_2_Cl_5_F_8_N_8_O_4_Rh_2_ |
| Formula weight | 596.82 | 4260.67 |
| Temperature/K | 150 | 150 |
| Crystal system | triclinic | monoclinic |
| Space group | *P*$\bar{1}$ | *C*2/*c* |
| a/Å | 9.9348(6) | 39.8482(4) |
| b/Å | 13.1066(7) | 32.1182(3) |
| c/Å | 14.5772(9) | 22.5730(3) |
| α/° | 95.644(5) | 90 |
| β/° | 104.228(5) | 123.5980(10) |
| γ/° | 91.123(5) | 90 |
| Volume/Å^3^ | 1829.09(19) | 24063.7(5) |
| Z | 2 | 4 |
| ρ_calc_g/cm^3^ | 1.084 | 1.176 |
| μ/mm^‑1^ | 0.487 | 1.399 |
| F(000) | 644.0 | 8980.0 |
| Colour/shape | Colourless/plate | Blue/plate |
| Crystal size/mm^3^ | 0.34 × 0.293 × 0.22 | 0.2 × 0.167 × 0.13 |
| Radiation | CuKα (λ = 1.54186) | GaKα (λ = 1.34143) |
| 2Θ range for data collection/° | 9.192 to 141.166 | 8.182 to 111.976 |
| Index ranges | -12 ≤ h ≤ 9, -15 ≤ k ≤ 15, -12 ≤ l ≤ 17 | -49 ≤ h ≤ 47, -30 ≤ k ≤ 39, -27 ≤ l ≤ 25 |
| Reflections collected | 37965 | 177075 |
| Independent reflections | 6797 [R_int_ = 0.0499, R_sigma_ = 0.0216] | 23584 [R_int_ = 0.0682, R_sigma_ = 0.0268] |
| Data/restraints/parameters | 6797/141/449 | 23584/2107/1511 |
| Goodness-of-fit on F^2^ | 1.045 | 1.216 |
| Final R indexes [I>=2σ (I)] | R_1_ = 0.0858, wR_2_ = 0.1797 | R_1_ = 0.0988, wR_2_ = 0.2147 |
| Final R indexes [all data] | R_1_ = 0.0953, wR_2_ = 0.1852 | R_1_ = 0.1233, wR_2_ = 0.2269 |
| Largest diff. peak/hole / e Å^-3^ | 0.39/-0.31 | 1.32/-0.57 |

Table S2: Crystallographic data for **[Het-Rh_2_]*^t^*^Bu^** and **[Het-Rh_2_]^Me^*.***

| **Compound** | **[Het-Rh_2_]*^t^*^Bu^** | **[Het-Rh_2_]^Me^** |
| --- | --- | --- |
| CCDC number | 2522555 | 2522558 |
| Empirical formula | C_142_H_160_B_2_F_8_N_4_O_4_P_4_Rh_2_ | C_114_H_100_B_2_Cl_8_F_8_N_4_O_2_P_4_Rh_2_ |
| Formula weight | 2490.05 | 2344.89 |
| Temperature/K | 150 | 150 |
| Crystal system | triclinic | triclinic |
| Space group | *P*$\bar{1}$ | *P*$\bar{1}$ |
| a/Å | 10.5700(2) | 10.7006(4) |
| b/Å | 13.8007(2) | 13.1509(6) |
| c/Å | 23.0582(4) | 19.7784(9) |
| α/° | 99.3320(10) | 90.224(3) |
| β/° | 94.2540(10) | 98.804(3) |
| γ/° | 104.3760(10) | 99.388(3) |
| Volume/Å^3^ | 3192.01(10) | 2712.4(2) |
| Z | 1 | 1 |
| ρ_calc_g/cm^3^ | 1.295 | 1.436 |
| μ/mm^‑1^ | 2.073 | 3.592 |
| F(000) | 1304.0 | 1196.0 |
| Colour/shape | Green/plate | Green/needle |
| Crystal size/mm^3^ | 0.11 × 0.053 × 0.01 | 0.5 × 0.21 × 0.05 |
| Radiation | GaKα (λ = 1.34143) | GaKα (λ = 1.34143) |
| 2Θ range for data collection/° | 5.854 to 111.308 | 7.8 to 113.812 |
| Index ranges | -12 ≤ h ≤ 12, -15 ≤ k ≤ 16, -28 ≤ l ≤ 19 | -12 ≤ h ≤ 13, -10 ≤ k ≤ 16, -24 ≤ l ≤ 24 |
| Reflections collected | 55512 | 44313 |
| Independent reflections | 12157 [R_int_ = 0.0219, R_sigma_ = 0.0178] | 10866 [R_int_ = 0.1484, R_sigma_ = 0.1286] |
| Data/restraints/parameters | 12157/600/841 | 10866/21/653 |
| Goodness-of-fit on F^2^ | 1.041 | 1.017 |
| Final R indexes [I>=2σ (I)] | R_1_ = 0.0295, wR_2_ = 0.0743 | R_1_ = 0.0841, wR_2_ = 0.1685 |
| Final R indexes [all data] | R_1_ = 0.0336, wR_2_ = 0.0771 | R_1_ = 0.1394, wR_2_ = 0.1947 |
| Largest diff. peak/hole / e Å^-3^ | 0.38/-0.77 | 2.06/-1.81 |

Table S3: Crystallographic data for **[Het-Rh_2_]^tBu2^**

| **Compound** | **[Het-Rh_2_]*^t^*^Bu2^** |
| --- | --- |
| CCDC number | 2522556 |
| Empirical formula | C_212_H_264_B_2_F_8_N_4_O_7.5_P_4_Rh_2_ |
| Formula weight | 3491.58 |
| Temperature/K | 150 |
| Crystal system | monoclinic |
| Space group | *P*2_1_/*c* |
| a/Å | 20.2259(4) |
| b/Å | 19.4967(6) |
| c/Å | 25.4877(5) |
| α/° | 90 |
| β/° | 104.665(2) |
| γ/° | 90 |
| Volume/Å^3^ | 9723.3(4) |
| Z | 2 |
| ρ_calc_g/cm^3^ | 1.1933 |
| μ/mm^‑1^ | 2.188 |
| F(000) | 3712.0 |
| Colour/shape | Blue/plate |
| Crystal size/mm^3^ | 0.8 × 0.6 × 0.2 |
| Radiation | Cu Kα (λ = 1.54186) |
| 2Θ range for data collection/° | 10.116 to 150.342 |
| Index ranges | -23 ≤ h ≤ 25, -22 ≤ k ≤ 24, -31 ≤ l ≤ 16 |
| Reflections collected | 105264 |
| Independent reflections | 19805 [R_int_ = 0.0679, R_sigma_ = 0.0527] |
| Data/restraints/parameters | 19805/1386/1173 |
| Goodness-of-fit on F^2^ | 0.838 |
| Final R indexes [I>=2σ (I)] | R_1_ = 0.0523, wR_2_ = 0.1259 |
| Final R indexes [all data] | R_1_ = 0.0835, wR_2_ = 0.1515 |
| Largest diff. peak/hole / e Å^-3^ | 0.66/-0.39 |


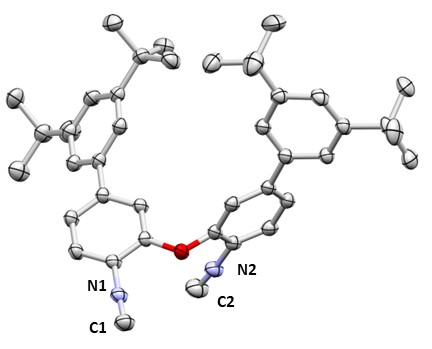


Figure S61: Molecular structure of **L^tBu^** in the solid state. One of the four t-butyl group is disordered. Only one of the disordered parts is shown. Thermal ellipsoids are drawn at 50 % probability. Selected bond distances (Å): N1-C1: 1.152(4) Å, N2-C2: 1.155(4) Å.


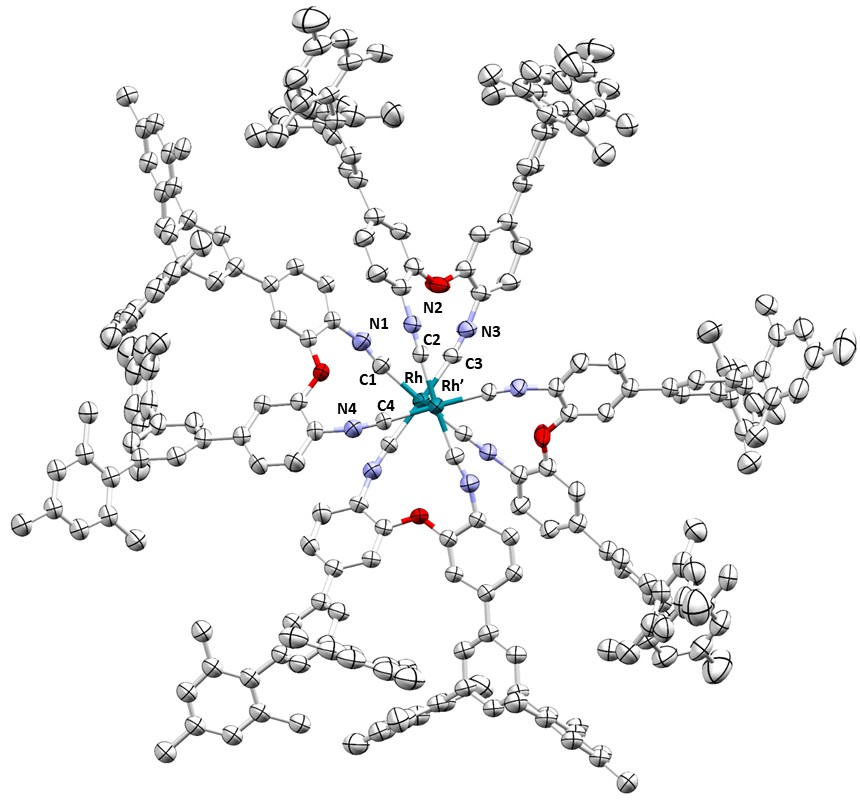


Figure S62: Molecular structure of **[Hom-Rh_2_]^Mes^** in the solid state. Non-coordinating solvents, hydrogen atoms and counter-anions are omitted for clarity. Twelve of the sixteen mesityl groups and four phenyl rings of 2,2^’^-oxydianiline are disordered. Only one of the disordered parts is shown. Thermal ellipsoids are drawn at 50 % probability. Selected bond distances (Å): Rh···Rh’: 3.2561(11) Å, Rh-C1: 1.960(5) Å, Rh’-C2: 1.989(5) Å, Rh-C3: 1.963(5) Å, Rh’-C4: 1.988(4) Å, N1-C1: 1.137(6) Å, N2-C2: 1.099(5) Å, N3-C3: 1.124(5) Å, N4-C4: 1.133(6) Å.


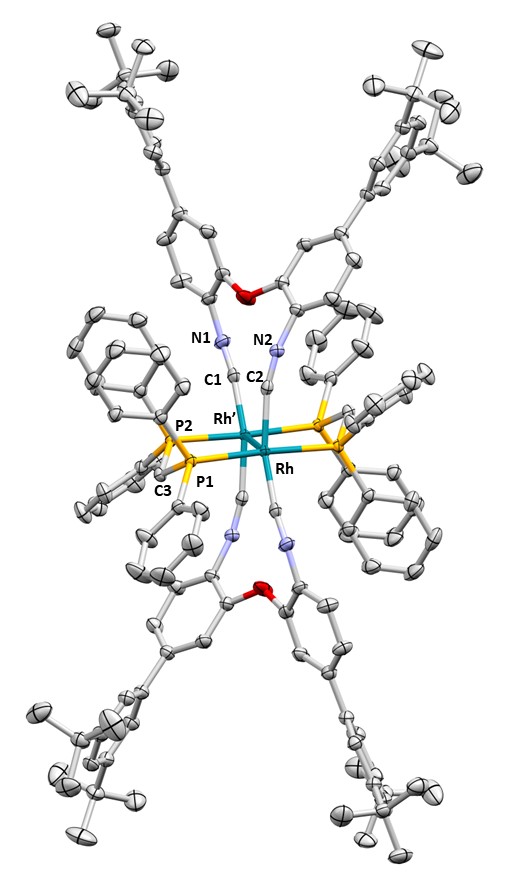


Figure S63: Molecular structure of **[Het-Rh_2_]^tBu^** in the solid state. Non-coordinating solvents, hydrogen atoms and counter-anions are omitted for clarity. Six of the eight t-butyl groups and BF_4_ anions are disordered. Only one of the disordered parts is shown. Thermal ellipsoids are drawn at 50 % probability. Selected bond distances (Å): Rh···Rh’: 3.1409(2) Å, Rh-P1: 2.3225(4) Å, Rh-P2: 2.3284(4) Å, Rh-C2: 1.913(2) Å, Rh’-C1: 1.954(2) Å, N1-C1: 1.173(2) Å, N2-C2: 1.168(2) Å, P1-C3: 1.835(2) Å, P2-C3: 1.836(2) Å; Selected bond angles (°): C1’-Rh-C2: 161.80(8), C2-Rh-Rh’: 115.74(6), P1-C3-P2: 115.94(9).


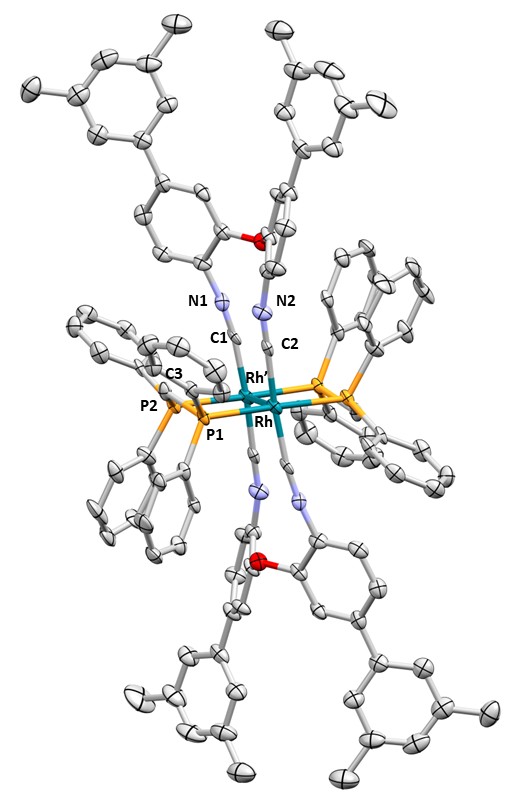


Figure S64: Molecular structure of **[Het-Rh_2_]^Me^** in the solid state. Non-coordinating solvents, hydrogen atoms and counter-anions are omitted for clarity. Thermal ellipsoids are drawn at 50 % probability. Selected bond distances (Å): Rh···Rh’: 3.0043(10) Å, Rh-P1: 2.316(2) Å, Rh-P2: 2.331(2) Å, Rh-C2: 1.918(7) Å, Rh’-C1: 1.927(7) Å, N1-C1: 1.177(9) Å, N2-C2: 1.190(10) Å, P1-C3: 1.860(7) Å, P2-C3: 1.845(7) Å; Selected bond angles (°): C1’-Rh-C2: 172.60(3), C2-Rh-Rh’: 97.70(2), P1-C3-P2: 113.9(4).


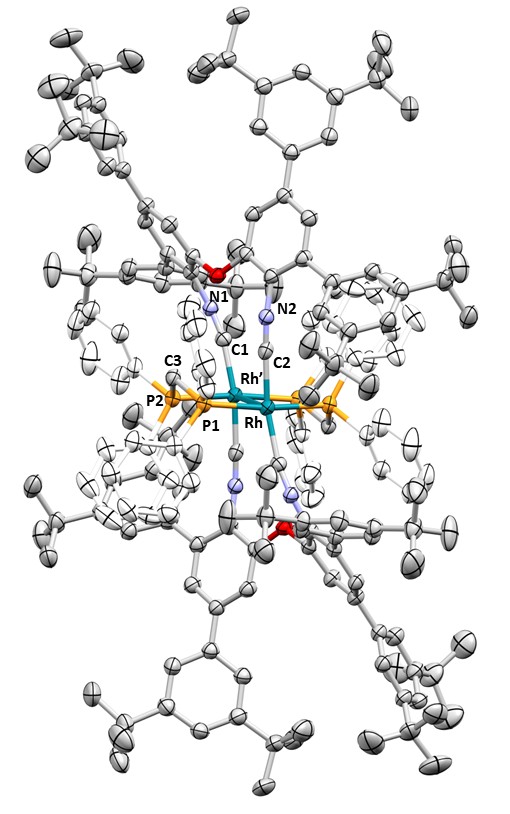


Figure S65: Molecular structure of **[Het-Rh_2_]^tBu2^** in the solid state. Non-coordinating solvents, hydrogen atoms and counter-anions are omitted for clarity. Eight of the sixteen t-butyl groups and BF_4_ anions are disordered. Only one of the disordered parts is shown. Thermal ellipsoids are drawn at 50 % probability. The phenyl groups attached to the phosphine moiety of **[Het-Rh_2_]^tBu2^** are colored white for visual clarity. Selected bond distances (Å): Rh···Rh’: 3.2753(5) Å, Rh-P1: 2.3299(8) Å, Rh-P2: 2.3408(8) Å, Rh-C2: 1.927(4) Å, Rh’-C1: 1.958(3) Å, N1-C1: 1.156(4) Å, N2-C2: 1.156(4) Å, P1-C3: 1.840(5) Å, P2-C3: 1.839(3) Å; Selected bond angles (°): C1’-Rh-C2: 164.52(2), C2-Rh-Rh’: 86.24(10), P1-C3-P2: 116.3(2).


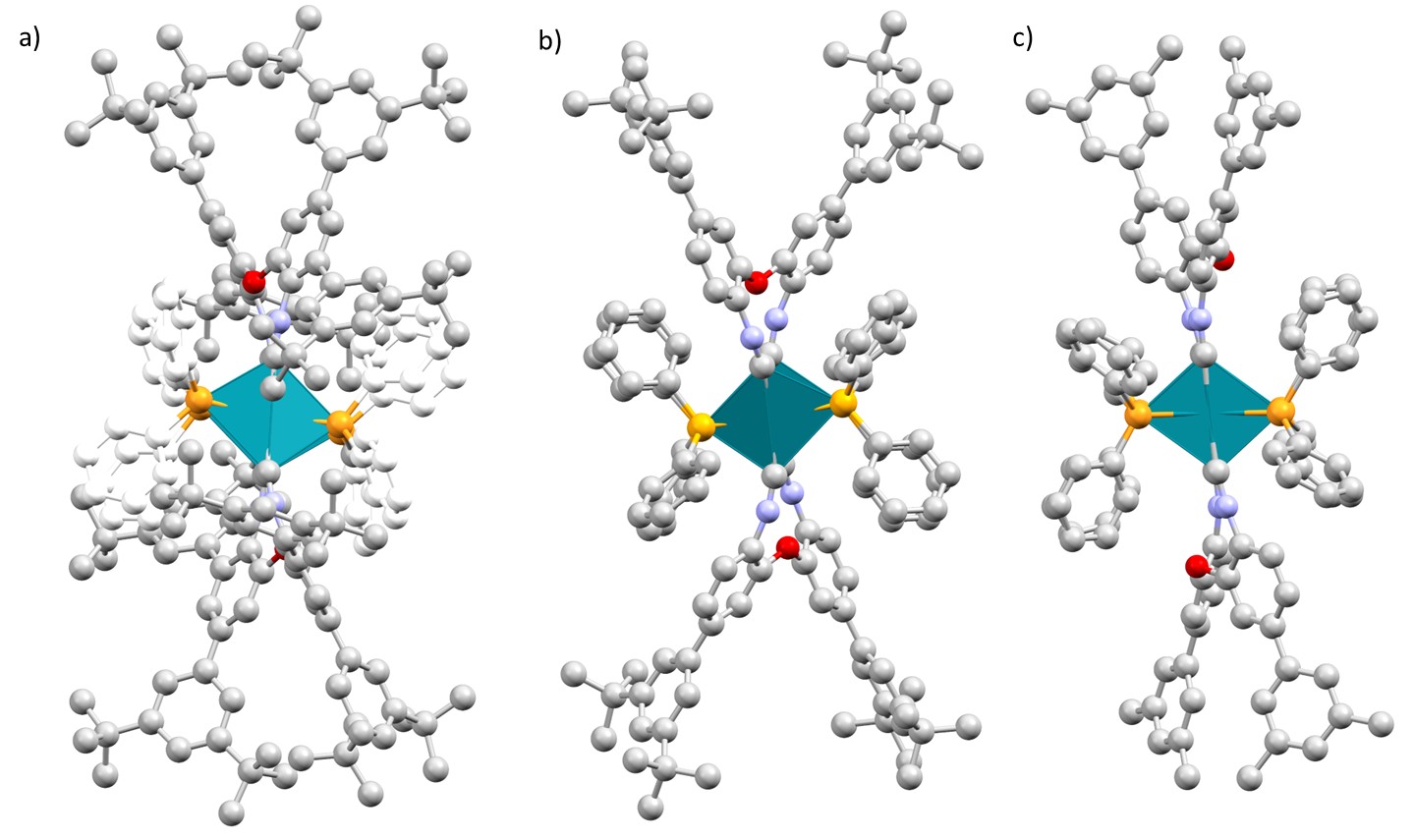


Figure S66: Eclipsed conformation at the Rh_2_ core of a) **[Het-Rh_2_]^tBu2^**, b) **[Het-Rh_2_]^tBu^** and c) **[Het-Rh_2_]^Me^**.

# **UV-Vis steady state absorption spectroscopy**


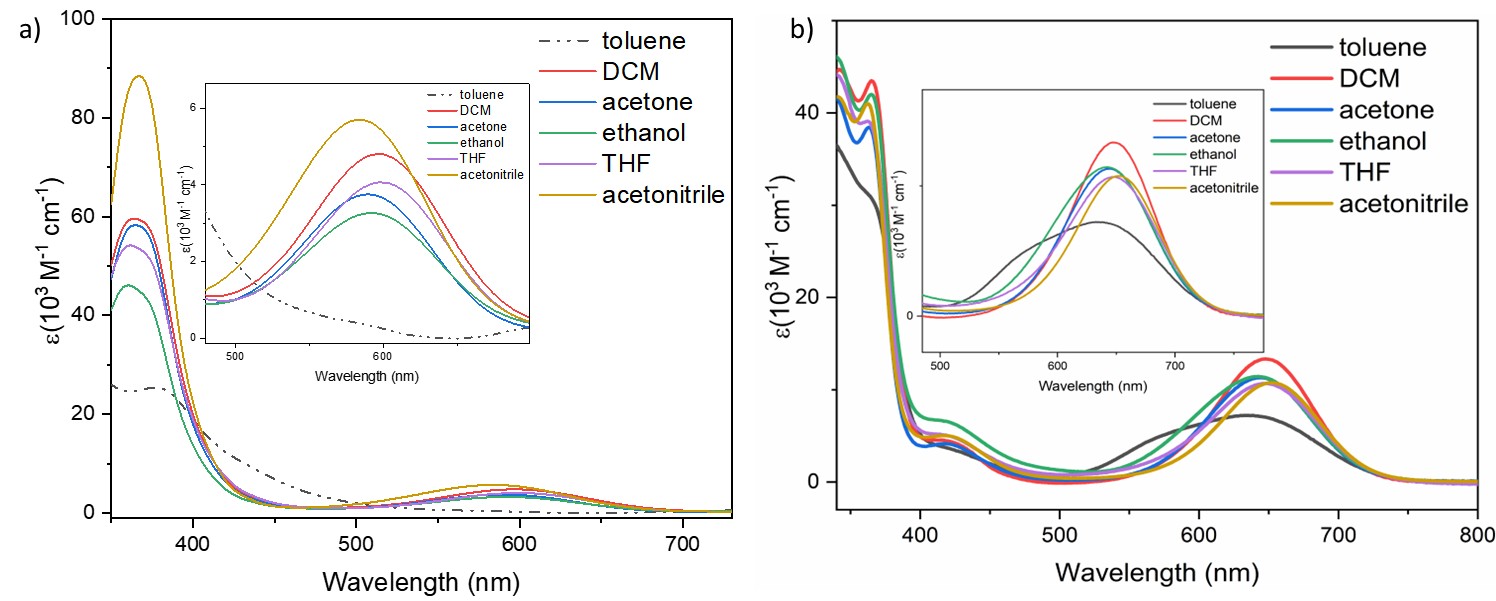


Figure S67: UV-Vis absorption spectra of a) **[Hom-Rh_2_]^tBu^** and b) **[Het-Rh_2_]^tBu^** in different solvents at room temperature.

The solvent dependence trend is opposite to what is normally observed for MLCT absorption bands, which may be due to predominant dσ*→ pσ character rather than MMLCT character.


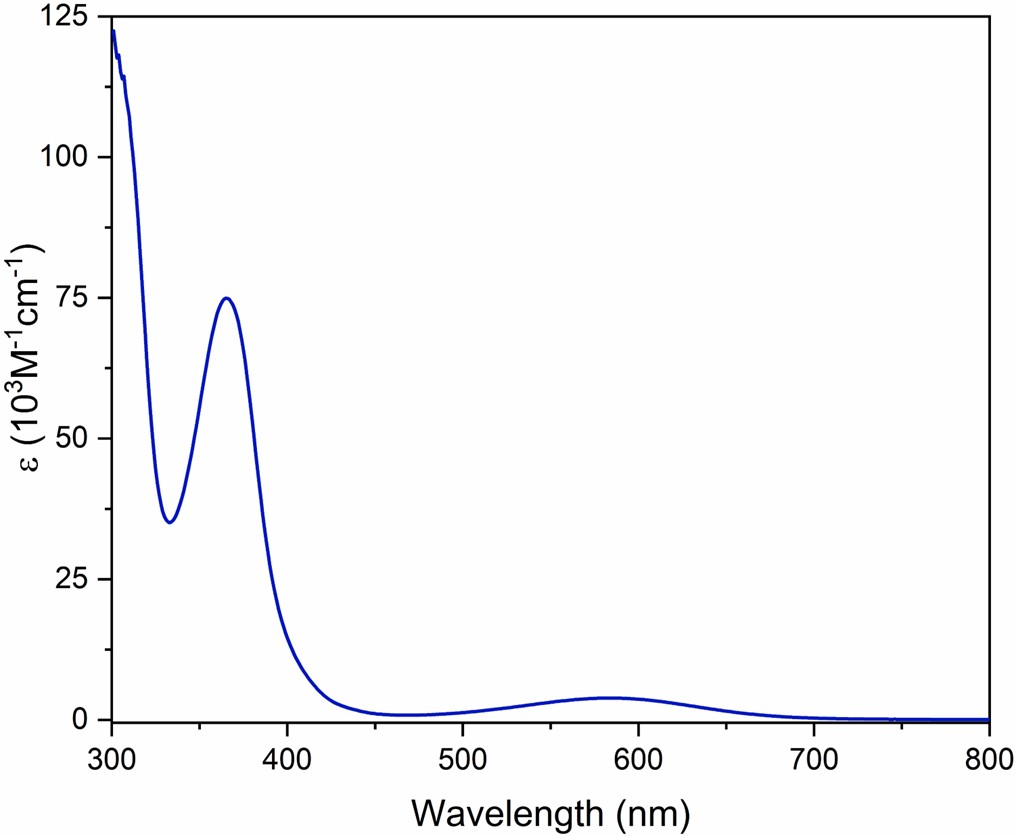


Figure S68: UV-Vis absorption spectrum of **[Hom-Rh_2_]^Mes^** in acetonitrile.

# **Photoluminescence spectroscopy**


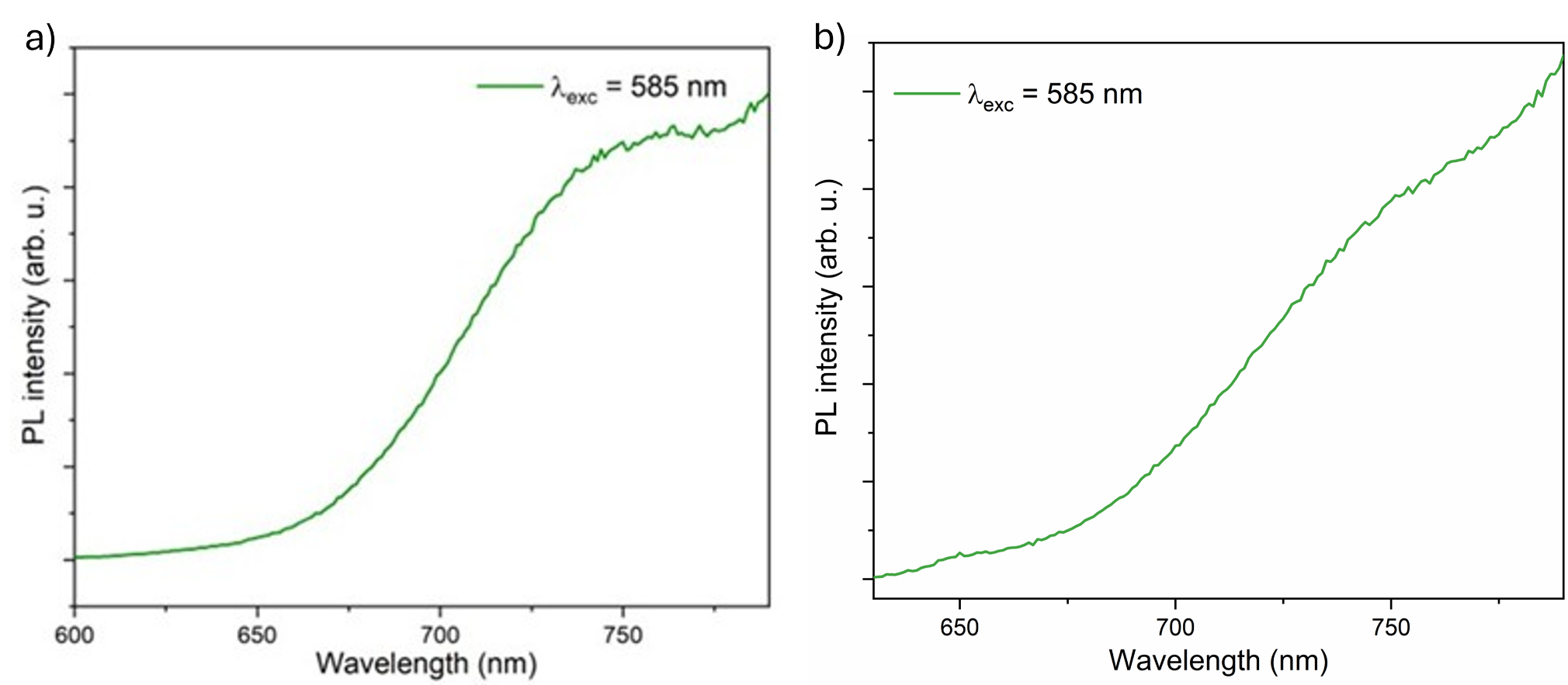


Figure S69: Steady-state photoluminescence spectrum of a) **[Hom-Rh_2_]^tBu^** and b) **[Hom-Rh_2_]^Mes^** showing weak fluorescence in deoxygenated CH_2_Cl_2_ after excitation at 585 nm. The shoulder is clearly visible only when the spectrum is collected until ~790 nm.


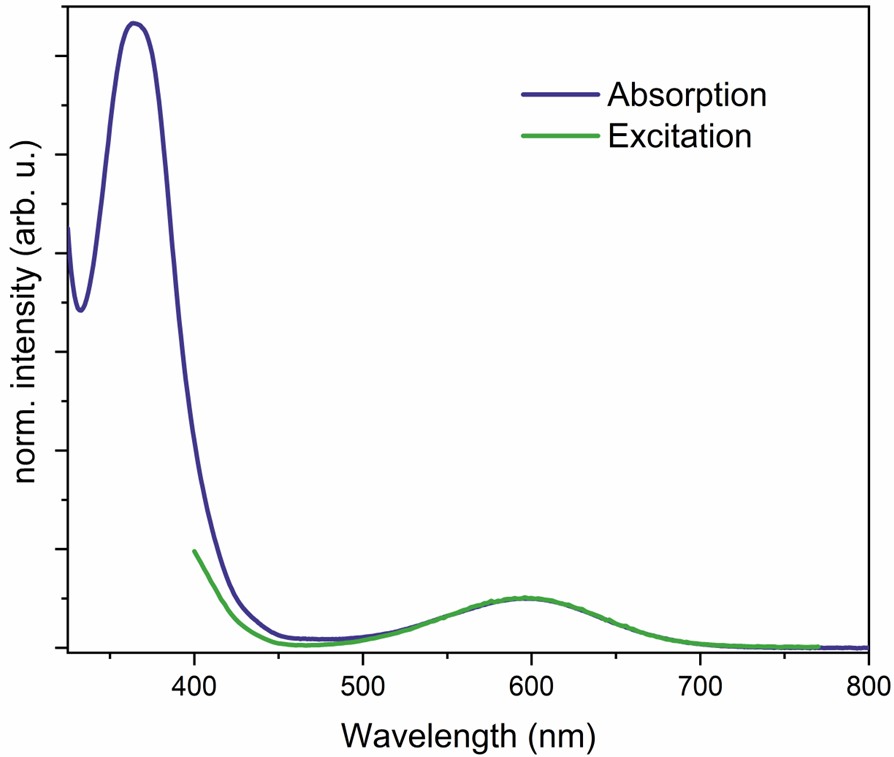


Figure S70: Excitation spectrum of **[Het-Rh_2_]^tBu^** (λ_em_ = 780 nm) overlaid with its respective absorption spectrum in deoxygenated CH_2_Cl_2_.


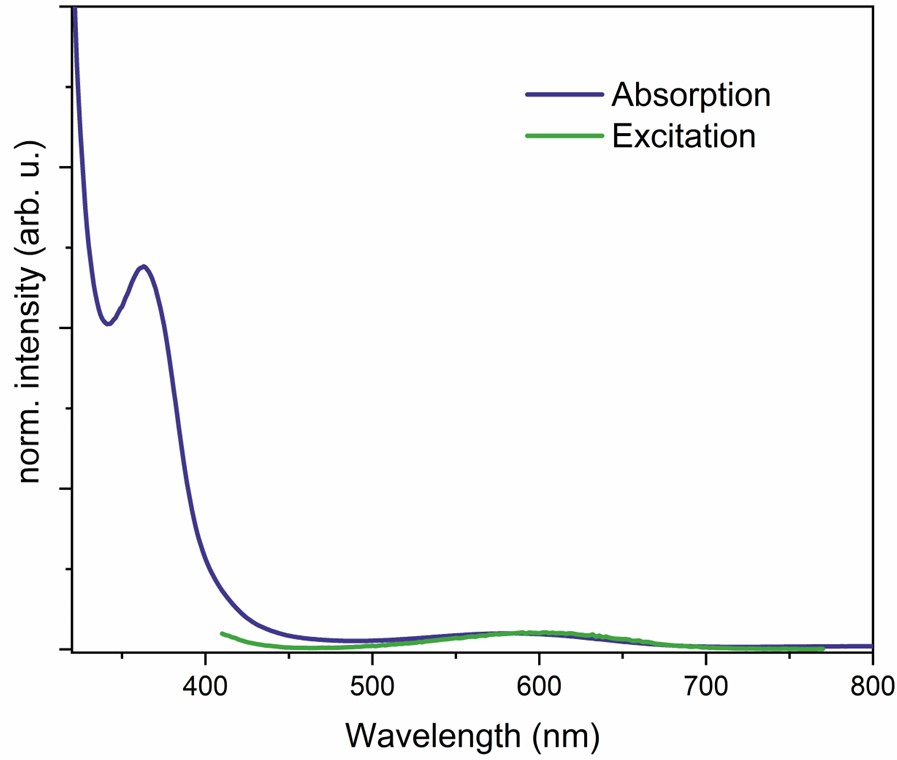


Figure S71: Excitation spectrum of **[Hom-Rh_2_]^Mes^** (λ_em_ = 780 nm) overlaid with its respective absorption spectrum in deoxygenated CH_2_Cl_2_.


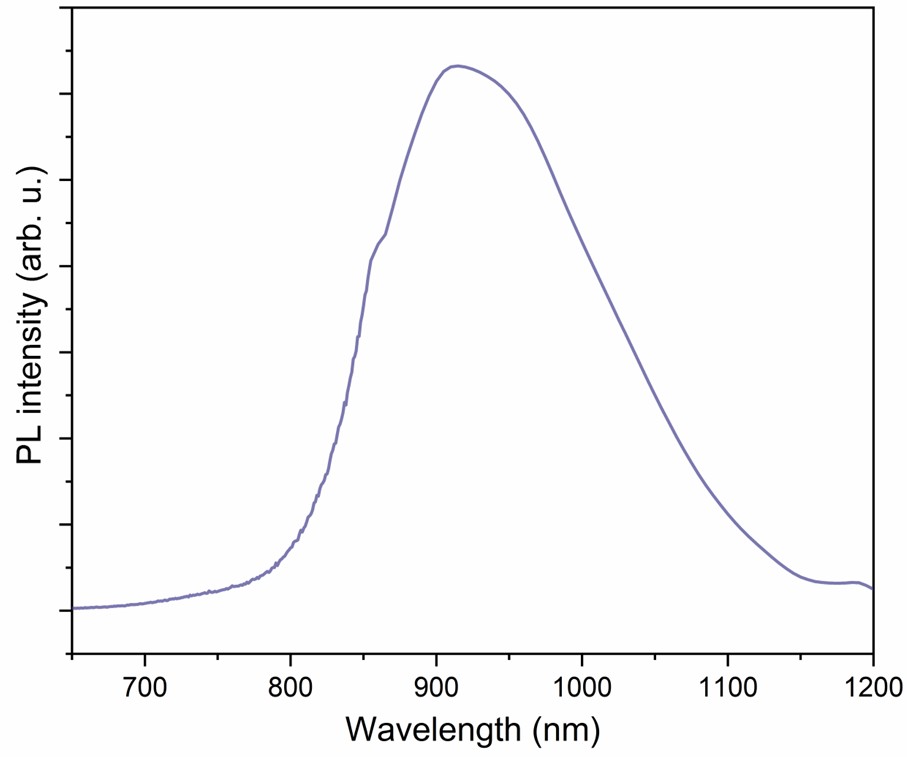


Figure S72: Emission spectra of **[Hom-Rh_2_]^Mes^** (λ_ex_= 620 nm) in deoxygenated CH_2_Cl_2_.


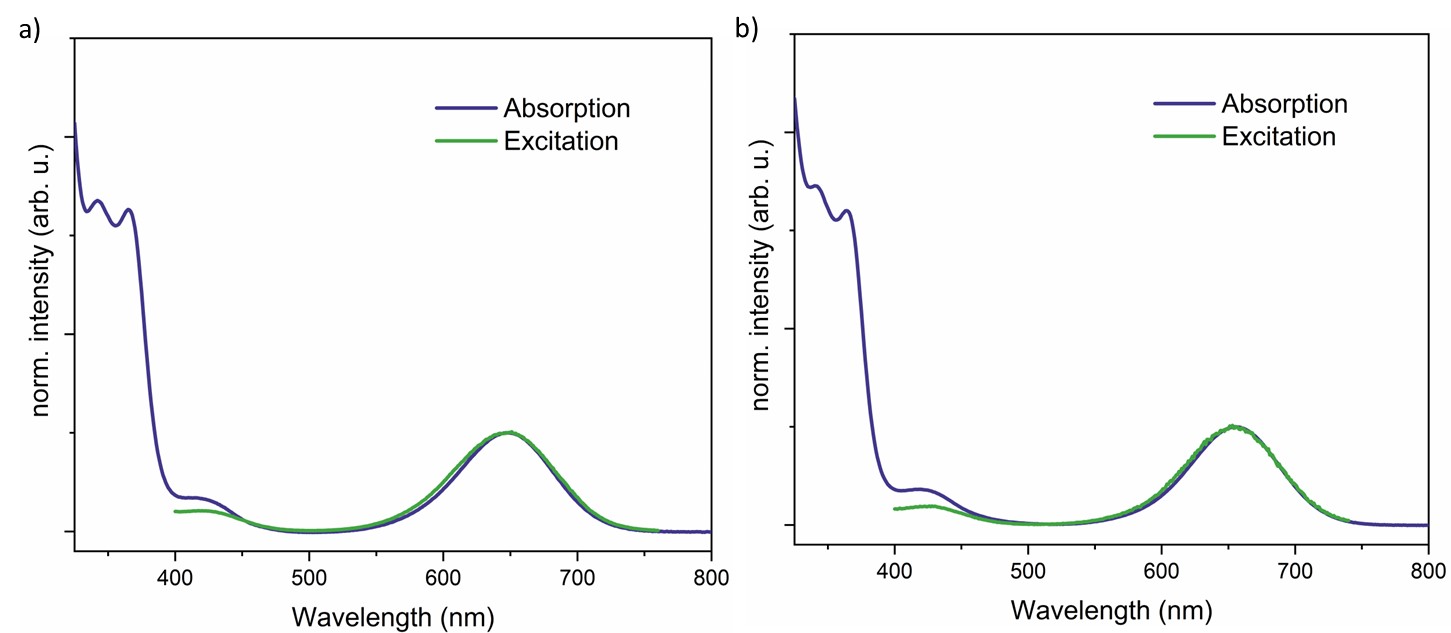


Figure S73: Excitation spectra of a) **[Het-Rh_2_]^tBu^** (λ_em_ = 780 nm) and b) **[Het-Rh_2_]^Me^** (λ_em_ = 760 nm) overlaid with their respective absorption spectrum in deoxygenated CH_2_Cl_2_.


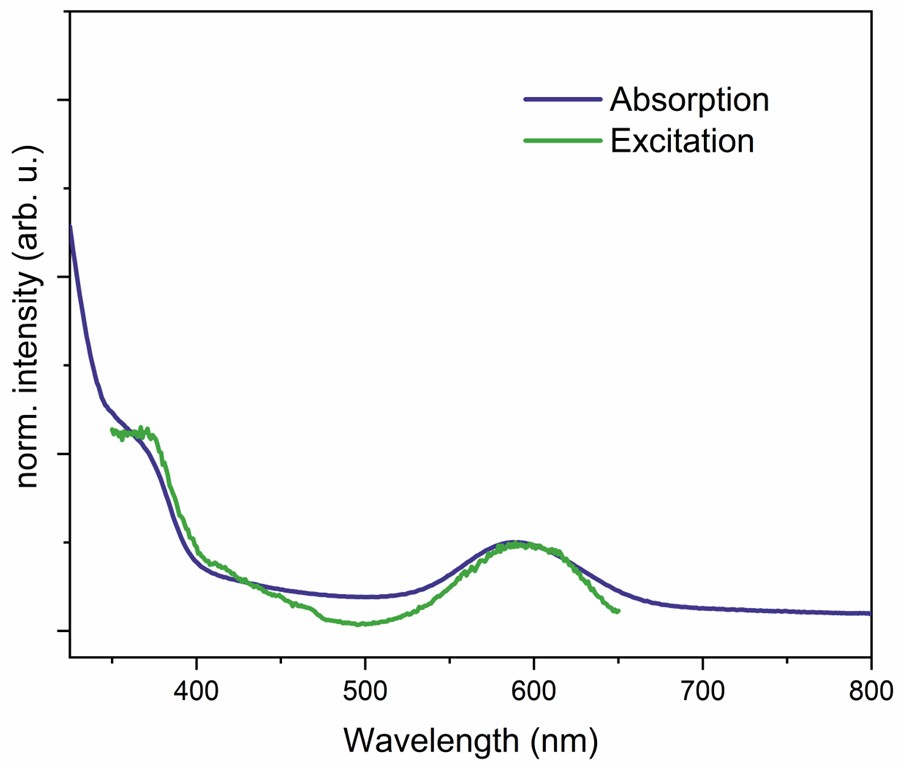


Figure S74: Excitation spectrum of **[Het-Rh_2_]^tBu2^** (λ_em_ = 670 nm) overlaid with its absorption spectrum in deoxygenated CH_3_CN.


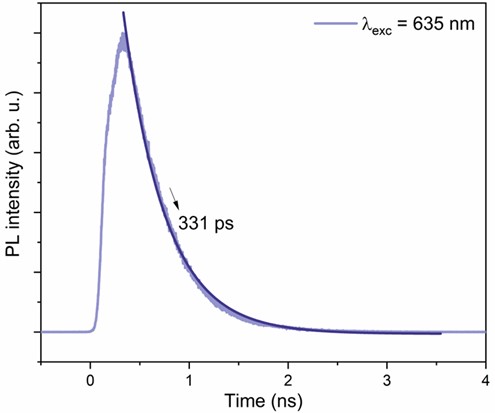


Figure S75: Fluorescence decay (λ_exc_ = 635 nm) of **[Het-Rh_2_]^Me^** detected at λ_obs_ = 725 nm obtained from TCSPC measurements. The dark purple trace is the result of a single-exponential fit to the experimental data.

**Determination of the energy gap between the lowest singlet and the lowest triplet excited states:**

The S_1_ energy was estimated from the intersection point of the normalized low-energy absorption band and the normalized fluorescence spectrum at room temperature. The T_1_ energy was estimated based on the wavelength at which the 77 K phosphorescence spectrum reaches 10% of its maximum intensity. The phosphorescence maximum of [Het-Rh_2_]*^t^*^Bu2^ falls between 800-900 nm, where both the PMT and NIR detector have low sensitivity. Hence, the T_1_ energy of this complex cannot be determined with meaningful accuracy. Due to very weak fluorescence in the homoleptic complexes, the S_1_ and T_1_ energies were not determined in these cases.


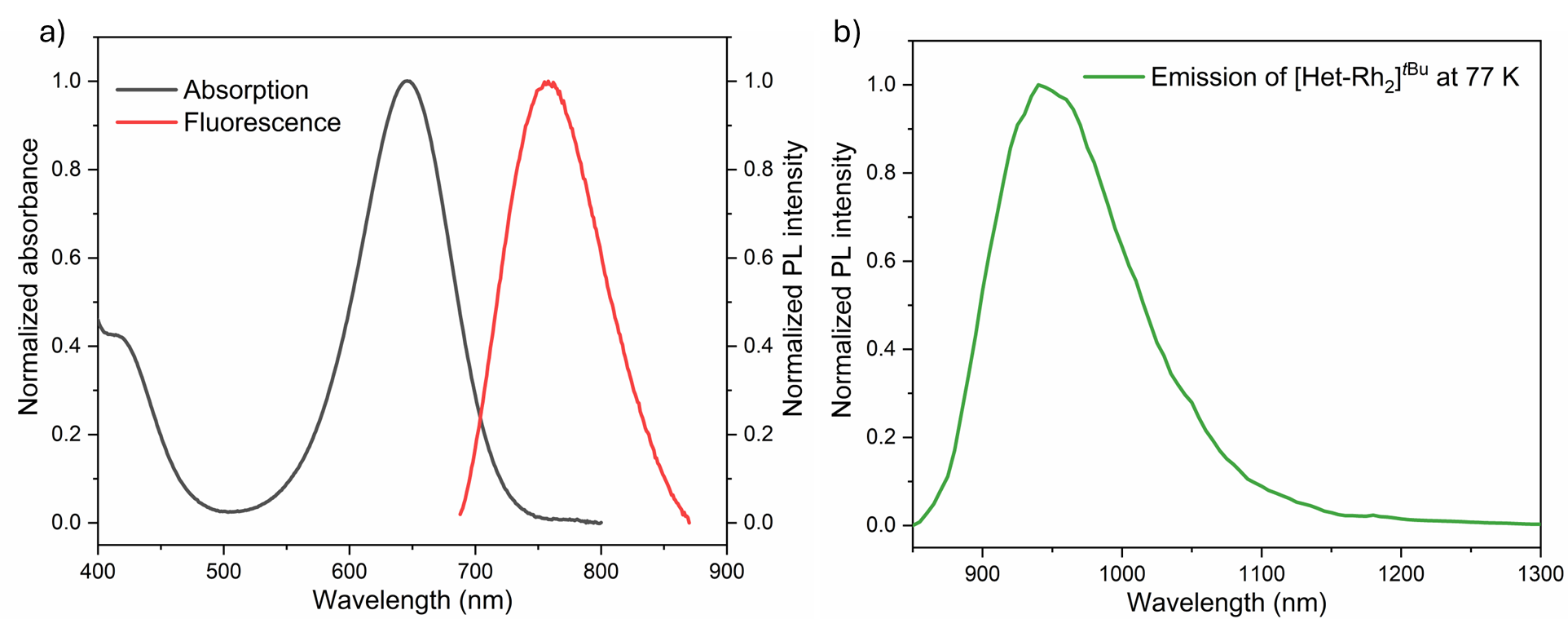


Figure S76: a) Normalized absorption and fluorescence spectra of **[Het-Rh_2_]^tBu^** in DCM:MeOH (1:1) mixture at room temperature; b) Phosphorescence spectrum of **[Het-Rh_2_]^tBu^** in frozen glassy matrix [DCM:MeOH (1:1)] at 77 K. E_S1_ = 1.76 eV, E_T1_ = 1.42 eV, ΔE_ST_ = 0.34 eV. Excitation occurred at 675 nm.


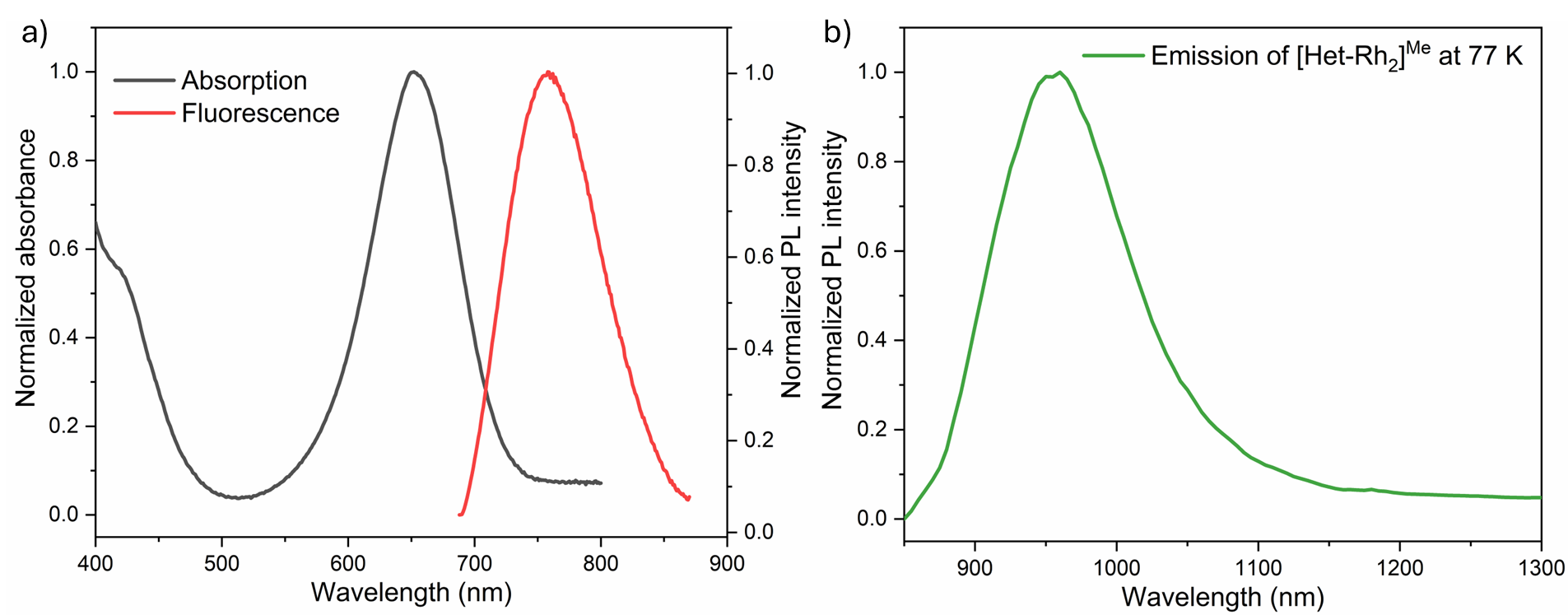


Figure S77: a) Normalized absorption and fluorescence spectra of **[Het-Rh_2_]^Me^** in DCM:MeOH (1:1) mixture at room temperature; b) Phosphorescence spectrum of **[Het-Rh_2_]^Me^** in frozen glassy matrix [DCM:MeOH (1:1)] at 77 K. E_S1_ = 1.75 eV, E_T1_ = 1.42 eV, ΔE_ST_ = 0.33 eV. Excitation occurred at 675 nm.

**Temperature-dependent luminescence measurements**

Temperature-dependent luminescence spectra (from room temperature down to 180 K to avoid freezing of CH_2_Cl_2_) were recorded using the Fluorolog setup described in the materials and methods section. An Oxford Optistat DN bath cryostat was mounted into the setup and cooled with liquid nitrogen. The sample solution was filled in a standard 1 x 1 cm cuvette and mounted in the cryostat. The emission was measured perpendicular to the excitation beam. Fluorescence spectra between 688 and 870 nm were recorded using a water-cooled photomultiplier tube (PMT, Hamamatsu R928), while phosphorescence spectra were measured using a DSS-LN solid-state detector. Owing to the weak sensitivity of the NIR detector below 890 nm, data recorded between 870 and 890 nm exhibit increased noise and were excluded from quantitative comparison across temperatures.


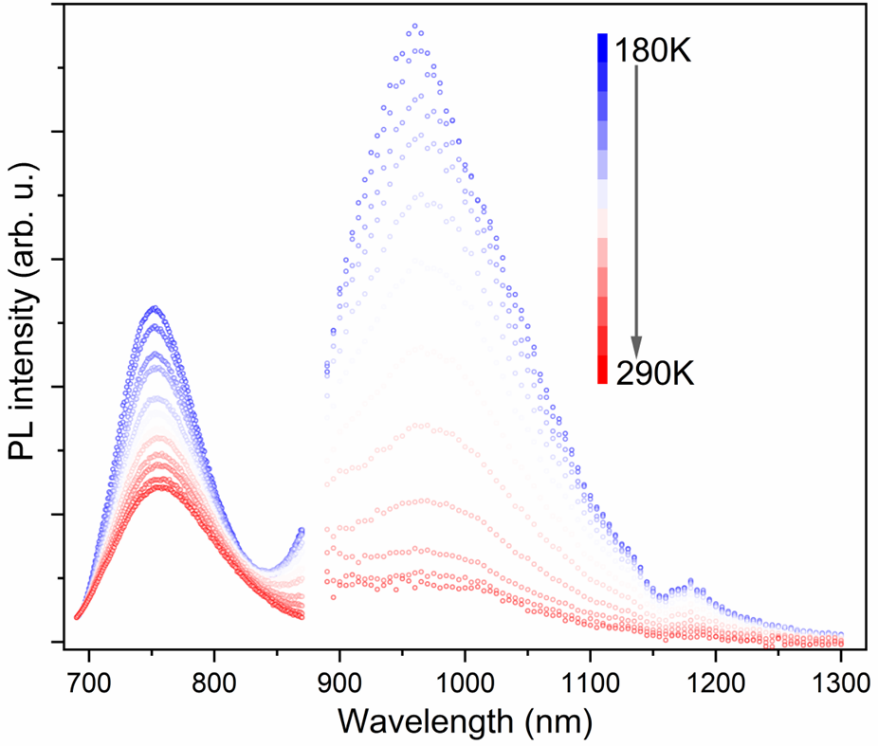


Figure S78: Temperature-dependent photoluminescence spectra of **[Het-Rh_2_]^tBu^** (λ_ex_ = 670 nm) in deoxygenated CH_2_Cl_2_.





Figure S79: Temperature-dependent photoluminescence spectra of **[Het-Rh_2_]^Me^** (λ_ex_ = 670 nm) in deoxygenated CH_2_Cl_2_.

Phosphorescence is significantly enhanced upon lowering the temperature, indicating suppressed non-radiative decay pathways. A 9-fold and 5-fold increase in phosphorescence to fluorescence ratio was observed for **[Het-Rh_2_]*^t^*^Bu^** and **[Het-Rh_2_]^Me^**, respectively, when the emission was measured at 180 K.

# **Luminescence quantum yield determination**

The luminescence quantum yield of the complexes in deaerated solutions of dichloromethane was determined relative to that of [Os(bpy)_3_]^2+^ (*ϕ*_MeCN_ = 0.5 %) in deaerated acetonitrile at 20 °C.^[5]^ Equation S1 was used for the photoluminescence quantum yield determination, where S is the slope of the plot of the integrated emission intensity vs the absorbance at the indicated excitation wavelength and $\eta$ is the refractive index of the solvent ($\eta$_acetonitrile_ = 1.344, $\eta$_dichloromethane_ = 1.424).

$\phi_{complex}= \frac{S_{complex}}{S_{[Os{(bpy)}_{3}]^{2+}}}\times\frac{\eta_{dichloromethane}^{2}}{\eta_{acetonitrile}^{2}}\times\phi_{[Os(bpy)_{3}]^{2+}}$ Eq S1

Since the reference and the complexes emit in different spectral ranges, the emission spectra were converted to wavenumbers:

Specifically,

- The x-axis was converted from wavelength λ (nm) to wavenumber ν̃ (cm^-1^) using the following equation

$\nũ (\mathrm{cm}^{-1})=\frac{{10}^{7}}{\lambda(nm)}$ Eq S2

- The y-axis was corrected by multiplying the square of the corresponding wavelength with the intensity of the emission

$I(\nũ)= \lambda^{2}I(\lambda)$ Eq S3

Due to very weak fluorescence for **[Hom-Rh_2_]*^t^*^Bu^** (Figure S69a) and **[Hom-Rh_2_]^Mes^** (Figure S69b), Gaussian deconvolution was not feasible in these specific cases. Consequently, the upper limit of the fluorescence quantum yield for this compound was estimated by comparing the relative intensities of phosphorescence and fluorescence emission bands.

Additionally, because all complexes except **[Het-Rh_2_]*^t^*^Bu2^**, including the reference emit in both the visible and NIR region (requiring two different detectors), a full emission spectrum was only recorded for the first data point. For the remaining points, emission was measured until 855 nm and the rest of the spectrum was reconstructed.

Since heteroleptic complexes **[Het-Rh_2_]*^t^*^Bu^** and **[Het-Rh_2_]^Me^** display two emission bands, their spectra were deconvoluted into two peaks using Gaussian fitting in Origin software. The integrated area of each fitted peak was then used to calculate the slope.


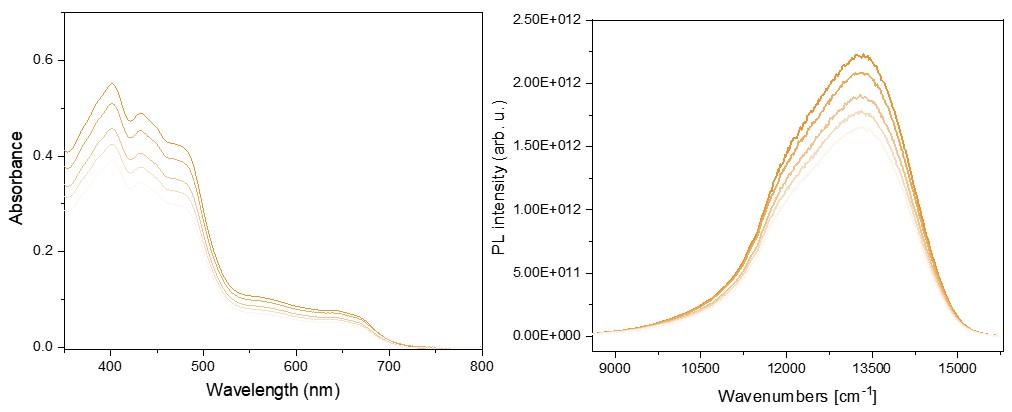

Figure S80: Raw data for using [Os(bpy)_3_]^2+^ (in deareated acetonitrile) as a reference for luminescence quantum yield determination at the excitation wavelength of 610 nm.

Figure S81: Raw data for luminescence quantum yield determination of **[Hom-Rh_2_]^tBu^** in deaerated dichloromethane at the excitation wavelength of 610 nm.

Figure S82: Top and middle: Raw data for luminescence quantum yield determination of **[Het-Rh_2_]^tBu^** in deaerated dichloromethane at the excitation wavelength of 610 nm. Bottom: Emission spectrum fitted by deconvolution into two Gaussian peaks, along with the cumulative fit overlaid on the experimental spectrum.

Figure S83: Top and middle: Raw data for luminescence quantum yield determination of **[Het-Rh_2_]^Me^** in deaerated dichloromethane at the excitation wavelength of 610 nm. Bottom: Emission spectrum fitted by deconvolution into two Gaussian peaks, along with the cumulative fit overlaid on the experimental spectrum.

Figure S84: Raw data for luminescence quantum yield determination of **[Het-Rh_2_]^tBu2^** in deaerated dichloromethane at the excitation wavelength of 585 nm.

Figure S85: Raw data for using [Os(bpy)_3_]^2+^ (in deareated acetonitrile) as a reference for luminescence quantum yield determination at the excitation wavelength of 585 nm.


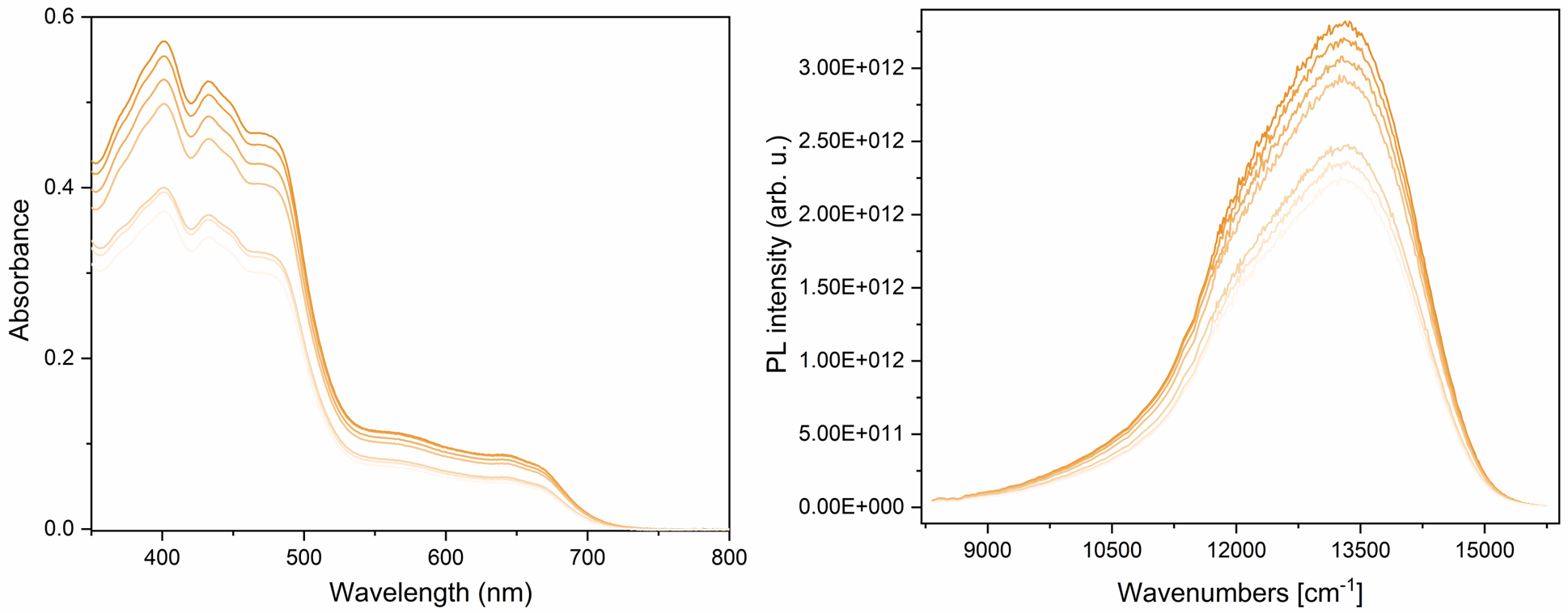


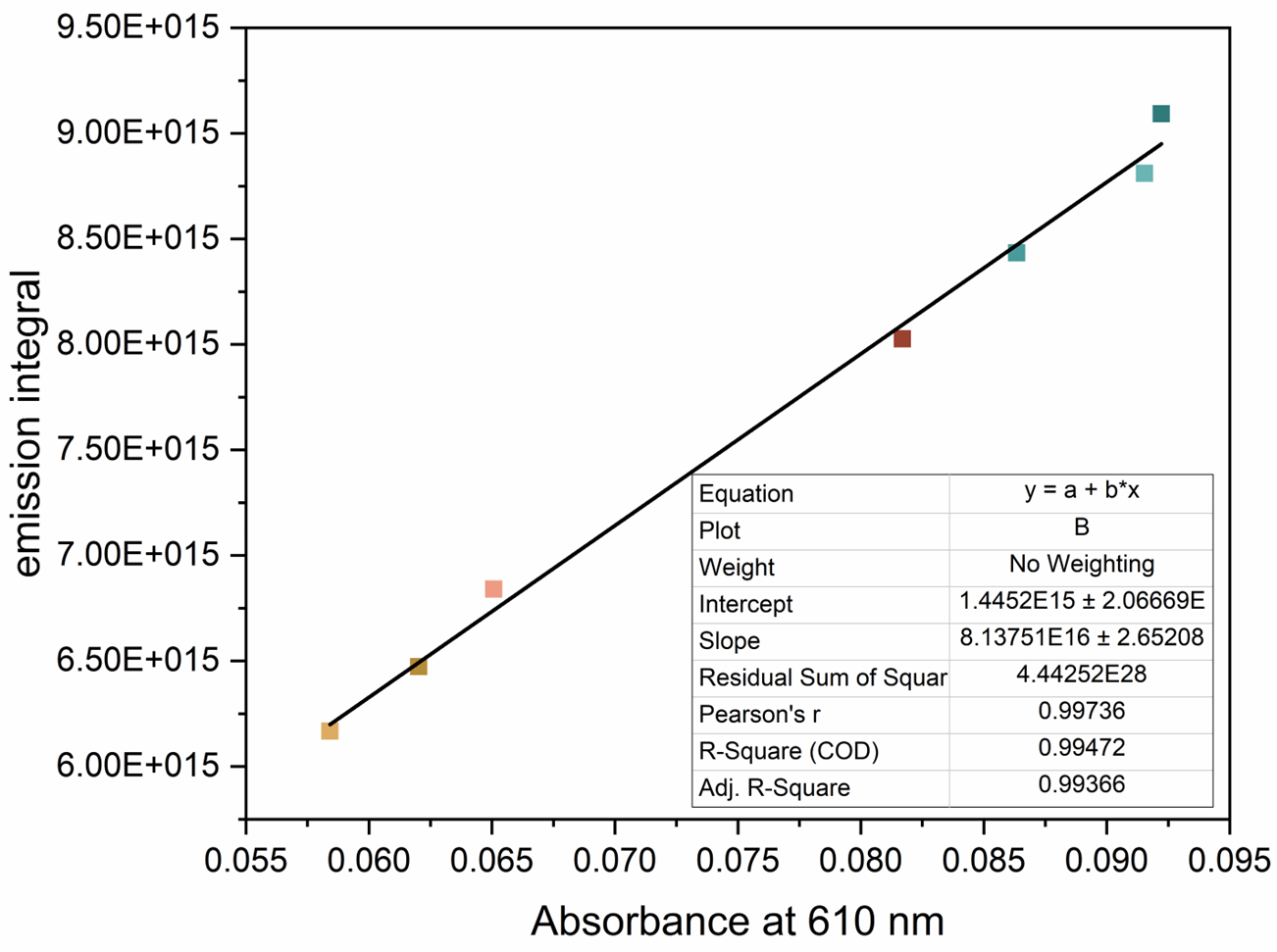


Figure S86: Raw data for using [Os(bpy)_3_]^2+^ (in deareated acetonitrile) as a reference for luminescence quantum yield determination at the excitation wavelength of 610 nm.


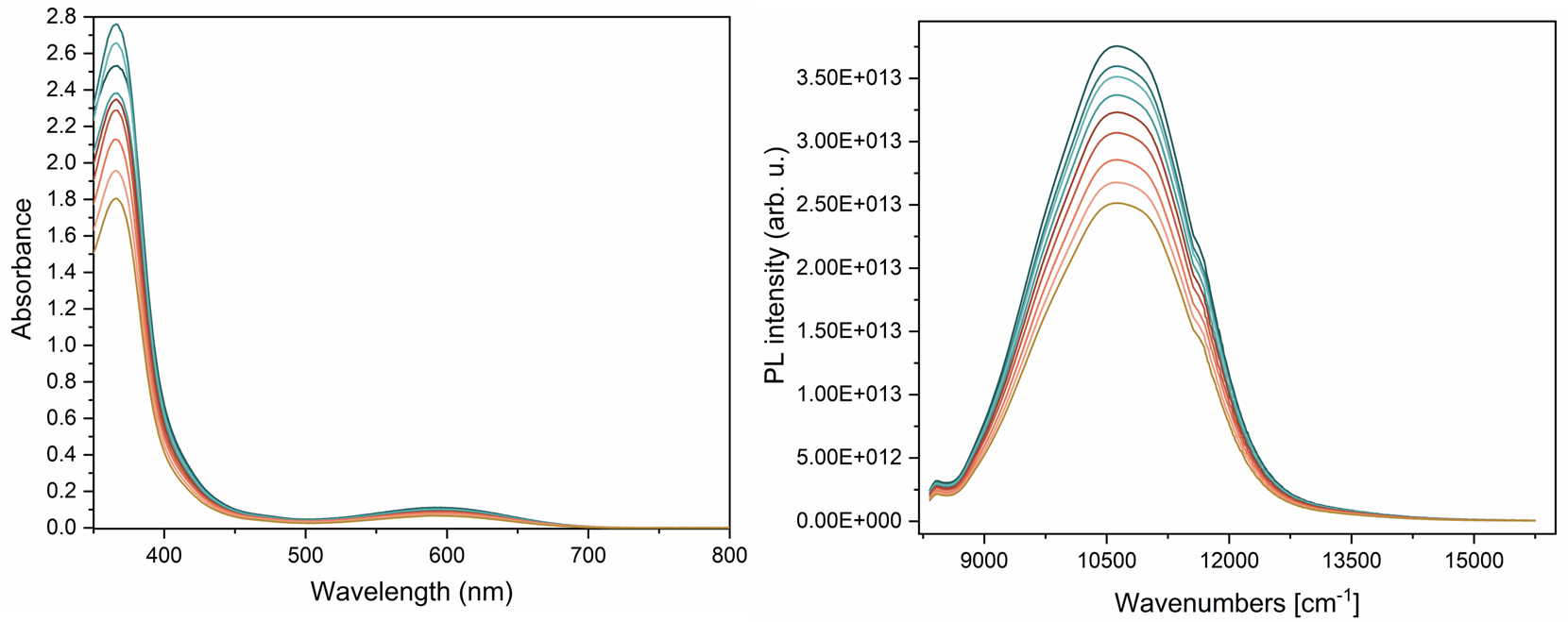


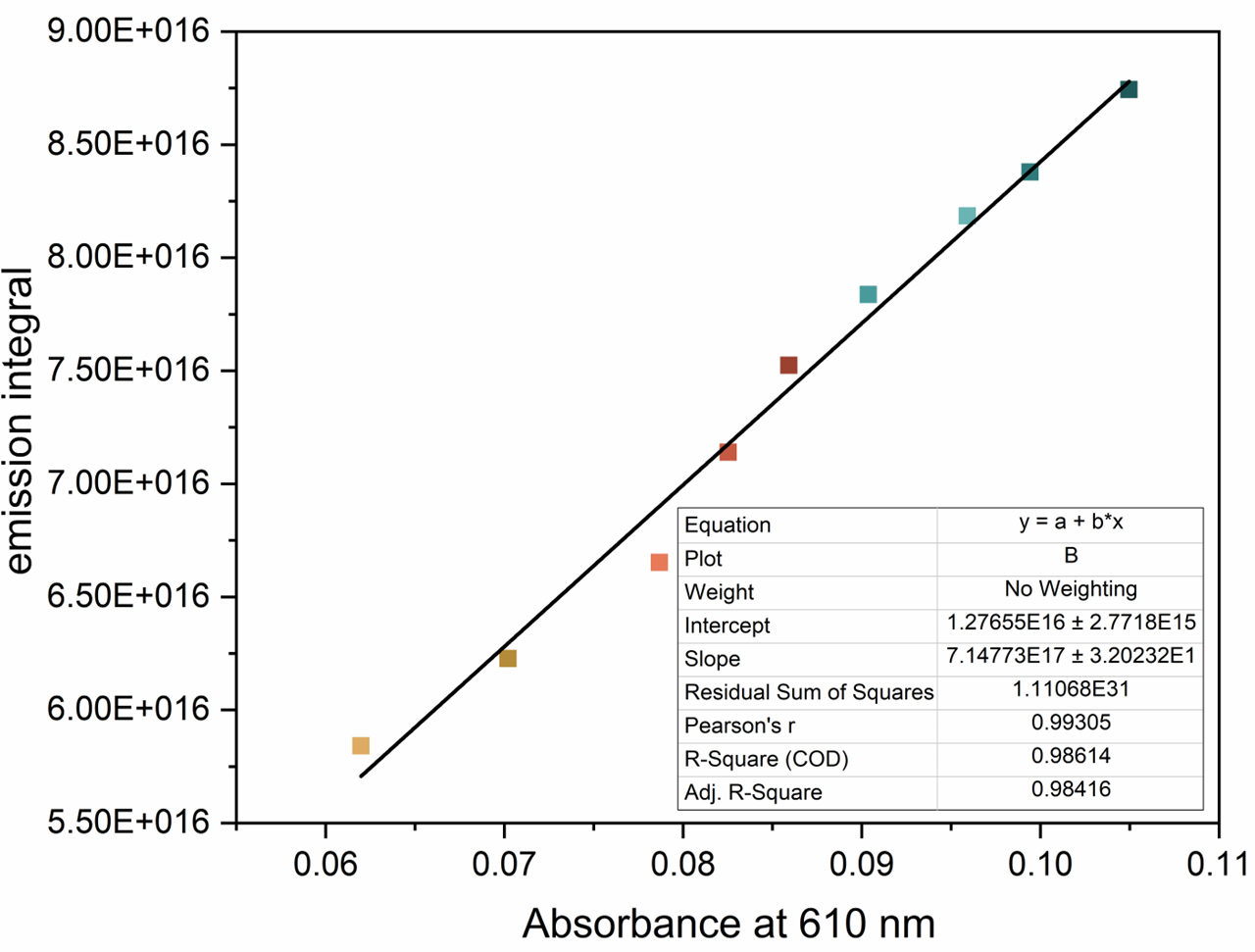


Figure S87: Raw data for luminescence quantum yield determination of **[Hom-Rh_2_]^Mes^** in deaerated dichloromethane at the excitation wavelength of 610 nm. Phosphorescence quantum yield of **[Hom-Rh_2_]^Mes^** was calculated to be 5.5 % and the upper limit of fluorescence (at 745 nm) was estimated to be <0.2 % by comparing relative intensities of the band (Figure S69b).

# **Nanosecond transient absorption measurements**


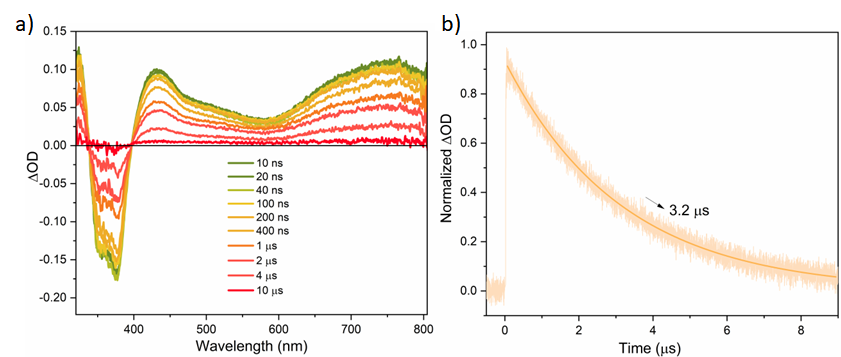


Figure S88: a) Nanosecond transient UV–Vis absorption spectra at different delay times for **[Hom-Rh_2_]^Mes^** b) Kinetic decay trace of the excited-state absorption (ESA) at 790 nm in deareated acetonitrile (laser pump pulses at 575 nm, OD_575 nm_ = 0.2, and integration time of 100 ns).


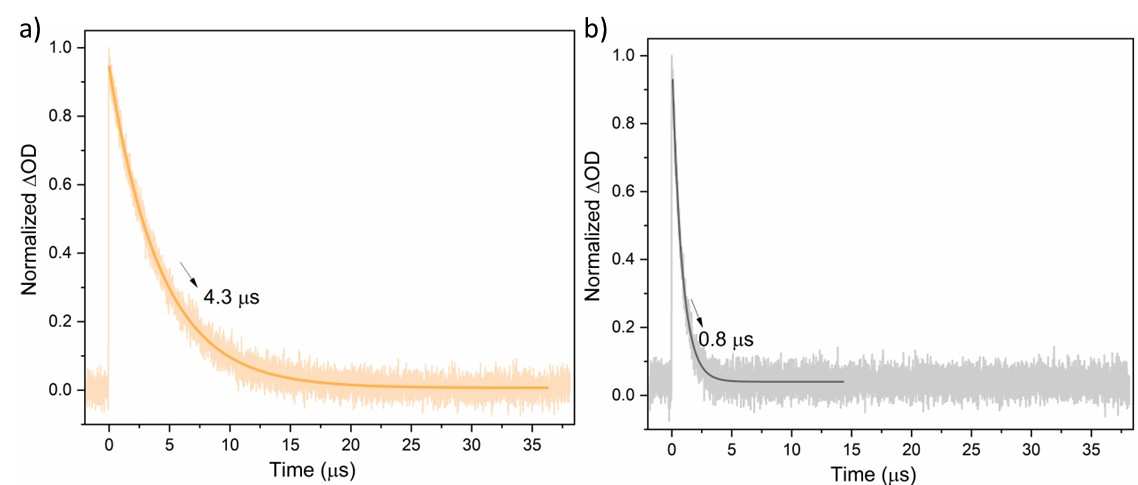


Figure S89: Kinetic decay traces of the excited-state absorption (ESA) of **[Hom-Rh_2_]^Mes^** at 450 nm (laser pump pulses at 585 nm, OD_585 nm_ = 0.1) in a) degassed CH_2_Cl_2_; b) air-equilibrated CH_2_Cl_2_.


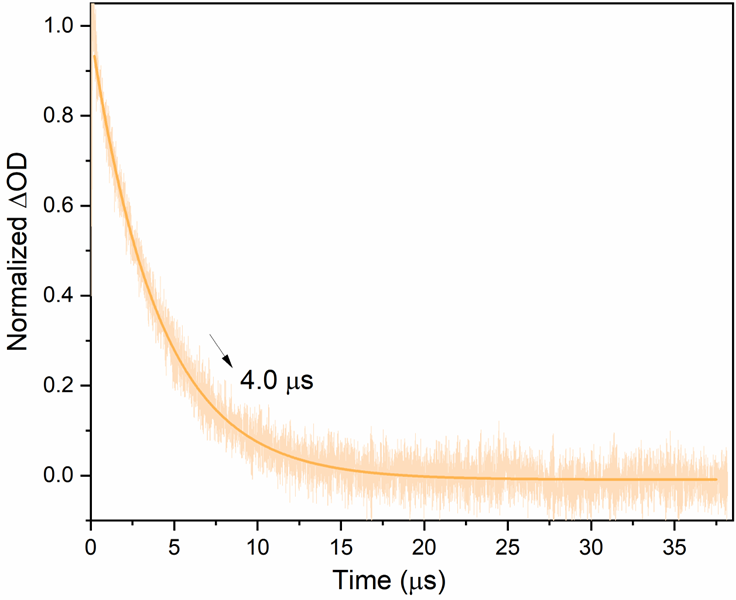


Figure S90: Phosphorescence decay of **[Hom-Rh_2_]^Mes^** after pulsed excitation at λ_exc_ = 625 nm and detected at λ_obs_ = 790 nm in deoxygenated CH_2_Cl_2_.


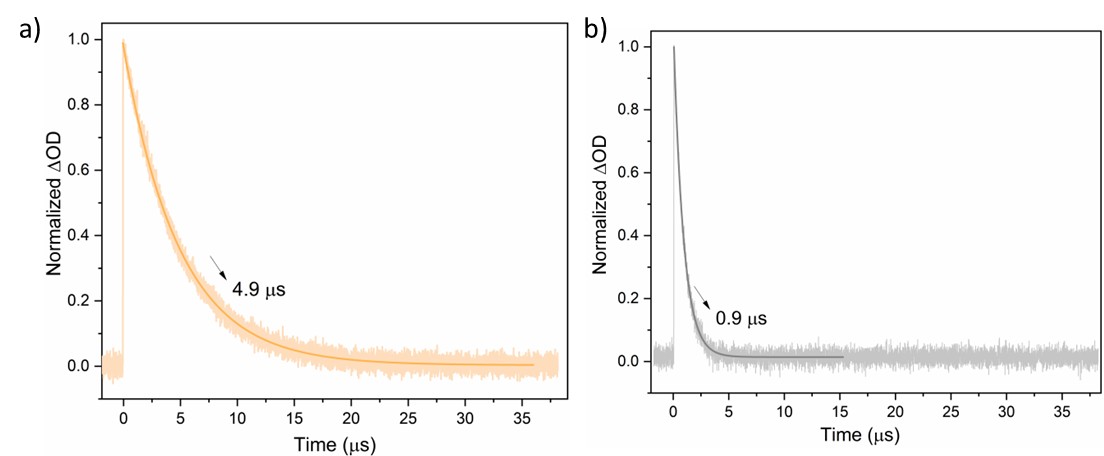


Figure S91 Kinetic decay traces of the excited-state absorption (ESA) of **[Hom-Rh_2_]*^t^*^Bu^** at 450 nm in a) degassed CH_2_Cl_2_; b) air-equilibrated CH_2_Cl_2_ (λ_exc_ = 600 nm).


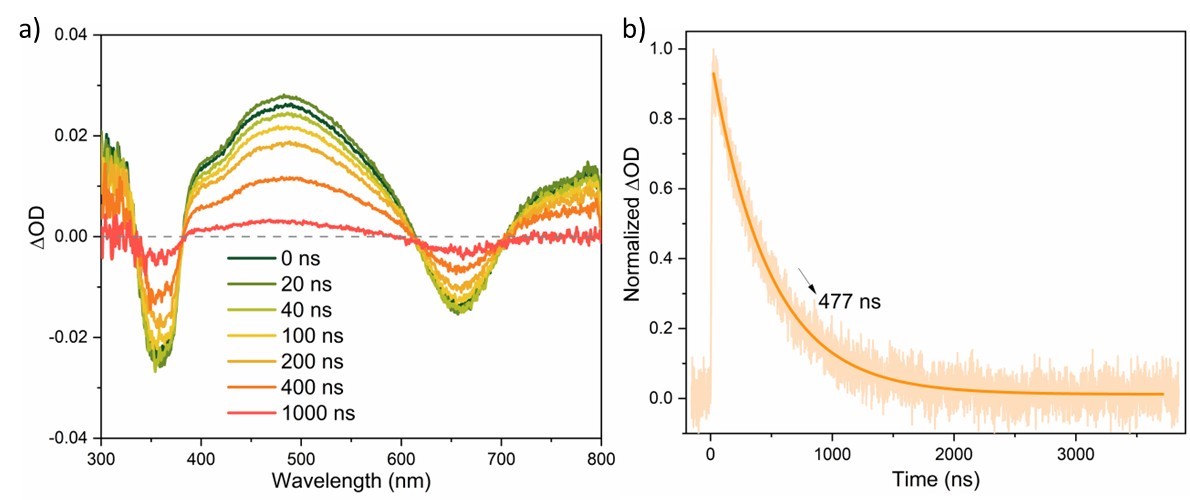


Figure S92: a) Transient UV–vis absorption spectra at different delay times for **[Het-Rh_2_]^Me^** (laser pump pulses at 625 nm, OD_625 nm_ = 0.2, and integration time of 200 ns); b) Kinetic decay of the ESA at 450 nm in degassed CH_2_Cl_2_.


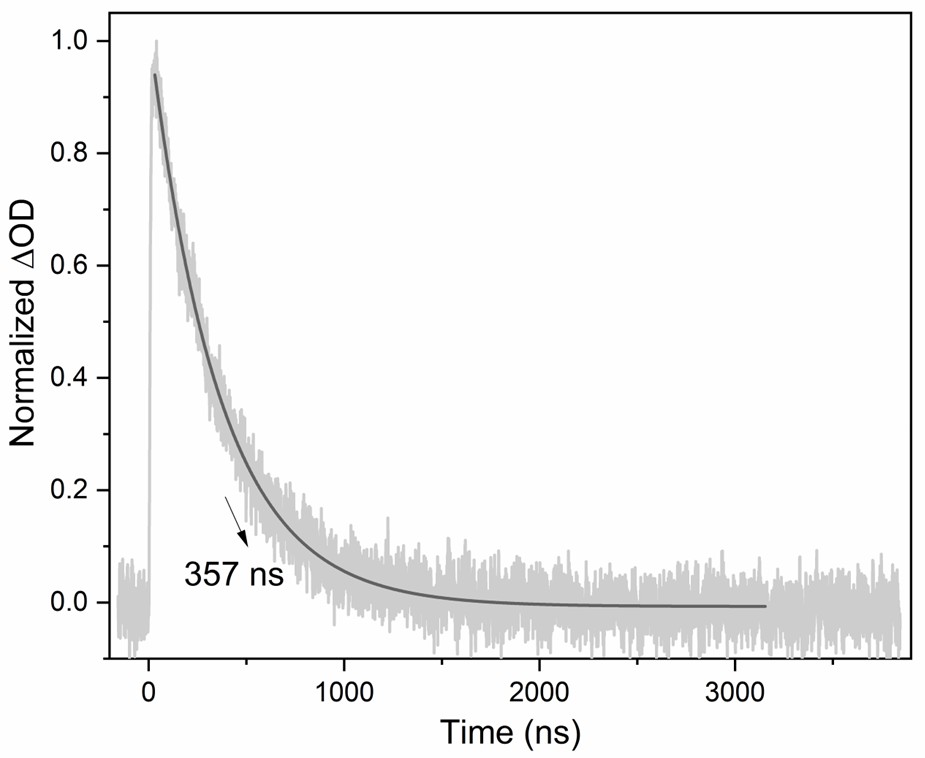


Figure S93: Kinetic decay traces of the excited-state absorption (ESA) of **[Hom-Rh_2_]^Me^** at 450 nm in air-equilibrated CH_2_Cl_2_ (λ_exc_ = 625 nm).

# **Femtosecond transient absorption measurements**


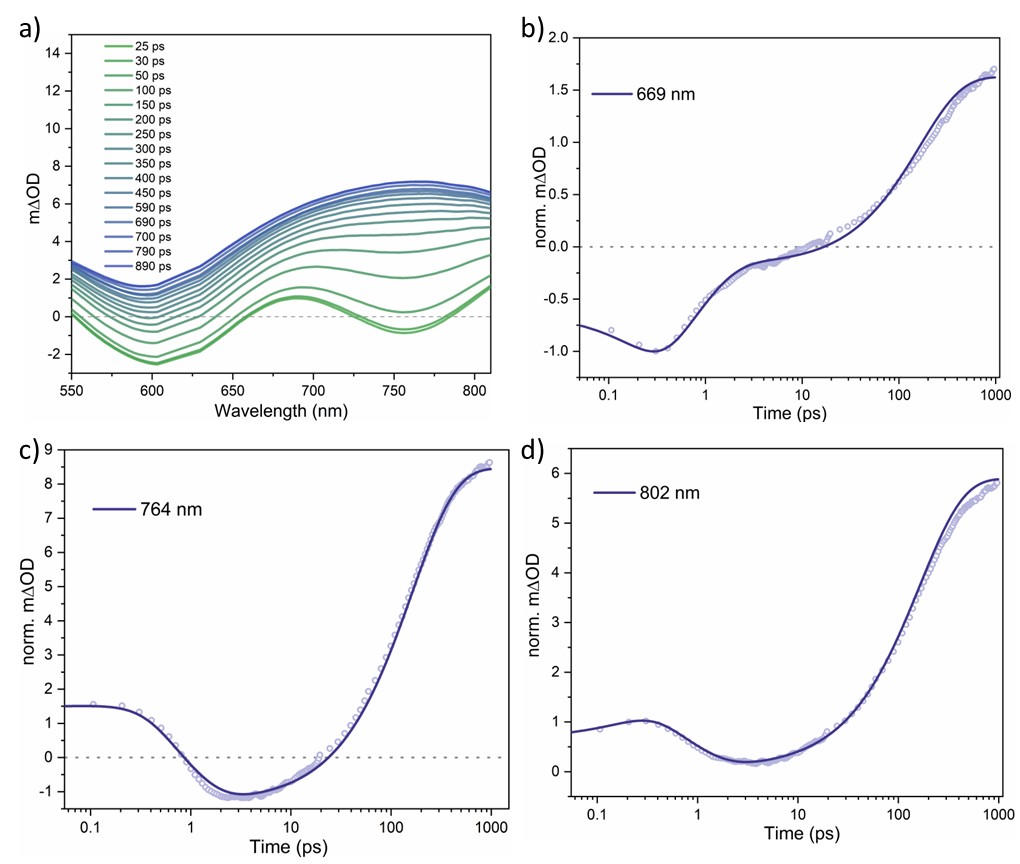


Figure S94: a) Femtosecond transient absorption spectra at longer delay times for **[Hom-Rh_2_]^tBu^** after excitation at 620 nm (OD_620 nm_ = 0.2); Single wavelength kinetic traces obtained after global analysis at b) 669 nm; c) 764 nm; d) 802 nm.


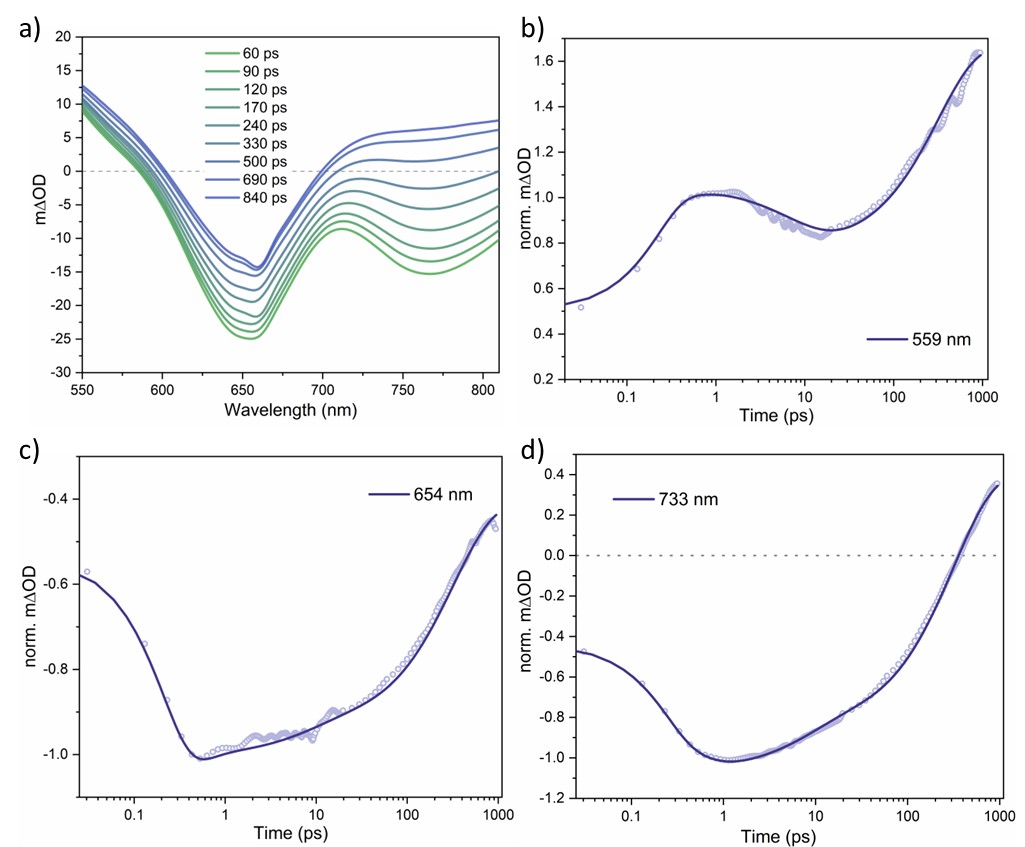


Figure S95: a) Femtosecond transient absorption spectra at longer delay times for **[Het-Rh_2_]^tBu^** after excitation at 660 nm (OD_660 nm_ = 0.2); Single wavelength kinetic traces obtained after global analysis at b) 559 nm; c) 654 nm; d) 733 nm.

Table S4: Global fitting parameters obtained from sub nanosecond transient absorption measurements.

| **Complex** | ***τ* _0_/ps (SAS1)** | ***τ _1_*/ps (SAS2)** | ***τ* _2_/ps (SAS3)** | ***τ* _3_/ps (SAS4)** |
| --- | --- | --- | --- | --- |
| [Hom-Rh_2_]*^t^*^Bu^ | 1.7 ± 0.9 | 165 ± 25 | 1.4 ± 0.7 | - |
| [Het-Rh_2_]*^t^*^Bu^ | 0.8 ± 0.4 | 320 ± 22 | 0.6 ± 0.2 | 7.7 ± 2.8 |

# **Cyclic voltammetry**


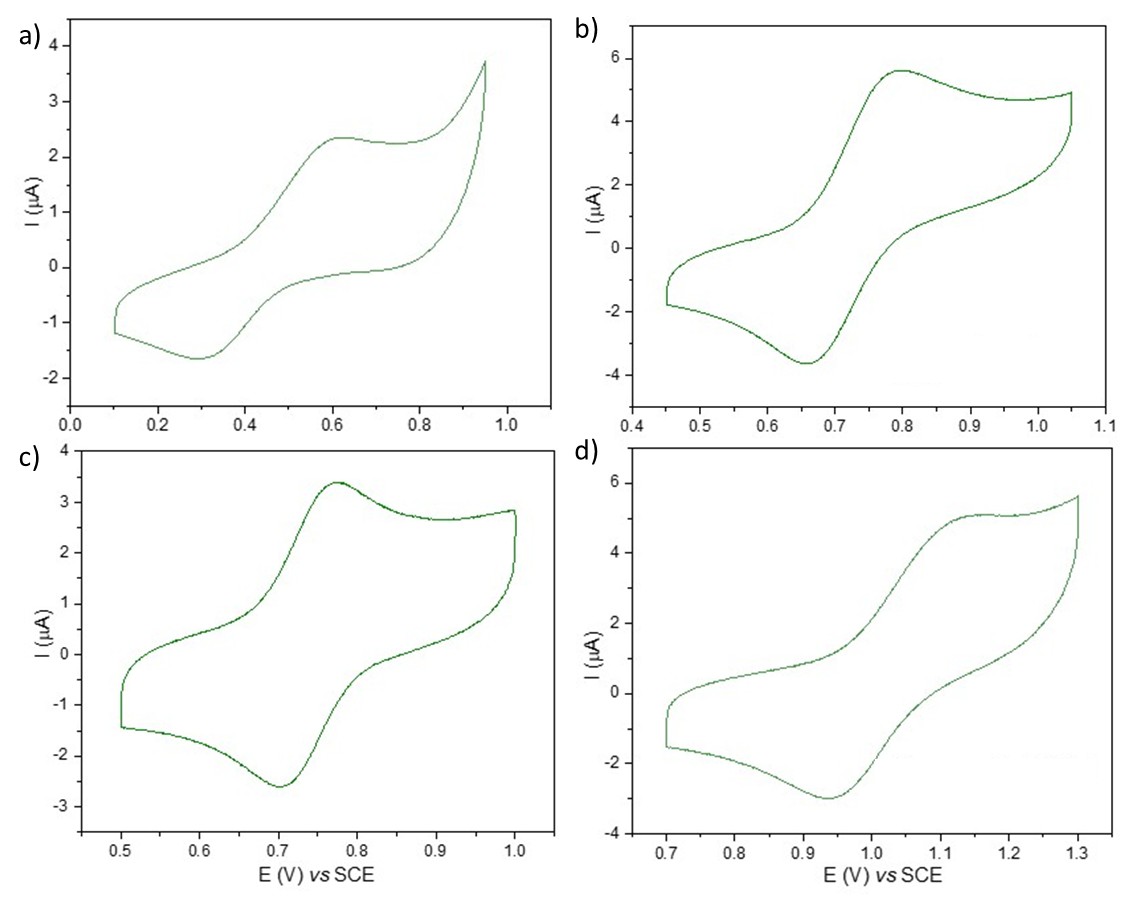


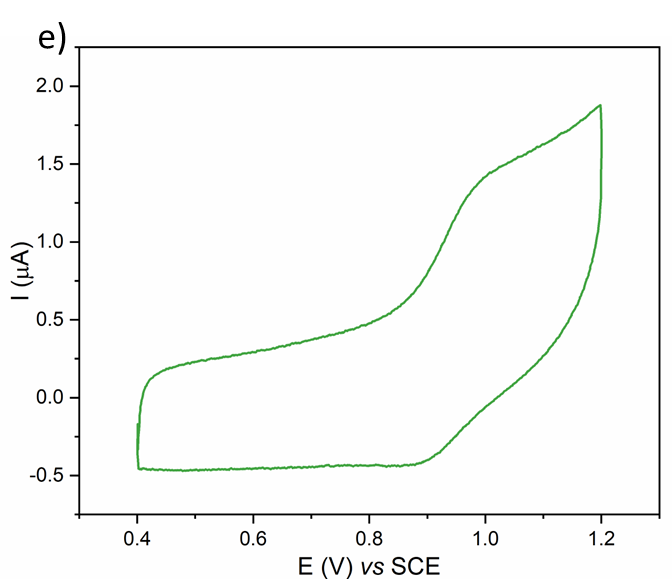


Figure S96: Cyclic voltammogram of a) **[Hom-Rh_2_]^tBu^** (E_pa_= 0.61 V vs SCE)**,** b) **[Het-Rh_2_]^tBu^** (E_1/2_ = 0.73 V vs SCE)**,** c) **[Het-Rh_2_]^Me^** (E_1/2_ = 0.74 V vs SCE), d) **[Het-Rh_2_]^tBu2^** (E_pa_ = 1.13 V vs SCE) and e) **[Hom-Rh_2_]^Mes^** (E_pa_ = 0.99 V vs SCE) in CH_2_Cl_2_ with 0.1 M of TBAPF_6_ as supporting electrolyte, recorded at a scan rate of 0.1 Vs^-1^.

# **References**

[1] V. Ritleng, D. V. Yandulov, W. W. Weare, R. R. Schrock, A. S. Hock, W. M. Davis, *J. Am. Chem. Soc.* **2004**, *126*, 6150–6163.

[2] G. R. Genov, J. L. Douthwaite, A. S. K. Lahdenperä, D. C. Gibson, R. J. Phipps, *Science* **2020**, *367*, 1246–1251.

[3] O. V. Dolomanov, L. J. Bourhis, R. J. Gildea, J. A. K. Howard, H. Puschmann, *J. Appl. Cryst.* **2009**, *42*, 339–341.

[4] G. Sheldrick, *Acta. Cryst. A* **2015**, *71*, 3–8.

[5] M. Montalti, A. Credi, L. Prodi, M. T. Gandolfi, *Handbook of photochemistry*, CRC press, **2006**.
